# Outcomes Associated With Oral Anticoagulants Plus Antiplatelets in Patients With Newly Diagnosed Atrial Fibrillation

**DOI:** 10.1001/jamanetworkopen.2020.0107

**Published:** 2020-02-26

**Authors:** Keith A. A. Fox, Priscilla Velentgas, A. John Camm, Jean-Pierre Bassand, David A. Fitzmaurice, Bernard J. Gersh, Samuel Z. Goldhaber, Shinya Goto, Sylvia Haas, Frank Misselwitz, Karen S. Pieper, Alexander G. G. Turpie, Freek W. A. Verheugt, Elizabeth Dabrowski, Kaiyi Luo, Liza Gibbs, Ajay K. Kakkar

**Affiliations:** 1 Centre for Cardiovascular Science, University of Edinburgh, Edinburgh, United Kingdom; 2 Aetion Inc, New York, New York; 3 Cardiology Clinical Academic Group Molecular & Clinical Sciences Research Institute, St George's University of London, London, United Kingdom; 4 Thrombosis Research Institute, London, United Kingdom; 5 University of Besançon, Besançon, France; 6 University of Warwick Medical School, Coventry, United Kingdom; 7 Mayo Clinic College of Medicine, Rochester, Minnesota; 8 Brigham and Women's Hospital and Harvard Medical School, Boston, Massachusetts; 9 Tokai University, Isehara, Japan; 10 Formerly Department of Medicine, Technical University of Munich, Munich, Germany; 11 Bayer HealthCare Pharmaceuticals, Berlin, Germany; 12 Duke University, Durham, North Carolina; 13 McMaster University, Hamilton, Ontario, Canada; 14 Onze Lieve Vrouwe Gasthuis (OLVG), Amsterdam, the Netherlands; 15 University College London, London, United Kingdom

## Abstract

**Importance:**

Patients with nonvalvular atrial fibrillation at risk of stroke should receive oral anticoagulants (OAC). However, approximately 1 in 8 patients in the Global Anticoagulant Registry in the Field (GARFIELD-AF) registry are treated with antiplatelet (AP) drugs in addition to OAC, with or without documented vascular disease or other indications for AP therapy.

**Objective:**

To investigate baseline characteristics and outcomes of patients who were prescribed OAC plus AP therapy vs OAC alone.

**Design, Setting, and Participants:**

Prospective cohort study of the GARFIELD-AF registry, an international, multicenter, observational study of adults aged 18 years and older with recently diagnosed nonvalvular atrial fibrillation and at least 1 risk factor for stroke enrolled between March 2010 and August 2016. Data were extracted for analysis in October 2017 and analyzed from April 2018 to June 2019.

**Exposure:**

Participants received either OAC plus AP or OAC alone.

**Main Outcomes and Measures:**

Clinical outcomes were measured over 3 and 12 months. Outcomes were adjusted for 40 covariates, including baseline conditions and medications.

**Results:**

A total of 24 436 patients (13 438 [55.0%] male; median [interquartile range] age, 71 [64-78] years) were analyzed. Among eligible patients, those receiving OAC plus AP therapy had a greater prevalence of cardiovascular indications for AP, including acute coronary syndromes (22.0% vs 4.3%), coronary artery disease (39.1% vs 9.8%), and carotid occlusive disease (4.8% vs 2.0%). Over 1 year, patients treated with OAC plus AP had significantly higher incidence rates of stroke (adjusted hazard ratio [aHR], 1.49; 95% CI, 1.01-2.20) and any bleeding event (aHR, 1.41; 95% CI, 1.17-1.70) than those treated with OAC alone. These patients did not show evidence of reduced all-cause mortality (aHR, 1.22; 95% CI, 0.98-1.51). Risk of acute coronary syndrome was not reduced in patients taking OAC plus AP compared with OAC alone (aHR, 1.16; 95% CI, 0.70-1.94). Patients treated with OAC plus AP also had higher rates of all clinical outcomes than those treated with OAC alone over the short term (3 months).

**Conclusions and Relevance:**

This study challenges the practice of coprescribing OAC plus AP unless there is a clear indication for adding AP to OAC therapy in newly diagnosed atrial fibrillation.

## Key Points

**Question:**

What outcomes are associated with combination therapy using oral anticoagulants (OAC) plus antiplatelet drugs in patients with newly diagnosed atrial fibrillation?

**Findings:**

This cohort study of 24 436 patients with de novo atrial fibrillation found that, after adjusting for baseline characteristics and comedications, patients treated with OAC plus antiplatelet drugs had significantly higher incidence rates of stroke and any bleeding event than those receiving OAC alone. Use of OAC plus antiplatelet drugs was not associated with reduced risk of experiencing acute coronary syndromes.

**Meaning:**

These findings suggest that patients with atrial fibrillation treated with OAC plus antiplatelet drugs may have significantly higher risk of stroke and bleeding compared with those receiving OAC alone.

## Teaser

This cohort study compares outcomes of treatment with oral anticoagulants plus antiplatelet drugs vs oral anticoagulants alone in patients with newly diagnosed atrial fibrillation.

## Introduction

Atrial fibrillation (AF) occurs when structural remodeling and/or electrophysiological abnormalities (eg, myocarditis or fibrosis) caused by diverse pathophysiological mechanisms (eg, hypertension or heart failure) alter atrial tissue to promote abnormal pulse wave generation and/or propagation, leading to atrial tachyarrhythmias.^1,2^ Both AF and the underlying abnormal atrial tissue predispose affected individuals to thrombus formation in the left atrium or left atrial appendage, and this can embolize to the brain and other sites. Guidelines^1,2^ recommend that patients with nonvalvular AF and CHA_2_DS_2_-VASc (cardiac failure, hypertension, age >75 years [2 points], diabetes, stroke, transient ischemic attack, or thromboembolism [2 points]–vascular disease, age >60 years, sex category female)^3,4^ risk stratification score of 2 or greater (not counting sex) should receive oral anticoagulation (OAC; vitamin K antagonist [VKA] or non-VKA OACs [NOACs]) as stroke prophylaxis regardless of symptoms; in patients with CHA_2_DS_2_-VASc score of 1, OAC may be considered. Although antiplatelet (AP) agents are not advocated for stroke prophylaxis in AF, it is known that some patients are coprescribed these drugs with OAC.^5,6,7,8^

Patients with new-onset AF may have comorbid cardiovascular disease (CVD) requiring therapy with OAC in combination with AP.^8^ Potential benefits of AP drugs in patients with CVD may be due to their favorable effects on inhibiting arterial thrombosis.^9,10^ Antiplatelet drugs may be given in combination with OAC in patients with AF after percutaneous coronary intervention, to prevent stent thrombosis, or after acute coronary syndromes (ACS).^1,2^ In patients with AF who require stenting, guidelines recommend concurrent AP plus OAC for up to 1 year and, in those at risk for stroke, OAC alone thereafter.^11,12^

In the large observational Global Anticoagulant Registry in the Field–Atrial Fibrillation (GARFIELD-AF) study,^5^ approximately 1 in 8 patients with AF at risk for stroke received AP therapy concomitantly with OAC, irrespective of whether they had a confirmed indication for AP. Because the balance of risk vs benefit with combination therapy using OAC plus AP is not well defined, the present study investigated baseline characteristics and outcomes of patients who were newly prescribed OAC plus AP therapy at the time of diagnosis of AF, using data from GARFIELD-AF.

## Methods

### Study Design and Participants

The GARFIELD-AF study design and main findings have been reported previously.^5,13^ The registry is a prospective, multicenter, observational study of adults aged 18 years and older with recently diagnosed nonvalvular AF and at least 1 risk factor for stroke. Patients were recruited from a range of representative care settings in each country between December 2009 and October 2017. No specific treatments, tests, or procedures were mandated by the study protocol. Decisions to initiate, continue, or change treatment were solely at the discretion of treating physicians. Patients with a transient reversible cause of AF and those for whom follow-up was not envisaged or possible were excluded.^2^

Independent ethics committee and hospital-based institutional review board approvals were obtained for the GARFIELD-AF study, including all subsequent analyses of the data. The registry was conducted in accordance with the principles of the Declaration of Helsinki,^14^ local regulatory requirements, and the International Conference on Harmonisation–Good Pharmacoepidemiological and Clinical Practice guidelines. Written informed consent was obtained from all study participants. This study followed the Strengthening the Reporting of Observational Studies in Epidemiology (STROBE) reporting guideline.

### Data Capture

In this prospective observational study, outcomes were captured by electronic case report forms. Submitted data were examined for completeness and accuracy by the coordinating center (Thrombosis Research Institute, London, United Kingdom), and data queries were sent to study sites. An audit and quality control program was implemented that included source documentation (20% of all electronic case report forms were monitored against source records).^15^

Baseline characteristics collected at study entry included medical history, care setting, type of AF, date and method of diagnosis of AF, symptoms, antithrombotic treatment (VKAs, NOACs, and AP), as well as all cardiovascular drugs. Race was classified by the investigator in agreement with the patient.^13^ Vascular disease included coronary artery disease (CAD) with a history of ACS and/or peripheral artery disease. Chronic kidney disease was classified according to National Kidney Foundation guidelines into moderate to severe (stages 3-5), mild (stages 1 and 2), or none. Data on components of the CHA_2_DS_2_-VASc risk stratification scheme were collected and calculated retrospectively since patients’ inclusion in the registry was decided by physicians’ clinical judgment. Collection of follow-up data occurred at 4-month intervals up to 24 months. Data for the present investigation were extracted from the study database in October 2017 and analyzed from April 2018 to June 2019.

In the present analysis, clinical outcomes and bleeding risk were investigated and compared in patients with de novo AF who received either OAC plus AP or OAC alone over 3 and 12 months.

### Statistical Analysis

Patients who were prescribed AP drugs, defined as aspirin or P2Y_12_-type ADP receptor inhibitors in combination with OAC were compared with those who did not receive concomitant AP therapy. To reduce risk of bias due to patient selection, patients who had previously taken OACs or AP drugs were excluded from this analysis, as were those prescribed VKA and a NOAC. Subgroup analyses were performed in patients classified as having low and high risk for AF-related stroke (defined as CHA_2_DS_2_-VASc score <2 and ≥2, respectively).

An intent-to-treat analysis was calculated using Cox proportional hazards regression to estimate multivariate adjusted hazard ratios (aHR) and 95% confidence intervals for the study end points of all-cause mortality, myocardial infarction (MI) or ACS, stroke, stroke or systemic embolism, any bleeding, major bleeding, major bleeding and hemorrhagic stroke, and major or nonmajor clinically relevant bleeding (see study design article^13^ for definitions of these events). Models were adjusted for 40 covariates (eTable 1 in the Supplement) reflecting demographic and clinical characteristics, medical history, and concomitant medication at registry entry. The covariates included all documented vascular indications for AP therapy. As a falsification analysis, the same approach was used to investigate the influence of supplemental AP therapy on an implausible end point such as death unrelated to cardiovascular disease. For each adverse outcome analyzed, patients were censored on first occurrence of that event, loss to follow-up, death, or reaching 90 days of follow-up for 3-month analyses and 365 days for 12-month analyses. Additionally, a propensity score model including the same set of covariates was developed and patients treated with AP drugs were matched 1:1 to patients not treated with AP drugs to create balanced cohorts, in which Cox regression was used to estimate HRs and 95% confidence intervals. Patients with missing values were included in the analysis.

A supplementary as-treated analysis was performed for all study end points using Cox proportional hazard regression to estimate multivariate adjusted HRs and 95% confidence intervals in the full analysis population and in the propensity score–matched cohorts that we have described. Patients were censored on occurrence of any outcome, loss to follow-up, death, discontinuation of therapy, or interruption of index therapy plus a 7-day risk window, addition or change of the index AP regimen, or reaching 365 days of follow-up.

An α of .05 (2-tailed) was used for statistical significance. All analyses were conducted using Aetion Evidence Platform version 3.13 (Aetion Inc).

## Results

### Baseline Patient Characteristics

**Figure 1.**
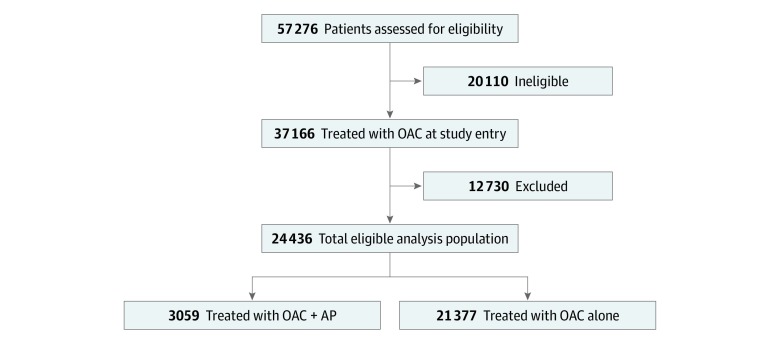
Patients’ Disposition in the Study

In total, 57 276 patients were enrolled in GARFIELD-AF between December 2009 and October 2017. After all inclusion and exclusion criteria were applied, the final number of eligible patients newly treated with OAC plus AP or OAC alone at registry entry was 24 436 (13 438 [55.0%] male; median [interquartile range] age, 71 [64-78] years) (Figure 1). Of these, 3059 patients (12.5%) composed the OAC plus AP group, and 21 377 (87.2%) composed the OAC alone group. Both patients who received OAC plus AP and those who received OAC alone had a median age of 71 years. The majority of patients (84.4%) had a moderate to high risk of stroke (CHA_2_DS_2_-VASc score ≥2); in the overall population, the median (interquartile range) CHA_2_DS_2_-VASc score was 3 (2-4) (Table).

**Table.** Clinical Characteristics of Patients With Atrial Fibrillation Treated With OAC Plus AP or OAC Alone for Stroke Prophylaxis

Parameter	OAC Plus AP (n = 3059)	OAC Alone (n = 21 377)
Male, No. (%)	1925 (62.9)	11 513 (53.9)
Age, mean (SD), y	69.8 (10.6)	70.3 (11.0)
Body mass index, mean (SD)^a^	28.2 (5.6)	27.9 (5.8)
Blood pressure, systolic/diastolic, mean	132.9/79.7	134.6/80.5
Pulse rate, mean (SD), beats/min	90.9 (26.9)	91.4 (26.7)
Left ventricular ejection fraction, mean (SD), %	52.8 (13.8)	56.7 (12.4)
CHA_2_DS_2_-VASc score, median (IQR)	3 (2-4)	3 (2-4)
Medical history, No. (%)		
Congestive heart failure	770 (25.2)	3587 (16.8)
Coronary artery disease	1195 (39.1)	2100 (9.8)
Acute coronary syndrome	673 (22.0)	926 (4.3)
Carotid occlusive disease	146 (4.8)	437 (2.0)
Deep vein thrombosis or pulmonary embolism	106 (3.5)	609 (2.8)
Stroke or transient ischemic attack	503 (16.4)	1902 (8.9)
Bleeding	84 (2.7)	346 (1.6)
Hypertension	2468 (80.7)	16 290 (76.2)
Hypercholesterolemia	1501 (49.1)	7771 (36.4)
Diabetes, type 1 or 2	923 (30.2)	4245 (19.9)
Chronic kidney disease, moderate to severe	406 (13.3)	2099 (9.8)

Abbreviations: AP, antiplatelet; CHA_2_DS_2_-VASc, cardiac failure, hypertension, age greater than 75 years (2 points), diabetes, stroke, transient ischemic attack, or thromboembolism (2 points)–vascular disease, age greater than 60 years, sex category female; IQR, interquartile range; OAC, oral anticoagulant.

^a^ Calculated as weight in kilograms divided by height in meters squared.

Compared with patients receiving OAC alone, those who received OAC plus AP therapy had a greater prevalence of cardiovascular indications for AP, including ACS (22.0% vs 4.3%), CAD (39.1% vs 9.8%), and carotid occlusive disease (4.8% vs 2.0%). These patients also had a higher prevalence of cardiovascular conditions such as congestive heart failure (25.2% vs 16.8%), history of hypertension (80.7% vs 76.2%), history of hypercholesterolemia (49.1% vs 36.4%), and history of bleeding (2.7% vs 1.6%) as well as severe renal disease (13.3% vs 9.8%) and diabetes (30.2% vs 19.9%). A higher proportion of patients receiving OAC plus AP were male (Table).

Among 20 687 patients at high risk of stroke (CHA_2_DS_2_-VASc score ≥2), 2735 (13.2%) received OAC plus AP therapy. This subpopulation had a higher prevalence of indications for AP, cardiovascular conditions (except congestive heart failure), severe renal disease, and diabetes and higher likelihood of receiving cardiovascular medications than their counterparts receiving OAC alone. Within this high-risk subpopulation, median (interquartile range) CHA_2_DS_2_-VASc score for those prescribed OAC plus AP and OAC alone was 4 (3-4) and 3 (2-4), respectively (eTable 2 in the Supplement).

### Clinical Outcomes at 12 Months

**Figure 2.**
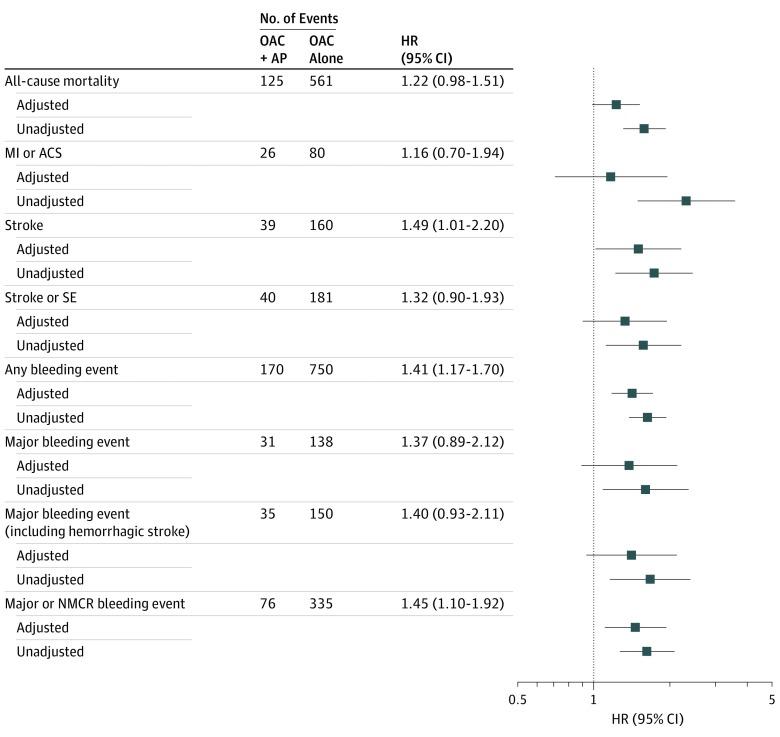
Relative Risk (Hazard Ratios [HRs], Unadjusted and Adjusted) for Study Outcomes in Patients With Newly Diagnosed Atrial Fibrillation Treated With Oral Anticoagulants (OAC) Plus Antiplatelet Drugs (AP) or OAC Alone (Reference) Over 12 Months (Intent-to-Treat Analyses) Hazard ratios were adjusted for 40 covariates as shown in eTable 1 in the Supplement. AP indicates antiplatelet drugs; and OAC, oral anticoagulants. ACS indicates acute coronary syndromes; MI, myocardial infarction; NMCR, nonmajor, clinically relevant; and SE, systemic embolism.

Unadjusted and adjusted HRs for outcome events over 12 months are displayed in Figure 2. After adjustment for 40 covariates, including baseline medications, patients treated with OAC plus AP had significantly higher incidence rates of stroke (aHR, 1.49; 95% CI, 1.01-2.20) and any bleeding event (aHR, 1.41; 95% CI, 1.17-1.70) as well as composite end points death or stroke (aHR, 1.27; 95% CI, 1.05-1.55) and death, stroke, or major bleeding (aHR, 1.32; 95% CI, 1.10-1.59) than those treated with OAC alone. Moreover, patients prescribed OAC plus AP did not show evidence of reduced all-cause mortality (aHR, 1.22; 95% CI, 0.98-1.51), stroke and/or systemic embolism (aHR, 1.32; 95% CI, 0.90-1.93), and major bleeding events including hemorrhagic stroke (aHR, 1.40; 95% CI, 0.93-2.11). Risk of ACS was not reduced in patients taking OAC plus AP compared with OAC alone (aHR, 1.16; 95% CI, 0.70-1.94). Hazard ratios generated from the propensity score model were similar for each outcome, although precision was slightly reduced owing to smaller sample size after matching 1:1 (results not shown).

Within the subpopulation of patients at high risk for stroke, the aHRs and HRs generated from the propensity score model were similar to those seen in the overall population for all outcomes (eg, stroke: aHR 1.55; 95% CI, 1.04-2.30; any bleeding event: aHR, 1.42; 95% CI, 1.17-1.72; major and nonmajor clinically relevant bleeding: aHR, 1.50; 95% CI, 1.13-1.99; death or stroke: aHR, 1.27; 95% CI, 1.04-1.56; death, stroke, or major bleeding: aHR, 1.33; 95% CI, 1.10-1.60). No reductions in risk of other clinical outcomes with OAC plus AP vs OAC alone were noted, including ACS (eFigure 1 in the Supplement).

### Clinical Outcomes at 3 Months

**Figure 3.**
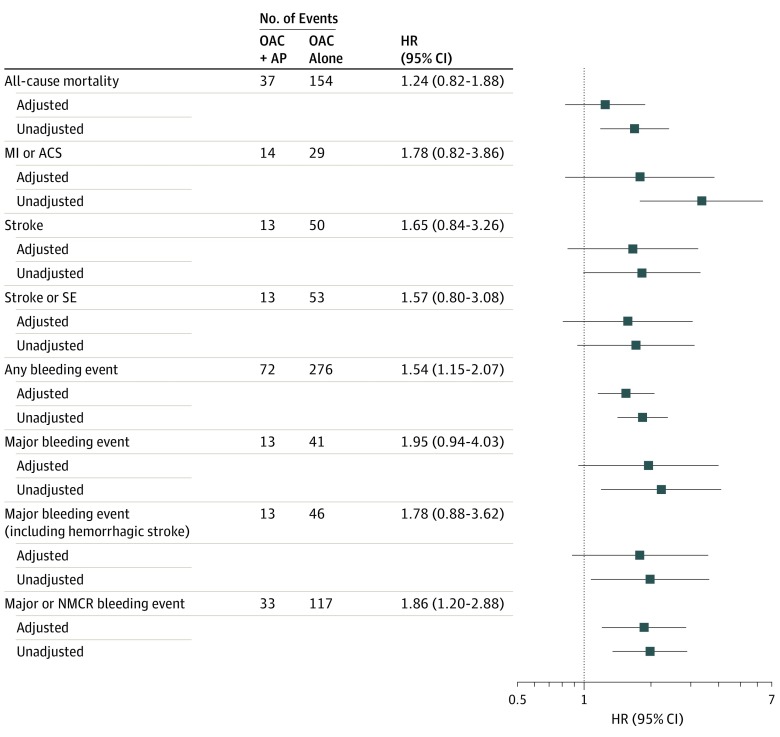
Relative Risk (Hazard Ratios [HRs], Unadjusted and Adjusted) for Study Outcomes in Patients With Newly Diagnosed Atrial Fibrillation Treated With Oral Anticoagulants (OAC) Plus Antiplatelet Drugs (AP) or OAC Alone (Reference) Over 3 Months (Intent-to-Treat Analyses) Hazard ratios were adjusted for 40 covariates as shown in eTable 1 in the Supplement. ACS indicates acute coronary syndromes; MI, myocardial infarction; NMCR, nonmajor, clinically relevant; and SE, systemic embolism.

Patients treated with OAC plus AP at registry entry had numerically higher rates of all clinical outcomes than those treated with OAC alone over 3 months (Figure 3). However, only any bleeding (aHR, 1.54; 95% CI, 1.15-2.07), major and nonmajor clinically relevant bleeding (aHR, 1.86; 95% CI, 1.20-2.88), and death, stroke, or major bleeding (aHR, 1.48; 95% CI, 1.07-2.06) exhibited statistically significant increases. Similar patterns were seen among the subgroup of patients at high risk for stroke at 3 months of follow-up (eFigure 2 in the Supplement).

### As-Treated Analyses

As-treated analyses, in which patients were censored at the time of discontinuation or change of initial treatment, resulted in similar findings to the primary intent-to-treat analyses (data not shown).

### Falsification Analysis

Among 2541 patients treated with OAC plus AP and 17 673 patients treated with OAC alone who died from causes unrelated to cardiovascular disease over the first 12 months, risk per 1000 patients was estimated to be 9.45 and 11.32, respectively (aHR for event, 0.76; 95% CI, 0.48-1.22) (eTable 3 in the Supplement).

## Discussion

In this prospective registry of patients with newly diagnosed AF receiving anticoagulant therapy, the majority (87.2%) was treated with OAC alone, whereas 1 in 8 individuals (12.5%) received OAC plus AP. Patients prescribed OAC plus AP had a higher burden of cardiovascular indications for AP therapy such as ACS, CAD, and carotid occlusive disease, as well as a range of cardiovascular conditions that AP drugs are not known to ameliorate, including hypertension, diabetes (an independent risk factor for MI and stroke^16,17^), and history of bleeding. During the observation period, patients treated with OAC plus AP experienced a higher incidence of adverse outcomes such as stroke, bleeding, and death than those treated with OAC alone over the longer term (12 months) and shorter term (3 months), both before and after adjusting for baseline conditions and comedications. Moreover, patients receiving OAC plus AP did not achieve lower risk of ACS vs patients who were prescribed OAC alone. Reducing early risk is challenging because it is known that the rate of cardiovascular mortality is highest during the first 1 month after diagnosis of AF.^18^

Patients presenting with AF and moderate to high risk for AF-related stroke (CHA_2_DS_2_-VASc score ≥2)^19,20^ are usually offered anticoagulant therapy. In low-risk patients (CHA_2_DS_2_-VASc ≤1), neither OAC nor AP is recommended because the potential for causing bleeding as an adverse effect could exceed the beneficial effects of preventing stroke. Conversely, in higher-risk patients, preventing stroke is a treatment priority, albeit at a cost of some increase in risk of bleeding. Antiplatelet drugs such as aspirin and clopidogrel, either alone or in combination (dual AP therapy), have been demonstrated to be less efficacious than OACs at preventing stroke in patients with AF and can cause similar or higher rates of bleeding.^21,22,23,24,25,26,27,28,29,30,31^ Therefore, AP drugs are not routinely recommended for stroke prophylaxis in patients with AF.

Combining antithrombotic drugs increases their potential to cause bleeding. In a Danish registry study of 82 854 patients with AF with follow-up of more than 3 years, drug-induced nonfatal or fatal bleeding was seen in 11.4%; the risk was lowest in patients who took aspirin or warfarin monotherapy, slightly higher for clopidogrel, and markedly higher (more than 3-fold compared with warfarin alone) for dual warfarin plus clopidogrel and triple therapy using warfarin, aspirin, and clopidogrel.^32^ These findings were confirmed in patients with AF receiving multiple antithrombotic drugs, including triple therapy, following MI or percutaneous coronary intervention.^33,34^ The same researchers retrospectively studied patients with AF with coexisting stable CAD and found that risk of recurrent coronary events or thromboembolism was the same for VKA plus aspirin or clopidogrel as for VKA alone, whereas the risk for bleeding increased when either AP drug was given concurrently with VKA.^35^

Hsu et al^36^ analyzed 200 000 outpatients with AF at risk for stroke enrolled in the American College of Cardiology’s Practice Innovation and Clinical Excellence (PINNACLE) registry and identified factors associated with prescribing aspirin alone over OAC that included hypertension, dyslipidemia, CAD, prior MI, angina, recent coronary artery bypass graft, and peripheral artery disease. Patients prescribed OAC, on the other hand, were more often male or had higher body mass index, prior stroke or transient ischemic attack, or heart failure.

Steinberg and colleagues^6^ looked at patterns of use and associated risks of coprescribing antithrombotic drugs in a cohort of 10 000 patients enrolled in the US-based Outcomes Registry for Better Informed Treatment of Atrial Fibrillation (ORBIT-AF) study. Patients receiving aspirin plus OAC were more likely to be male (66% vs 53%; *P* < .0001) and had more comorbid illness, although 39% did not have atherosclerotic disease. Major bleeding and bleeding hospitalizations were significantly greater (by approximately 50%) in patients receiving aspirin plus OAC than in those receiving OAC alone. Overall rates of ischemic events were low. These researchers suggested that adding aspirin therapy to OAC may not be worth the risk in AF, in particular in patients who do not have a convincing indication for aspirin, such as manifest atherosclerosis.

Several clinical trials^37,38,39^ have investigated the efficacy and safety of add-on AP therapy in patients with AF receiving OACs. In the Apixaban for Reduction in Stroke and Other Thromboembolic Events in Atrial Fibrillation (ARISTOTLE) study^37^ conducted in more than 18 000 patients with AF at risk of stroke, apixaban exerted comparable favorable effects on preventing stroke, systemic embolism, and mortality and caused less major bleeding than warfarin irrespective of whether aspirin was concomitantly used, including in subgroups of patients with arterial disease. Comparable findings were reported for the pivotal Effective Anticoagulation with Factor Xa Next Generation in Atrial Fibrillation (ENGAGE-AF) study^38^ of edoxaban vs warfarin and Rivaroxaban Once Daily Oral Direct Factor Xa Inhibition Compared with Vitamin K Antagonism for Prevention of Stroke and Embolism Trial in Atrial Fibrillation (ROCKET AF) trial^39^ of rivaroxaban vs warfarin in AF.

### Strengths and Limitations

To our knowledge, GARFIELD-AF is the largest international prospective registry in AF with extensive quality control measures providing reassurance for accuracy of results.^5,13,15^ Although the data were adjusted for an extensive range of clinical and medical history variables known to influence outcomes, differences between patients treated with either OAC plus AP or OAC alone may be subject to unmeasured confounders related to treatment selection by physicians. Indeed, although we were able to analyze clinical scenarios significantly associated with likelihood of receiving comedication with OAC plus AP, treating physicians’ actual reasons for adopting this strategy in individual patients were not recorded. On the other hand, our falsification analysis suggests lack of appreciable bias in this research. Patients included in the present analysis had not received prior OAC or AP therapy.

## Conclusions

This study suggests that patients with AF at risk for stroke who receive OAC do not require supplemental AP therapy unless there are clear indications for these medications, such as intercurrent ACS or as adjunct to percutaneous coronary intervention. However, this study shows that approximately 1 in 8 patients who are not in this category do receive OAC plus AP. In this study, patients receiving add-on AP therapy had more cardiovascular complications than those given OAC alone, even after adjusting for all baseline risk factors and medications. These findings challenge the clinical practice of combining OAC and AP therapy for stroke prevention in patients with de novo AF.

## Notes

Supplement. **eTable 1.** List of Covariates Included for Calculation of Multivariate Adjusted Hazard Ratios (aHR) **eTable 2.** Baseline Clinical Characteristics of Patients at High Risk for Stroke (CHA₂DS₂-VASc score ≥2) **eTable 3.** Falsification Analysis—Non-CV Mortality Calculated Over 12-Month Follow-up **eFigure 1.** Relative Risk (Hazard Ratios, Unadjusted and Adjusted) for Study Outcomes in High-Risk AF Patients (CHA2DS2-VASc Score ≥2) Treated With OAC Plus AP or OAC Alone (Reference) Over 12 Months (Intent-to-Treat Analyses) **eFigure 2.** Relative Risk (Hazard Ratios, Unadjusted and Adjusted) for Study Outcomes in High-Risk AF Patients (CHA2DS2-VASc Score ≥2) Treated with OAC Plus AP or OAC Alone (Reference) Over 3 Months (Intent-to-Treat Analyses) **eAppendix. **Group Information
