# Supplementary material for: Outcomes Associated With Oral Anticoagulants Plus Antiplatelets in Patients With Newly Diagnosed Atrial Fibrillation
Source: JAMA Netw Open. 2020 Feb 26;3(2):e200107. doi: 10.1001/jamanetworkopen.2020.0107 (PMC7137686; doi:10.1001/jamanetworkopen.2020.0107)
Supplement: Supplement. — eTable 1. List of Covariates Included for Calculation of Multivariate Adjusted Hazard Ratios (aHR) eTable 2. Baseline Clinical Characteristics of Patients at High Risk for Stroke (CHA₂DS₂-VASc score ≥2) eTable 3. Falsification Analysis—Non-CV Mortality Calculated Over 12-Month Follow-up eFigure 1. Relative Risk (Hazard Ratios, Unadjusted and Adjusted) for Study Outcomes in High-Risk AF Patients (CHA2DS2-VASc Score ≥2) Treated With OAC Plus AP or OAC Alone (Reference) Over 12 Months (Intent-to-Treat Analyses) eFigure 2. Relative Risk (Hazard Ratios, Unadjusted and Adjusted) for Study Outcomes in High-Risk AF Patients (CHA2DS2-VASc Score ≥2) Treated with OAC Plus AP or OAC Alone (Reference) Over 3 Months (Intent-to-Treat Analyses) eAppendix. Group Information [file jamanetwopen-3-e200107-s001.pdf]

Supplementary Online Content

Fox KAA, Velentgas P, Camm AJ, et al; GARFIELD-AF Investigators. Outcomes associated with oral anticoagulants plus antiplatelets in patients with newly diagnosed atrial fibrillation. *JAMA Netw Open*. 2020;3(2):e200107. doi:10.1001/jamanetworkopen.2020.0107

eTable 1. List of Covariates Included for Calculation of Multivariate Adjusted Hazard Ratios (aHR)

eTable 2. Baseline Clinical Characteristics of Patients at High Risk for Stroke (CHA_2DS_2-VASc score ≥2)

eTable 3. Falsification Analysis—Non-CV Mortality Calculated Over 12-Month Follow-up

eFigure 1. Relative Risk (Hazard Ratios, Unadjusted and Adjusted) for Study Outcomes in High-Risk AF Patients (CHA_2DS_2-VASc Score ≥2) Treated With OAC Plus AP or OAC Alone (Reference) Over 12 Months (Intent-to-Treat Analyses)

eFigure 2. Relative Risk (Hazard Ratios, Unadjusted and Adjusted) for Study Outcomes in High-Risk AF Patients (CHA_2DS_2-VASc Score ≥2) Treated with OAC Plus AP or OAC Alone (Reference) Over 3 Months (Intent-to-Treat Analyses)

eAppendix. Group Information

This supplementary material has been provided by the authors to give readers additional information about their work.

© 2020 Fox KAA et al. *JAMA Network Open.*

eTable 1. List of Covariates Included for Calculation of Multivariate Adjusted Hazard Ratios (aHR)

Patient characteristics<br>Sex
Age
Race/ethnicity
Pulse rate (bpm)
Diastolic blood pressure (mmHg)
Systolic blood pressure (mmHg)
Medical history
Heart failure
Acute coronary syndromes (MI or unstable angina)
Coronary artery disease
Diabetes mellitus (type 1 or 2)
Bleeding (history)
Carotid occlusive disease
Coronary artery bypass graft
Dementia
Hypercholesterolemia
Hypertension (history)
Pulmonary embolism or deep vein thrombosis
Stroke/transient ischemic attack
Systemic embolization (history)
Cirrhosis
Renal disease severe

© 2020 Fox KAA et al. *JAMA Network Open.*

Alcohol consumption
Smoker
Medication use at study entry
NSAIDs/COX-2 inhibitor (prior use)
Calcium channel blockers
Proton pump inhibitors
Digoxin
Statin
Beta blocker
Oral antidiabetic agent
Digitalis
Alfa blocker
Angiotensin-converting enzyme inhibitor (ACEi)
Angiotensin II receptor blocker (ARB)
Aldosterone antagonist
Nitrates
Insulin
Loop or other diuretics
Bisoprolol
Year/quarter study entry

© 2020 Fox KAA et al. *JAMA Network Open.*

eTable 2. Baseline Clinical Characteristics of Patients at High Risk for Stroke (CHA_2DS_2-VASc score ≥2)

Parameter	OAC + AP (N = 2735)	OAC Alone (17,892)
Sex male, n (%)	1630 (59.6%)	8392 (46.9%)
Age in years, mean (SD)	71.3 (9.7)	72.6 (9.5)
BMI, kg/m^2, mean (SD)	28.1 (5.6)	27.9 (5.8)
SBP/DBP, mean	133.6/79.8	135.5/80.5
Pulse rate, bpm, mean (SD)	90.9 (26.8)	91.3 (26.6)
LVEF %, mean (SD)	52.6 (13.9)	56.6 (12.5)
CHA_2DS_2-VASc, median	4	3
Medical history, n (%)		
CHF	745 (27.2%)	3405 (19.0%)
CAD	1123 (41.1%)	1960 (11.0%)
ACS	650 (23.8%)	877 (4.9%)
Carotid occlusive disease	142 (5.2%)	404 (2.3%)
DVT/PE	101 (3.7%)	541 (3.0%)
CABG	207 (7.6%)	231 (1.3%)
Stroke/TIA	493 (18.0%)	1832 (10.2%)
Bleeding	80 (2.9%)	307 (1.7%)
Hypertension	2342 (85.6%)	14,761 (82.5%)
Hypercholesterolemia	1,407 (51.4%)	6848 (38.3%)
DM, type 1 or 2	905 (33.1%)	4137 (23.1%)
CKD, moderate–severe	395 (14.4%)	1989 (11.1%)

© 2020 Fox KAA et al. *JAMA Network Open.*

ACS, acute coronary syndrome; CABG, coronary artery bypass graft; CAD, coronary artery disease; CHF, congestive heart failure; CKD, chronic kidney disease; DBP, diastolic blood pressure; DM, diabetes mellitus; DVT, deep vein thrombosis; LVEF, left ventricular ejection fraction; PE, pulmonary embolism; SBP, systolic blood pressure; TIA, transient ischemic attack.

© 2020 Fox KAA et al. *JAMA Network Open.*

eTable 3. Falsification Analysis—Non-CV Mortality Calculated over 12-month Follow-up

	OAC + AP			OAC Alone			Crude HR (95% CI)	aHR (95% CI)
	**Events**	**Subjects**	**Risk/1000 Pts**	**Events**	**Subjects**	**Risk/1000 Pts**		
ITT (multivariate)	24	2541	945	200	17,673	11.32	0.85 (0.55–1.29)	0.76 (0.48–1.22)
ITT (PS matched)	23	2380	966	28	2380	11.76		0.83 (0.48–1.44)

ITT, intent-to-treat analysis with variable follow-up; PS, propensity score.

© 2020 Fox KAA et al. *JAMA Network Open.*

eFigure 1. Relative Risk (Hazard Ratios, Unadjusted and Adjusted[1]) for Study Outcomes in High-Risk AF Patients (CHA_2DS_2-Vasc Score ≥2) Treated with OAC Plus AP or OAC Alone (Reference) over 12 Months (Intent-to-Treat Analyses).

[1]Adjusted for 40 covariates as shown in Supplementary material, Table S1.

	OAC+ AP*	OAC*	aHR (95%CI)
All-cause mortality	119	534	1.21 (0.97-1.51)
MI/ACS	25	75	1.14 (0.68-1.92)
Stroke	39	149	1.55 (1.04-2.3)
Stroke and/or systemic embolism	40	169	1.37 (0.93-2.01)
Any bleeding events	161	689	1.42 (1.17-1.72)
Major bleeding event	30	129	1.43 (0.91-2.23)
Major bleeding event (including hemorrhagic stroke)	34	139	1.46 (0.96-2.22)
Major and NMCR bleeding	74	312	1.5 (1.13-1.99)

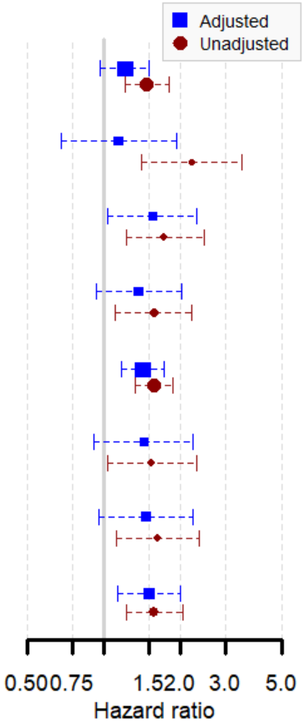

*Number of events

© 2020 Fox KAA et al. *JAMA Network Open.*

eFigure 2. Relative Risk (Hazard Ratios, Unadjusted and Adjusted[1]) for Study Outcomes in High-Risk AF Patients (CHA_2DS_2-Vasc Score ≥2) Treated with OAC Plus AP or OAC Alone (Reference) over 3 Months (Intent-to-Treat Analyses).

[1]Adjusted for 40 covariates as shown in Supplementary material, Table S1.

	OAC+ AP*	OAC*	aHR (95%CI)
All-cause mortality	36	150	1.25 (0.82-1.89)
MI/ACS	13	25	1.74 (0.78-3.91)
Stroke	13	49	1.68 (0.85-3.32)
Stroke and/or systemic embolism	13	51	1.61 (0.82-3.17)
Any bleeding events	70	254	1.58 (1.17-2.14)
Major bleeding event	13	41	1.94 (0.94-4.01)
Major bleeding event (including hemorrhagic stroke)	13	46	1.77 (0.87-3.59)
Major and NMCR bleeding	33	110	1.94 (1.24-3.03)

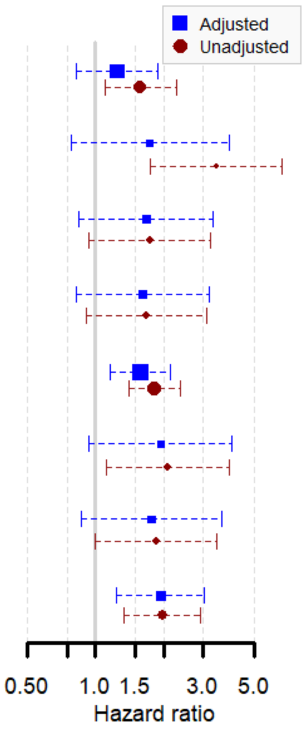

** Number of events*

© 2020 Fox KAA et al. *JAMA Network Open.*

eAppendix. Group Information

The GARFIELD-AF Registry Investigators include the following:

Global Steering Committee: University College London, London, United Kingdom: Ajay K. Kakkar (chair); University of Besançon, Besançon, France: Jean-Pierre Bassand; St. George's University of London, London, United Kingdom: A. John Camm; University of Warwick, Coventry, United Kingdom: David A. Fitzmaurice; Harvard Medical School, Brigham and Women's Hospital, Boston, Massachusetts: Samuel Z. Goldhaber; Tokai University School of Medicine, Kanagawa, Japan: Shinya Goto; Formerly Haemostasis and Thrombosis Research Group, Institute for Experimental Oncology and Therapy Research, Technical University of Munich, Munich, Germany: Sylvia Haas; University Hospital of Heidelberg, Heidelberg, Germany: Werner Hacke; University of Milano-Bicocca, Milan, Italy: Lorenzo G. Mantovani; Bayer AG, Berlin, Germany: Frank Misselwitz; Duke Clinical Research Institute, Durham, North Carolina: Karen S. Pieper; University of Edinburgh, Edinburgh, United Kingdom: Kieth A. A. Fox; McMaster University, Hamilton, Ontario, Canada: Alexander G. G. Turpie; Bayer AG, Berlin, Germany: Martin van Eickels; Onze Lieve Vrouwe Gasthuis, Amsterdam, the Netherlands: Freek W. A. Verheugt.

Publications Committee: St. George's University of London, London, United Kingdom: A. John Camm, Chair; University of Besançon, Besançon, France: Jean-Pierre Bassand; Harvard Medical School, Brigham and Women's Hospital, Boston, Massachusetts: Samuel Z. Goldhaber; Formerly Haemostasis and Thrombosis Research Group, Institute for Experimental Oncology and Therapy Research, Technical University of Munich, Munich, Germany: Sylvia Haas; University of Edinburgh, Edinburgh, United Kingdom: Kieth A. A. Fox; Thrombosis Research Institute, United Kingdom: Gloria Kayani; University of Milano-Bicocca, Milan, Italy: Lorenzo G. Mantovani.

Audit Committee: University of Edinburgh, Edinburgh, United Kingdom: Keith A. A. Fox; Mayo Clinic, Rochester, Minnesota: Bernard J. Gersh.

GARFIELD-AF National Coordinators: Universidad Nacional de Tucumán, San Miguel de Tucuman, Tucuman, Argentina: Hector Lucas Luciardi; The Alfred Hospital, Melbourne, Victoria, Australia: Harry Gibbs; Medizinische Universität Graz Klinische Abteilung für Angiologie, Graz, Austria: Marianne Brodmann; AZ KLINA, Cardiology, Brasschaat, Belgium: Frank Cools; Hospital das Clínicas da Faculdade de Medicina da USP, Sao Paulo, Sao Paulo, Brazil: Antonio Carlos Pereira Barretto; McMaster University, Hamilton, Ontario, Canada: Stuart J. Connolly; McMaster University, Hamilton, Ontario, Canada: John Eikelboom; Pontificia, Universidad Catolica, Santiago, Chile: Ramon Corbalan; National Center for Cardiovascular Disease, Chinese Academy of Medical Sciences and Peking Union Medical College, Beijing, China: Zhi-Cheng Jing; University Hospital Motol, Prague, Czech Republic: Petr Jansky; Bispebjerg and Frederiksberg Hospital, Denmark: Jørn Dalsgaard Nielsen; National Heart Institute, Cairo, Egypt: Hany Ragy; Tampere University Hospital, Tampere, Finland: Pekka Raatikainen; Georges Pompidou Hospital, Paris, France: Jean-Yves Le Heuzey; Vivantes Neukoelln Medical Center, Berlin, Germany:

Harald Darius; Hungarian Institute of Cardiology, Budapest, Hungary: Matyas Keltai; Sir Ganga Ram Hospital, Delhi, India: Jitendra Pal Singh Sawhney; University of Perugia School of Medicine, Perugia, Italy: Giancarlo Agnelli; University of Perugia School of Medicine, Perugia, Italy: Giuseppe Ambrosio; Osaka National Hospital, Osaka, Japan: Yukihiro Koretsune; Instituto de Cardiologia y Medicina Vascular, TecSalud, Tecnológico de Monterrey, Monterrey, Mexico: Carlos Jerjes Sánchez Díaz; Institute Maastricht, Maastricht University Medical Center, Maastricht, the Netherlands: Hugo Ten Cate; Oslo University Hospital, Oslo, Norway: Dan Atar; Institute of Cardiology, Warsaw, Poland: Janina Stepinska; Atherothrombosis Clinical Problems Laboratory, Cardiology Research and Production Center, Moscow, Russia: Elizaveta Panchenko; National University Heart Centre, Singapore: Toon Wei Lim; University of the Witwatersrand, Johannesburg, South Africa: Barry Jacobson; University College of Medicine, Seoul, South Korea: Seil Oh; Hospital de la Santa Creu i Sant Pau, Barcelona, Spain: Xavier Viñolas; Karolinska Institutet, Stockholm, Sweden: Marten Rosenqvist; University Hospital Zurich, Zurich, Switzerland: Jan Steffel; Ramathibodi Hospital, Mahidol University, Bangkok, Thailand: Pantep Angchaisuksiri; Hacettepe University Faculty of Medicine, Ankara, Turkey: Ali Oto; National Scientific Center, MD Strazhesko Institute of Cardiology, Kiev, Ukraine: Alex Parkhomenko; Heart and Vascular Institute, Cleveland Clinic, Abu Dhabi, United Arab Emirates: Wael Al Mahmeed; University of Warwick, Coventry, United Kingdom: David Fitzmaurice; Harvard Medical School, Brigham and Women's Hospital, Boston, Massachusetts: Samuel Z. Goldhaber.

GARFIELD-AF National Investigators:

China: Peking University People's Hospital, Beijing: Dayi Hu, Yihong Sun, Lei Li, Wenling Liu and Yuanfeng Gao; Southwest Hospital, Third Military Medical University, Chongqing, China: Kangning Chen and Hong Zhai; Beijing 301 Hospital, Beijing, China: Yusheng Zhao and Ran Zhang; The First Affiliated Hospital of Wenzhou Medical College, Wenzhou, China: Huaiqin Zhang and Xiao Chen; The First Affiliated Hospital, School of Medicine, Zhejiang University, Hangzhou, China: Jiyan Chen, Tingting Liu, Kan Wang, and Xiaosheng Hu; Nanfang Hospital of Southern Medical University, Guangzhou, China: Shiping Cao, Qiong Zhan, Jingshan Yang, and Xingfu Huang; Union Hospital of Tongji Medical College of Huazhong Science and Technology University, Wuhan, China: Daowen Wang¸ Ting Yu, Xiaoxue Yan and Yang Bai; Fuwai Hospital, Beijing, China: Yuejin Yang, Xuesi Wu Qian Zhang, Zhicheng Jing, Xiaoliang Luo, Zhicheng Jing and Xiaojin Gao; The First Affiliated Hospital of Xiamen University, Xiamen, China: Weihua Li and Wuyang Zheng; Daqing Oilfield General Hospital, Daqing, China: Hui Li, Yanhong Li, Hongying Yu, Tiebing Song and Shanshan Feng; The Second Affiliated Hospital of Chongqing Medical University, Chongqing, China: Yuehui Yin, Xianbin Lan, and Bei Zhou; The First Affiliated Hospital of Liaoning Medical University, Jinzhou, China: Guizhou Tao, Ge Tian and Xiaojing Shi; China-Japan Union Hospital of Jilin University, Changchun, China: Ping Yang and Bing Li; Renji Hospital Shanghai Jiaotong University School of Medicine, Shanghai, China: Yingmin Chen; Northern Jiangsu People's Hospital, Yangzhou, China: Shenghu He and Yi Zhang; China-Japan Friendship Hospital, Beijing, China: Yong Wang and Jing Li; Sir Run Run Shaw

Hospital, Hangzhou, China: Guosheng Fu, Yang Ye, and Xia Sheng; Jinzhou Central Hospital, Jinzhou, China: Xin Li; Guangzhou Red Cross Hospital, Guangzhou, China: Tongguo Wu, Jin Chen, Cancan Zuo and Xiaobi Guo; The Second Affiliated Hospital of Nanchang University, Nanchang, China: Xiaoshu Cheng, Qinmei Xiong, Aijuan Xu, Juxiang Li, and Jianhua Yu; Peking Union Medical College Hospital, Beijing, China: Xiaowei Yan, Ruiyi Xu and Xue Lin; Baotou Central Hospital, Baotou, China: Ruiping Zhao, Yangyang Liu, and Rina Wu; Haikou City People's Hospital, Haikou, China: Moshui Chen, Tianyi Ma, Yixue Zhang, Zhihong Zhou, and Xiaoai He; The Second Xiangya Hospital of Central South University, Changsha, China: Longgen Xiong, Li Wang, Chuanfang Cheng, Chengfeng Luo, and Zhicheng Lu; Shantou Central Hospital, Shantou, China: Ping Chen and Ying Wang; The Second Affiliated Hospital of Soochow University, Suzhou, China: Yang Jiao and Xinyi Zhu; Hunan Province People's Hospital, Changsha, China: Ying Guo, Qinghua Fu, Qin Yang, Na Li, Jianqiang Peng, and Qiong Xie; General Hospital of Ningxia Medical University, Yinchuan, China: Li Xue, Weina Guo, Ruhua He, Lin Chen, and Guoshan Zhang; Second Affiliated Hospital of Shanxi University, Taiyuan, China: Zhiming Yang, Fengzhi Wang, Shuai Feng, Lin Yang, Jinxiu Zhang, and Chunlin Bai.

India: Sujata Birla Hospital & Medical Research Centre, Nashik, India: Praveen Jadhavm, Priyanka Dhakrao, and Deepak Ghumare; Lalitha Super Speciality Hospital, Guntur, India: Raghava Sarma and Naga Malleswara Rao; Shree Multispecialty Hospital, Pune, India: Govind Kulkarni, Unnati Joshi, Bhakti Deshpande, Shankar G Panse, Anjali Sable, and Amol Lawande; Heart and General Hospital, Jaipur, India: Prakash Chandwani and Mahendra Sharma; Shree Krishna Hospital & Heart Care Centre, Ahmedabad, India: Rasesh Atulbhai Pothiwala, Dhrumi Shah, Nehal Sadhu, and Pratik Dwivedi; West Fort Hi-Tech Hospital Pvt Ltd, Thrissur, India: Mohanan Padinhare Purayil, Divin Davies, and Manoj Earath; Sterling Hospital, Ahmedabad, India: Shrenik Shah; Sterling Hospital, Vadodara, India: Kamaldeep Chawla, Amish Ganatra, and Khyati Patel; K.L.E. Society's Dr Prabhakar Kore Hospital and Medical Research Centre, Belgaum, India: Veerappa Annasaheb Kothiwale, James Kot, Kinjal Shah, and Vineeta Dhyani; Chinmaya Narayana Institute of Neuroscience, Bangalore, India: Bagirath Raghuraman, Sudheer Garapati, Ayyappa N, and Subramani Krishnappa; Vijan Cardiac and Critical Care, Nashik, India: Vinod Madan Vijan and Mahesh Dargude; Sir Gangaram Hospital, New Delhi, India: Jitendra Sawhney and Somya Duhan; St John's Medical College Hospital, Bangalore, India: Ganapathi Bantwal and Sudha Suresh; Crescent Hospital & Heart Centre, Nagpur, India: Aziz Khan, Puja Sherke, Satish Giradkar, Kajal Gaikwad, Reshma Kohade, and Alka Gedam; S. R. Kalla Memorial Gastro & General Hospital, Jaipur, India: Ramdhan Meena, Jugal Gupta, Vipin Jain, and Shweta Paliwal; Chopda Medicare & Research Centre, Nashik, India: Manojkumar Chopada, Bhushan Suryawanshi, Priyanka More, and Anand Rokade; Chinmaya Narayana Super Speciality Hospital, Bangalore, India: Sunitha Abraham, Rajendran Karthikeyan, and Anusha Kotha; Bisne's Heart Institute, Nagpur, India: Vikas Bisne, Bhowari Jamunkar, Neha Madarkar, Darshana Nandekar, and Ruchieta Kishty; Kerala Institute of Medical Sciences, Trivandrum, India: Govindan Vijayaraghavan and Kavitha VC; Rabindranath Tagore International Institute of Cardiac Sciences (RTIICS), Vishakhapatnam, India: Debabrata Roy, Partha Bhattacharjee, and Anjuli Barai; Sri Venkateswara Institute of Medical

© 2020 Fox KAA et al. *JAMA Network Open.*

Sciences, Tirupati, India: Rajashekhar Durgaprasad, Latheef Kasala, and Vanaja Vanajakshamma; Mysore Medical College and Research Institute, Mysore, India: A.G. Ravi Shankar, Rahul ER, Kanuri Prashanth, Prasad Shiva, and Dilip Teja Naidu; Apollo BGS Hospital, Mysore, India: Sunil Kumar, Dilipa Naik, and Manasa Rao; Dayanand Medical College and Hospital, Ludhiana, India: Dinesh Jain, Sukhjeet Kaur, Anju Jain, and Aarushi Jain; Medanta The Medicity, Gurgaon, India: Kartikeya Bhargava, Dhriti Adak, Krishan Sharma, and Kuldeep Chauhan; Vikram Hospital & Heart Care, Mysore, India: Vinay Kumar, Devina Igoor, Manjappa M, Vinushree K, Rathna RL, Haseena Begum, Vaibhavi PS, and Bharath Kumar; M. S. Ramaiah Medical College and Hospital, Bangalore, India: Udigala Madappa Nagamalesh, Dayakar P, Sanjana Xavier, Swathi Balaraju, Vijay Kumar, and Beena Lokesh; Indraprastha Apollo Hospitals, Delhi, India: Rajeeve Kumar Rajput, Swati Sharma, Huma Yusuf, Arti Malik, and Swati Singhal.

Japan: NHO Osaka National Hospital, Osaka-shi, Japan: Yukihiro Koretsune; Kanamorikai Kanamori Clinic, Uto-shi, Japan: Seishu Kanamori; Keiaikai Saga Memorial Hospital, Saga-shi, Japan: Kenichi Yamamoto; Kohokai Fukuoka Sanno Hospital, Fukuoka-shi, Japan: Koichiro Kumagai; Fukuoka City Medical Association Hospital, Kitakyushu-shi, Japan: Yosuke Katsuda; Sagaken Medical Center Koseikan, Saga-shi, Japan: Keiki Yoshida and Kemji Sadamatsu; Fukuokairyodan Chidoribashi Hospital, Fukuoka-shi, Japan: Fumitoshi Toyota; Juryokai Kumamoto Kino Hospital, Kumamoto-shi, Japan: Yuji Mizuno; NHO Kumamoto Saishunso National Hospital, Koshi-shi, Japan: Ikuo Misumi; Chiyukai Fukuoka Wajiro Hospital, Fukuoka-shi, Japan: Hiroo Noguchi; Fukuokaken Saiseikai Futsukaichi Hospital, Chikushino-shi, Japan: Shinichi Ando; Yukeikai Suetsugu Naika Junkankika, Kanoya-shi, Japan: Tetsuro Suetsugu; JCHO Kanazawa Hospital, Kanazawa-shi, Japan: Masahiro Minamoto; Public Central Hospital of Matto Ishikawa, Hakusan-shi, Japan: Hiroyuki Oda; Shuwakai Shuwa General Hospital, Kasukabe-shi, Japan: Susumu Adachi; Hoyukai Chitose Hoyukai Hospital, Chitose-shi, Japan: Kei Chiba; Yuaikai Oda Regional Medical Center, Kashima-shi, Japan: Hiroaki Norita; Tsuruta Naika Junkankika Clinic, Saga-shi, Japan: Makoto Tsuruta; Koyanagi Naika Junkankika Clinic, Tosu-shi, Japan: Takeshi Koyanagi; Yamamoto Heart Clinic, Kurume-shi, Japan: Kunihiko Yamamoto; Ando Naika Junkankika Clinic, Fukuoka-shi, Japan: Hiroshi Ando; Ryubokai Higashi Diabetes and Cardiovascular Clinic, Tamana-shi, Japan: Takayuki Higashi; Seikeikai Okada Hospital, Nagato-shi, Japan: Megumi Okada; Kenshinkai Azakami Naika Junkankika Clinic, Nogata-shi, Japan: Shiro Azakami; Kokoronohi Komaki Clinic, Aira-shi, Japan: Shinichiro Komaki; Eikokai Kumeda Naika Junkankinaika Clinic, Kagoshima-shi, Japan: Kenshi Kumeda; Murayama Naika Junkankika Clinic, Kagoshima-shi, Japan: Takashi Murayama; Shunseikai Higuchi Hospital, Kasuga-shi, Japan: Jun Matsumura; KKR Chihaya Hospital, Fukuoka-shi, Japan: Yurika Oba; Chishinkai Nishimura Naika Noshinkeigeka Hospital, Kumamoto-shi, Japan: Ryuji Sonoda; Hibikikai Goto Naika Clinic, Kumamoto-shi, Japan: Kazuo Goto; Minoda Naika Junkankika, Hitoyoshi-shi, Japan: Kotaro Minoda; Haraguchikai Haraguchi Junkankika Naika Clinic, Kumamoto-shi, Japan: Yoshikuni Haraguchi; Toeikai Suefuji Naika Junkankika, Kumamoto-shi, Japan: Hisakazu Suefuji; Miyagi Junkanki Naika, Yatsushiro-shi, Japan: Hiroo Miyagi; Jinseikai Kato Clinic, Osaka-shi, Japan: Hitoshi Kato;

© 2020 Fox KAA et al. *JAMA Network Open.*

Nakamura Medical and Circuratory Clinic, Kitakyushu-shi, Japan: Tsugihiro Nakamura; Nakamura Tadashi Naika Junkankika Clinic, Kitakyushu-shi, Japan: Tadashi Nakamura; Nandate Naika Junkankika, Kitakyushu-shi, Japan: Hidekazu Nandate; Zaitsu Cardiovascular Clinic, Fukuoka-shi, Japan: Ryuji Zaitsu; Fukuoka Teishin Hospital, Fukuoka-shi, Japan: Yoshihisa Fujiura; Hyodokai Yoshimura Naika, Nagato-shi, Japan: Akira Yoshimura; Numata Naika Kokyukika, Fujisawa-shi, Japan: Hiroyuki Numata; Handa Naika Clinic, Kanazawa-shi, Japan: Jun Ogawa; Tatematsu Clinic, Nagoya-shi, Japan: Hiroshi Tatematsu; Taikai Clinic, Satsumasendai-shi, Japan: Yasuyuki Kamogawa; Murakami Kinshiro Cardiovascular Internal Clinic, Sasebo-shi, Japan: Kinshiro Murakami; Wakasa Medical Clinic, Kanazawa-shi, Japan: Yutaka Wakasa; Yamasawa Naika, Koriyama-shi, Japan: Masanori Yamasawa; Maekawa Medical Clinic, Yokohama-shi, Japan: Hiromitsu Maekawa; Kokankai Kokan Clinic, Kawasaki-shi, Japan: Sumihisa Abe; Kikokai Kihara Cardiovascular Internal Medicine Clinic, Asahikawa-shi, Japan: Hajime Kihara; Close To You Est Clinic, Hirosaki-shi, Japan: Satoru Tsunoda; Seiwakai Nishiarai Heart Center Hospital, Adachi-ku, Japan: Katsumi Saito; Tachibana-Iiyama Hospital, Kagoshima-shi, Japan: Hiroki Tachibana; Showakai Imakiire General Hospital, Kagoshima-shi, Japan: Ichiro Oba; Yushikai Kuwahata Clinic, Kagoshima-shi, Japan: Takashi Kuwahata; Hakuaikai Makiminato Central Hospital, Urasoe-shi, Japan: Satoshi Higa; Doshinkai Gushiken Junkanki Naika, Urasoe-shi, Japan: Masamichi Gushiken; Eto Clinic, Nichinan-shi, Japan: Takuma Eto; Shonankai Shonan Hospital, Okinawa-shi, Japan: Hidetoshi Chibana; Fujisawa Clinic, Itoshima-shi, Japan: Kazuaki Fujisawa; Seishinkai Inoue Hospital, Itoshima-shi, Japan: Yuhei Shiga; Sumi Junkankinaika Clinic, Oita-shi, Japan: Hirokuni Sumi; Chojukai Nagatomo Naika Junkankinaika Clinic, Kitakyushu-shi, Japan: Toshihisa Nagatomo; Tenyokai Chuo Clinic, Kagoshima-shi, Japan: Yoshihiko Atsuchi; Nagoshi Naika, Miyazaki-shi, Japan: Toshiro Nagoshi; Ojukai Sanno Naika, Aira-shi, Japan: Kazuhisa Sanno; Karinkai Murakamikarindoh Hospital, Fukuoka-shi, Japan: Fumihiro Hoshino; Yokota Naika, Miyazaki-shi, Japan: Naoto Yokota; Kanoya Medical Center Citizens' Health Plaza, Kanoya-shi, Japan: Masahiro Kameko; Tabuchi Naika Junkankika, Kikuchi-gun, Japan: Toshifumi Tabuchi; Ishizawa Junkankinaika, Ebino-shi, Japan: Munesumi Ishizawa; Hojunkai Fujiura Junkankinaika Clinic, Nichinan-shi, Japan: Yoshitake Fujiura; Seijinkai Ikeda Hospital, Kanoya-shi, Japan: Daisuke Ikeda; Seto Junkankinaika Clinic, Fukuoka-shi, Japan: Taku Seto; Oita Red Cross Hospital, Oita-shi, Japan: Tetsu Iwao; Kieikai Fukuoka Kieikai Hospital, Fukuoka-shi, Japan: Norio Ishioka and Kohei Nii; Omotokai Ohama Daiichi Hospital, Naha-shi, Japan: Koichi Oshiro; Tsuchida Clinic of Internal and Cardiovascular Medicine, Nagaoka-shi, Japan: Keizo Tsuchida; Hatori Clinic, Kawasaki-shi, Japan: Yutaka Hatori; Takeuchi Clinic, Kobe-shi, Japan: Motoshi Takeuchi; Takezawa Clinic (Nagoya), Nagoya-shi, Japan: Hiroto Takezawa; Jiaikai Imamura General Hospital, Kagoshima-shi, Japan: Shinjiro Nagano; Matsushirokai Hayato Spa Hospital, Kirishima-shi, Japan: Masaaki Iwaki; Nakamura Cardiovascular Clinic, Itoshima-shi, Japan: Yuichiro Nakamura; Sanyukai Saino Clinic, Tokorozawa-shi, Japan: Naomasa Miyamoto; Taguchi Junkankikanaika Clinic, Miyakonojo-shi, Japan: Toshifumi Taguchi; Ashida Medical Clinic, Tamba-shi, Japan: Ko Ashida; Yoshizawa Heart Clinic, Setagaya-ku, Japan: Naoto Yoshizawa; Agata Clinic, Obihiro-shi, Japan: Jun Agata; Matsukawa Heart Clinic,

Edogawa-ku, Japan: Seishiro Matsukawa; Yuaikai Tomishiro Chuo Hospital, Tomigusuku-shi, Japan: Osamu Arasaki and Tetsuji Shinjo; Fukuoka Clinic, Nichinan-shi, Japan: Shuji Fukuoka; South Tokyo Heart Clinic, Machida-shi, Japan: Hirofumi Murakami; Mishima Naika Clinic, Kitamorokata-gun, Japan: Kazuya Mishima; Naha City Hospital, Naha-shi, Japan: Mamoru Manita; Kenshinkai Minamino Cardiovascular Hospital, Hachioji-shi, Japan: Yoshiki Hata; Caress Sapporo Hokko Memorial Clinic, Sapporo-shi, Japan: Ichiro Sakuma; Tokyobay Urayasu Ichikawa Medical Center, Urayasu-shi, Japan: Kotaro Obunai; Takamura Naika Iin, Otaru-shi, Japan: Ichiro Takamura; Akutsu Naika Iin, Otaru-shi, Japan: Mitsuyuki Akutsu; Unoki Junkankinaika Iin, Miyakonojo-shi, Japan: Toshihide Unoki; Kochikukai Go Neurosurgical Clinic, Chikushi-gun, Japan: Yoshinori Go; Shinseikai Ikemura Internal Medicine Clinic, Miyakojima-shi, Japan: Makoto Ikemura; Miike Junkankika Naika Iin, Kusu-gun, Japan: Shoji Morii; Tokachi Heart Clinic, Kato-gun, Japan: Shigeru Marusaki; Doi Naika Junkankika, Miyazaki-shi, Japan: Hideo Doi; Tanaka Junkankinaika Clinic, Kobayashi-shi, Japan: Mitsuru Tanaka; Kotokukai Kusumoto Naika Iin, Izumi-shi, Japan: Takaaki Kusumoto; Otaru Kyokai Hospital, Otaru-shi, Japan: Shigeo Kakinoki; Ogurusu Heart Clinic, Kasaoka-shi, Japan: Chiga Ogurusu; Wayokai Murata Clinic, Sanyoonoda-shi, Japan: Kazuya Murata; Shimoyama Clinic, Yonago-shi, Japan: Masaki Shimoyama; Jisshokai Nakatsuka Naika, Hofu-shi, Japan: Masami Nakatsuka; Asakurashinryojo Asakura Clinic, Imabari-shi, Japan: Yutaka Kitami; Soyokaze Cardiovascular Medicine and Diabetes Care, Matsuyama-shi, Japan: Yoichi Nakamura; Shogen Naika Clinic, Sendai-shi, Japan: Hiroshi Oda; Tojukai Ito Naika Iin, Shimonoseki-shi, Japan: Rikimaru Oyama; Ageta Naika Clinic, Nichinan-shi, Japan: Masato Ageta; Mita Medical-Cardiovascular Clinic, Sapporo-shi, Japan: Teruaki Mita; Nagao Naika Junkanki Clinic, Sapporo-shi, Japan: Kazuhiko Nagao; Mito Naika Junkanki Clinic, Sapporo-shi, Japan: Takafumi Mito; Saito Naika Clinic, Hakodate-shi, Japan: Tsutomu Saito; Juntenkai Teshima Clinic, Kure-shi, Japan: Junichi Minami; Yotsuba Circulation Clinic, Matsuyama-shi, Japan: Mitsunori Abe; Fujii Heart Clinic, Kurashiki-shi, Japan: Masako Fujii; Okawa Naika, Kochi-shi, Japan: Makoto Okawa; Kawaguchi Heart Clinic, Kawaguchi-shi, Japan: Tsuneo Fujito; Aishin Clinic, Yachiyo-shi, Japan: Toshiya Taniguchi; Gyokushinkai Kashima Heart Clinic, Kamisu-shi, Japan: Tenei Ko; Hakushinkai Hiro Clinic, Takasaki-shi, Japan: Hiroshi Kubo; Chiba Heart Clinic, Chiba-shi, Japan: Mizuho Imamaki; Heart Clinic, Tatebayashi-shi, Japan: Masahiro Akiyama; Ueda Heart Clinic, Tatsuno-shi, Japan: Takashi Ueda; Dobashi Naika Iin, Sendai-shi, Japan: Hironori Odakura; Inagaki Clinic, Itami-shi, Japan: Masahiko Inagaki; Katsube Clinic, Nishinomiya-shi, Japan: Yoshiki Katsube; Nakata Naika Clinic, Nishinomiya-shi, Japan: Atsuyuki Nakata; Tojo Clinic, Kato-shi, Japan: Shinobu Tomimoto; Juzenkai Tateyama Junkankinaika Geka, Tateyama-shi, Japan: Mitsuhiro Shibuya; Kaiseikai Nakano Hospital, Shibukawa-shi, Japan: Masayuki Nakano; Ito Cardiovascular Clinic, Saiki-shi, Japan: Kenichiro Ito; Matsuta Heart Clinic, Yamatokoriyama-shi, Japan: Masahiro Matsuta; Yi Yuan Hui Ishiguro Clinic, Gifu-shi, Japan: Motoyuki Ishiguro; Minagawa Clinic, Gifu-shi, Japan: Taro Minagawa; Wada Clinic (Gunma), Tomioka-shi, Japan: Masamichi Wada; Mukawa Heart Clinic, Ogaki-shi, Japan: Hiroaki Mukawa; Mizuguchi Clinic, Suzuka-shi, Japan: Masato Mizuguchi; Kosumosukai Okuda Clinic,

© 2020 Fox KAA et al. *JAMA Network Open.*

Ube-shi, Japan: Fumio Okuda; Meihokai Meiho Clinic, Tsuruga-shi, Japan: Teruaki Kimura; Keiyukai Taga Naika Junkankika Iin, Fukui-shi, Japan: Kuniaki Taga; Techigawara Clinic, Koriyama-shi, Japan: Masaaki Techigawara; Asunarokai Igarashi Clinic, Shirakawa-shi, Japan: Morio Igarashi; HAL Clinic, Niigata-shi, Japan: Hiroshi Watanabe; Seo Naika Clinic, Osaka-shi, Japan: Toshihiko Seo; Hiramitsu Heart Clinic, Nagoya-shi, Japan: Shinya Hiramitsu; Yuaikai Higashi Junkanki Clinic, Toyohashi-shi, Japan: Hiroaki Hosokawa; Hoshiai Clinic, Owariasahi-shi, Japan: Mitsumoto Hoshiai; Hibino Naika Clinic, Toyota-shi, Japan: Michitaka Hibino; Miyagawa Clinic, Nisshin-shi, Japan: Koichi Miyagawa; Heart Naika Clinic, Yatomi-shi, Japan: Hideki Horie; Wakeikai Sugishita Iin, Gujo-shi, Japan: Nobuyoshi Sugishita; Meisei Shiga Clinic, Obu-shi, Japan: Yukio Shiga; Heart Clinic Kokoro, Koka-shi, Japan: Akira Soma; Neya Naika Clinic, Itabashi-ku, Japan: Kazuo Neya; Onga Hospital, Onga-gun, Japan: Tetsuro Yoshida; Akahane Clinic, Nagano-shi, Japan: Kunio Akahane; Shofukai Naito Hospital, Kurume-shi, Japan: Sen Adachi; Takanaka Clinic, Hamamatsu-shi, Japan: Chiei Takanaka; Ueda Neurosurgery, Miyazaki-shi, Japan: Takashi Ueda; Matsui Clinic, Hamamatsu-shi, Japan: Saori Matsui; Heart Clinic Kanda, Okazaki-shi, Japan: Hirofumi Kanda; Kaneko Naika Junkankika Clinic, Hamamatsu-shi, Japan: Masanori Kaneko; Nagasaka Heart Clinic, Hamamatsu-shi, Japan: Shiro Nagasaka; Taguchi Naika Junkankika Iin, Hamamatsu-shi, Japan: Atsushi Taguchi; Nitobe Memorial Nakano General Hospital, Nakano-ku, Japan: Shuta Toru, Kazuyuki Saito, Akiko Miyashita and Hiroki Sasaguri; Osaka General Hospital of West Japan Railway Company, Osaka-shi, Japan: Jin Nariyama, Hiroyuki Miyamoto, Yusuke Nishida, Tatsuya Suga and Hiroki Nagata; Hatsuno Clinic, Iruma-gun, Japan: Taketo Hatsuno; Iwase Internal Medicine Cardiology Clinic, Shibuya-ku, Japan: Takash Iwase; Itsukikai Heart Clinic, Katsushika-ku, Japan: Kazuki Sato; Chikamorikai Chikamori Hospital, Kochi-shi, Japan: Kazuya Kawai; Jinikai Makita General Hospital, Ota-ku, Japan: Tomobumi Kotani; Tsuji Clinic, Toyonaka-shi, Japan: Tsuyoshi Tsuji; Sakai Medical Clinic, Higashiosaka-shi, Japan: Hirosumi Sakai; Nishino Clinic, Kochi-shi, Japan: Kiyoshi Nishino; Tachikawa IM Clinic, Tachikawa-shi, Japan: Kenichi Ikeda; Maeda Naika Iin, Niigata-shi, Japan: Kazuo Maeda; Murao Shinryojo, Neyagawa-shi, Japan: Tomohiro Shinozuka; Inoue Cardiovascular Clinic, Oita-shi, Japan: Takeshi Inoue; Kawakami Clinic, Ota-shi, Japan: Koichi Kawakami; Kitazumi Clinic, Itabashi-ku, Japan: Hiromichi Kitazumi; Takagi Cardiology Clinic, Kyoto-shi, Japan: Tsutomu Takagi; Hamaoka Clinic, Nishinomiya-shi, Japan: Mamoru Hamaoka; Kojima Clinic, Odawara-shi, Japan: Jisho Kojima; Sasaki Heart Clinic, Shizuoka-shi, Japan: Akitoshi Sasaki; Ohashi Cardiovascular & Internal Medicine Clinic, Fukuoka-shi, Japan: Yoshihiro Tsuchiya; Tokyo Tenshi Hospital, Hachioji-shi, Japan: Tetsuo Betsuyaku; Hiratsuka Kyosai Hospital, Hiratsuka-shi, Japan: Koji Higuchi; Honda Clinic, Izumo-shi, Japan: Masaaki Honda; Hasegawa Outpatients Clinic for Cardiovascular Disease, Takamatsu-shi, Japan: Koichi Hasegawa; Baba Clinic, Nishitokyo-shi, Japan: Takao Baba; Mineoi Clinic, Matsuyama-shi, Japan: Kazuaki Mineoi; Saien Cardiovascular Medicine Clinic, Morioka-shi, Japan: Toshihiko Koeda; Hirasawa Cardiovascular Medicine Clinic, Asahikawa-shi, Japan: Kunihiko Hirasawa; Sunomata Clinic, Ogaki-shi, Japan: Toshihide Kumazaki; Nakagomi Medical Clinic, Akita-shi, Japan: Akira Nakagomi; Otaki Cardiology Clinic, Bunkyo-ku, Japan: Eiji Otaki; Shindo Clinic,

© 2020 Fox KAA et al. *JAMA Network Open.*

Amagasaki-shi, Japan: Takashi Shindo; Chiyo Clinic, Kitakyushu-shi, Japan: Hiroyoshi Hirayama; Toyoda Heart Clinic, Iwata-shi, Japan: Chikako Sugimoto; Yamagishi Medical Clinic, Yamaguchi-shi, Japan: Takashi Yamagishi; Orange Clinic, Yokohama-shi, Japan: Ichiro Mizuguchi; Nico Nico Heart Clinic, Higashimatsuyama-shi, Japan: Kazunori Sezaki; Niwa Medical Clinic, Kyoto-shi, Japan: Isamu Niwa; Takenaka Clinic, Kyoto-shi, Japan: Ken Takenaka; Nakatani Clinic, Yao-shi, Japan: Osamu Iiji; Taya Clinic, Tsuchiura-shi, Japan: Koichi Taya; Tachikawa General Hospital, Nagaoka-shi, Japan: Hitoshi Kitazawa; Okinawa Tokushukai Chiba Tokushukai Hospital, Funabashi-shi, Japan: Osamu Ueda; Kakuda Clinic, Kahoku-shi, Japan: Hirokazu Kakuda; Ono Medical Clinic, Koto-ku, Japan: Takuya Ono; Oriso Internal Medicine Cardiology Clinic, Ninohe-shi, Japan: Seizo Oriso; Odori Kamata Medicine Clinic, Morioka-shi, Japan: Junya Kamata; Gakuen Heart Clinic, Machida-shi, Japan: Toshihiko Nanke; Miyanomori Memorial Hospital, Sapporo-shi, Japan: Itaru Maeda and Takashi Kawamoto; Sakaemachi Clinic, Tottori-shi, Japan: Yoshifusa Matsuura; JR Hiroshima Hospital, Hiroshima-shi, Japan: Hiroki Teragawa, Yuichi Fujii, Shuichi Nomura and Tomohiro Ueda; Kosekai Iwatsuki-minami Hospital, Saitama-shi, Japan: Yasuyuki Maruyama; Takei Medical Clinic, Odawara-shi, Japan: Kazuo Takei; Horie Clinic, Omihachiman-shi, Japan: Hajime Horie; Clinic Kakehashi, Nagoya-shi, Japan: Tetsutaro Kito; Asuka Clinic, Nakano-ku, Japan: Hiroshi Asano; Matsushita Medical Clinic, Toyonaka-shi, Japan: Koji Matsushita; Nakamura Medical Clinic, Suita-shi, Japan: Masaichi Nakamura; Washizuka Clinic, Kamo-shi, Japan: Takashi Washizuka; Yoshida Heart Clinic, Hiroshima-shi, Japan: Tomoki Yoshida; Sawano Clinic, Yokohama-shi, Japan: Masato Sawano and Koji Matsushita; Arima Shinichi Clinic, Kagoshima-shi, Japan: Shinichi Arima; Kanoya Heart Center, Kanoya-shi, Japan: Hidekazu Arai; Iwamoto Medical Clinic, Zentsuji-shi, Japan: Hisanori Shinohara; Takai Clinic, Kawachinagano-shi, Japan: Hiroyuki Takai; Furukawa Medical Clinic, Nakatsu-shi, Japan: Nobufusa Furukawa; Ota Shinryojo, Awa-shi, Japan: Akira Ota; Yamamoto Naika, Sakai-shi, Japan: Kentaro Yamamoto; Ebetsu City Hospital, Ebetsu-shi, Japan: Kenji Aoki, Masahiko Abe and Rikiya Shinohe; Yamamoto Clinic (Shiga), Moriyama-shi, Japan: Taku Yamamoto; Kasai Naika Junkanki Clinic, Nerima-ku, Japan: Takeaki Kasai; Suzuki Medical Clinic, Minamiawaji-shi, Japan: Shunji Suzuki; KKR Tohoku Kosai Hospital, Sendai-shi, Japan: Shu Suzuki and Kikuyo Takahashi; Sakakibarakosekai Shinjuku Mitsui Building Clinic, Shinjuku-ku, Japan: Nitaro Shibata; Omori Internal Medicine Cardiology Clinic, Fukushima-shi, Japan: Masayuki Watanabe; St. Luke's International Hospital, Chuo-ku, Japan: Yosuke Nishihata, Yutaro Nishi, Hiroyuki Niinuma, Yasuhiro Yokoyama, Hirotsugu Mitsuhashi, Ryo Nakazato, Takeaki Shirai, Yumi Shiina, Atsushi Mizuno, Toru Adachi, Taku Asano, Ikki Komatsu and Masahiro Yamazoe; Yamaichi Building Medical Clinic, Adachi-ku, Japan: Toru Arino; Okuyama Clinic, Murayama-shi, Japan: Masaki Okuyama; Wakiyama Clinic, Karatsu-shi, Japan: Tetsushi Wakiyama; Juntendo University Hospital, Bunkyo-ku, Japan: Tomoko Kato; Sasagawa Clinic, Shibata-shi, Japan: Yasuo Sasagawa; Shuri Jokamachi Clinic Daiichi, Naha-shi, Japan: Takeshi Tana and Ayano Ishihara; Internal Medicine Cardiology Hayashi Clinic, Asahikawa-shi, Japan: Yoshihito Hayashi; Ueki Hospital, Kumamoto-shi, Japan: Shinichi Hirota; Abe Clinic, Fukushima-shi, Japan: Yukihiko Abe; Hidamari no Mori Clinic, Japan: Yoshihiro Saito; Uchiyama Clinic,

Japan: Hirohide Uchiyama; Takeda Heart Clinic, Japan: Hiroshi Takeda; Sakanoue Family Clinic, Japan: Hiroshi Ono; Hoden Clinic, Japan: Shuichi Tohyo; Clinic Hanazono Naika Iin, Japan: Naoto Hanazono; Miyajima Junkanki Naika, Japan: Seiichi Miyajima; Shimono Clinic, Japan: Hisashi Shimono; Koseikai Kizawa Memorial Hospital, Minokamo-shi, Japan: Takuma Aoyama, Shusaku Miyata, Shigekiyo Takahashi, Takahisa Ido, Makoto Yamaura, Keita Suzuki, Yuto Kumai, Gen Tanabe, Takehiro Yamada and Yuka Kawada; Shozawa Clinic, Japan: Yasunobu Shozawa; Kanetsu Chuo Hospital, Japan: Yawara Niijima; Sekishinkai Kawasaki Saiwai Hospital, Japan: Osamu Murai; Sekishinkai Kawasaki Saiwai Clinic, Japan: Osamu Murai; Teikyo University School of Medicine University Hospital, Mizonokuchi, Japan: Hideko Inaba; Nomura Medical Clinic, Japan: Katsumasa Nomura; Saiseikai Fukuoka General Hospital, Japan: Masatsugu Nozoe; Kenkokan Suzuki Clinic, Japan: Kazuo Suzuki; St. Marianna University School of Medicine Hospital, Japan: Toshiyuki Furukawa; Shiraiwa Medical Clinic, Japan: Toshihiko Shiraiwa; Grace Medical Clinic, Japan: Nobuhisa Ito; Nagai Clinic, Japan: Shunichi Nagai; Sendai Ekihigashi Clinic, Japan: Kiyoharu Sato; Dokkyo Medical University Koshigaya Hospital, Japan: Shiro Nakahara; Risshokoseikai Kosei General Hospital, Japan: Yujin Shimoyama; Kojinkai Tsuruhashi Clinic, Japan: Naoko Ohara; Omotecho Family Clinic, Japan: Teruhiko Kozuka; Okita Internal Medicine Clinic, Japan: Hideaki Okita; Endo Clinic (Miyagi), Japan: Masato Endo; Goto Iin, Japan: Tsutomu Goto; Hirose Clinic, Japan: Makoto Hirose; Harada Naika Clinic, Japan: Emiko Nagata; Kogakai Nakanishi Clinic, Japan: Noriyuki Nakanishi; Mori Clinic (Osaka), Japan: Toshizumi Mori; Seki Iin, Japan: Shuichi Seki; Okamoto Naika Iin, Japan: Katsuhiro Okamoto; Moriai Naika Clinic, Japan: Osamu Moriai; Emura Clinic, Japan: Yoko Emura; Fukuda Clinic (Tondabayashi), Japan: Tsuyoshi Fukuda; Date Clinic, Japan: Haruhiko Date; Meikikai Uchimura Kawakami Naika, Japan: Shuichi Kawakami; Ebino Sentoro Clinic, Japan: Sho Nagai; Ueyama Clinic, Japan: Yuya Ueyama; Fudo Clinic, Osaka-shi, Japan: Tetsuro Fudo; Ichikawa Iin, Japan: Mitsuru Imaizumi; Ogawa Naika Clinic, Japan: Takuo Ogawa; Hakujinkai Take Naika Clinic, Japan: Shunsuke Take; Ikeda Naika Hifuka Clinic, Japan: Hideo Ikeda; Nishioka Family Clinic, Japan: Hiroaki Nishioka; Ebisu Clinic, Japan: Norihiko Sakamoto; Ikeoka Clinic, Japan: Kiyomitsu Ikeoka; Wakaki Clinic, Japan: Nobuo Wakaki; Abe Junkanki Clinic, Japan: Masatake Abe; Kazahaya Clinic, Japan: Junji Doiuchi; Kira Cardiovascular Clinic, Japan: Tetsuya Kira; Tada Naika (Hakodate), Japan: Masato Tada; Eiseikai Tsuzaki Cardiovascular Anesthesiology Clinic, Japan: Ken Tsuzaki; Miura Clinic (Sapporo), Japan: Naoya Miura; Fujisawa Cardio-Vascular Clinic, Japan: Yasuaki Fujisawa; Furumoto Clinic, Japan: Wataru Furumoto; Rumoi Central Clinic, Japan: Susumu Suzuki; Honbetsu Cardiovascular Medicine Clinic, Japan: Akinori Fujisawa; Nakamurakai Nakamura Clinic, Japan: Ryosai Nakamura; Soseikai Komatsu Iin, Japan: Hiroyasu Komatsu; Seiwakai Fujiki Clinic, Japan: Rei Fujiki; Keiwakai Kawano Iin, Japan: Shuichi Kawano; Nishizawa Clinic, Japan: Keijiro Nishizawa; Kato Medical Clinic, Japan: Yoji Kato; Tsurugaoka Azuma Clinic, Japan: Junya Azuma; Koseikai Yotsubashi Clinic, Japan: Kiyoshi Yasui; Amano Naika, Japan: Toshio Amano; Yuwakai Sekine Iin, Japan, Yasuhiro Sekine; Honzawa Medical Center, Japan: Tatsuo Honzawa; Koshibu Clinic, Japan: Yuichiro Koshibu; SYU Cardiovascular Clinic, Japan: Yasuhide Sakamoto; Seta Clinic, Japan: Yukihiro

Seta; Miyaguchi Clinic, Japan: Shingo Miyaguchi; Morishita Naika Iin, Japan: Kojuro Morishita; Yoshikawa Medical Clinic, Japan: Yasuko Samejima; Ishizawa Iin, Japan: Toyoshi Sasaki; Iseki Junkanki Naika, Japan: Fumiko Iseki; Clinica de Kobayashi, Japan: Toshiyuki Kobayashi; Kano Clinic, Japan: Hiroshi Kano; Akiyama Heart Clinic, Japan: Jaeyoung Kim; Hiroshi Yamaguchi Clinic, Japan: Hiroshi Yamaguchi; Takagi Naika Junkankika, Japan: Yoichi Takagi; Galatea Pearce Clinic, Japan: Yoko Onuki Pearce; Medical Clinic Suzuki, Japan: Yasuyuki Suzuki; Fukui Clinic, Japan: Takayuki Fukui; Nakayama Clinic (Kochi), Japan: Toru Nakayama; Kanai Clinic, Japan: Hideaki Kanai; Tenshindo Hetsugi Hospital, Japan: Yoshiyuki Kawano; Souseikai Fukuoka Mirai Hospital, Japan: Tetsuji Ino; Miyoshi Junkanki Clinic, Japan: Hironori Miyoshi; Jonan Koen Clinic, Japan: Yasufumi Miyamoto; Shigekiyo Naika Geka, Japan: Masahito Shigekiyo; Marugame Ono Clinic, Japan: Shimato Ono; Tenshindo Hetsugi Clinic, Japan: Yoshiyuki Kawano; Okamoto Iin, Japan: Yutaka Okamoto; Nijumarukai Ubukata Heart Clinic, Japan: Satoshi Ubukata; Juzenkai Kisarazu Heart Clinic, Japan: Kojiro Kodera; Shinseikai Oriuchi Clinic, Japan: Tatsuo Oriuchi; Sanikai Tsurukawa Kinen Hospital, Japan: Naoki Matsumoto; Inagaki Heart Clinic, Japan: Koichi Inagaki; Iseki Clinic, Japan: Atsushi Iseki; Yoshida Clinic Internal & Cardiology, Japan: Tomohiro Yoshida; Aishinkan Aishin Circulatory Organ Clinic, Japan: Toshihiro Goda; Katsuki Naika Clinic, Japan: Tsukasa Katsuki; Sato Naika Shonika Toriage Iin, Japan: Atsushi Sato; Chihaya Heart Clinic, Japan: Etsuo Mori; Tsubokura Clinic, Japan: Toshio Tsubokura; Hiro Clinic, Japan: Hiroshi Shudo; Hakuyokai Fujimoto Clinic, Japan: Shunichi Fujimoto; Katsuya Clinic, Japan: Tomohiro Katsuya; Furukawa Naika Clinic, Japan: Yoshiyuki Furukawa; Ichibangai Sogo Clinic, Japan: Hiroshi Hosokawa; Narumiyakai Narumiya Heart Clinic, Japan: Jun Narumi; Shozankai Yamamoto Naika Junkankika Iin, Japan: Kiichiro Yamamoto; Owari Cardiovascular & Diabetes Clinic, Japan: Masaki Owari; Keiaikai Inakura Clinic, Japan: Takuya Inakura; Anno Clinic, Japan: Takafumi Anno; Shirakawa Clinic, Japan: Kazuyuki Shirakawa.

Singapore: National Heart Centre, Singapore: Chi Keong Ching, Sandra Thng, Eric Lim, and Yap Pei Wen; National University Hospital, Singapore: Toon Wei Lim, Ying Ming Lee, Winnie Sia, and Vivian Fong; Tan Tock Seng Hospital, Singapore: David Foo, Haiyan Li, Ong See Peng, and Tristen Ng; Changi General Hospital-Parent, Singapore: Kelvin Wong, Siew Yoon Yap, Dan Dan Chen, Geraldine Lim, and Colin Yeo; Singapore General Hospital- Parent, Singapore: Tan Yuyang, Zhi Zi Tan, Wan Tin Lim, and Fazlur Rehman Jaufeerally.

South Korea: Seoul National University Hospital, Seoul, South Korea: Seil Oh, JiHyun Sim, Hye Jin Song, Seungyun Lee, Tae-Eun Kim, and HyunJu Yu; Severance Hospital, Yonsei University, Seoul, South Korea: Hui Nam Park, Mi Suk Lee, Sung Yon Shim, So Hee Joo, Youngmi Park, and Mihyang Park; Kyung Hee University Hospital, South Korea: Woo-Shik Kim, Eunjung Hwang, Eunsun Heo, Jin-bae Kim, and Jung A Kim; Inje University Sanggye Paik Hospital, Seoul, South Korea: HyeYoung Lee, Sun-joo Kim, Inae Han, and Hiyan Ju Kim; The Catholic University of Korea, St. Paul's Hospital, Seoul, South Korea: Sung-Won Jang, Dong Bin Kim, SoYoung Yun, Dong Joon Kim, JinHee An, and JiSuk Kwon; Inha University Hospital, Incheon, South Korea: Dae Hyeok Kim and Kyung Mi Yoo; Pusan National

University Yangsan Hospital, South Korea: Jun Kim, Sang Eun Lee, and Jeongsu Kim; Kangwon National University Hospital, Chuncheon, South Korea: DongRyeol Ryu, KyoungOk Her, and Eun Hee Choi; Korea University Anam Hospital, Seoul, South Korea: Jaemin Shim, Sang Weon Park, Young Ran Jeon, JinYoung Ahn, Gyeong Hui Lee, Hye-Gyeong Jeon, and Woo Jin Chi; Inje University Busan Paik Hospital, Busan, South Korea: Dae-Kyeong Kim, Seula Ye, Min Sun Ha, Sun Min Lee, Jin Hee Lee, Kyung Jin Lee, and Yang-I Kim; Seoul National University Bundang Hospital, Seongnam-si, South Korea: Dong Ju Choi, Eun Hee Lee, Hee Kyung Choi, Woo Yeon Lee, and EunJi Yoon; The Catholic University of Korea, Seoul St. Mary's Hospital, Seoul, South Korea: Yong Seog Oh, Yun Suk Son, Sae Rom Jeon, In-Ae Jung, and Sukyung Park; Chungbuk National University Hospital, Cheongju, South Korea: Myeong-Chan Cho and Chung Suk Lee; Seoul Metropolitan Government Seoul National University, Seoul, South Korea: Hack-Lyoung Kim, Sang Hyun Kim, Sang Hee Kim, EuMi Jang, Miseon Lim, Kyung Lee, Jiyeon Lee, and Bo Ram Park; Pharmacy of The Catholic University of Korea, Uijeongbu St. Mary's Hospital, Uijeongbu, South Korea: Hui-Kyung Jeon, Gyeong Hui Lee, and Ki Tae Kim; Yeungnam University Hospital, Daegu, South Korea: Dong-Gu Shin, Seoyeon Jang, Jung-Min Kim, and Kyung Hui Yim; Hyewon Medical Foundation SeJong General Hospital, Bucheon-si, South Korea: Sang Weon Park, Jin Sik Park, Bomi Kami, Ju-Hee Ahn, Jae Seok Park, and Jung Joo Ahn; VHS Medical Center, Seoul, South Korea: Hoon Ki Park and Hee Suk Choi; Hallym University Sacred Heart Hospital, Anyang-si, South Korea: Sang-Jin Han, Nari Ryu, NaYoung Kwon, and Eun-Joo Choi; CHA Bundang Medical Center, CHA University, Seongnam-Si, South Korea: Jung Hoon Sung, JiSun Kang, So-Young Seo, and HyeKyoung Wi; Chonnam National University Hospital, Gwangju, South Korea: Hyung-Wook Park, JiHyeun Park, Mi Ra Kim, Jeong-Gwan Cho, JiSeon Kim, and Haeim Kim; Asan Medical Center, Seoul, South Korea: Gi-Byoung Nam, Sulhee Lee, and Keun Hye Lee; Samsung Medical Center, Seoul, South Korea: Young Keun On, Sehyun Kim, SoYoung Jo, and Seung Hyun Lee, Hoejeoung Park, Minsuk Rhue, and Min Joo Seo; Korea University Guro Hospital, Seoul, South Korea: Hong Euy Lim, Sang-Min Lee, and SeongWook Hwang; Inje University Ilsan Paik Hospital, Gyeonggi-do, South Korea: JaeJin Kwak, Hyeji Bae, HyeYoung Kye, and JaeJin Kwak; Kosin University Gospel Hospital, Busan, South Korea: Tae-Joon Cha, Eun A Choi, and In Kyoung Noh; Pusan National University Hospital, Busan, South Korea: Taek Jong Hong, JaeHyun Park, JaeKyung Kim, Jeong Hee Moon, Ro Woon Lee, and Kyeonghwa Hyun; Ewha Womans University Mokdong Hospital, Seoul, South Korea: Seong Hoon Park and Mi-Young Lee; Yonsei University Wonju Severance Christian Hospital, Kangwon-do, South Korea: Jung Han Yoon, Jung Ja Woo, Hye-suk Jang, Sunhui Han, Areum Park, Won Sook Son, and Hae Jung An; Wonkwang University Hospital, Iksan-si, South Korea: Nam-Ho Kim, Hae Kyeong Jeong, and Ae Lee Baek; Daegu Catholic University Medical Center, Daegu, South Korea: Kee-Sik Kim, Sung-Guk Kim, Mal-soon Park, Mi Sun Kim, and JuHee Kim; Daegu Fatima Hospital, Deagu, South Korea: Byung Chun Jung and Jung Sook Kim; Ajou University Hospital, Suwon-si, South Korea: Gyo-Seung Hwang, Doo Lae Lee, Kye Ryun Lee, Ji Sun Kang, Joo Yeon Yoo, and Young Mi Sohn; Kyung Hee University Hospital at

Gangdong, Seoul, South Korea: Chong-Jin Kim and Hye-Young Lee; Inha University Hospital, Incheon, South Korea: Dae Hyeok Kim and Kyung Mi Yoo.

Thailand: Burirum Hospital, Muang, Thailand: Sakda Rungaramsin, Riam Inphontan, and Pimolwan Phunpinyosak; Central Chest Institute of Thailand, Muang, Thailand: Peerapat Katekangplu and Piyanuch Sukklad; Inburi Hospital, Inburi, Thailand: Porames Khunrong; Lampang Hospital, Muang, Thailand: Thanita Bunyapipat and Suree Yawila; Maharaj Nakorn Chiang Mai Hospital, Muang, Thailand: Wanwarang Wongcharoen, Arintaya Phrommintikul, and Siriluck Gunaparn; Maharat Nakhon Ratchasima Hospital, Muang, Thailand: Pinij Kaewsuwanna and Thida Chaipermkul; Nakornping Hospital, Mae Rim, Thailand: Khanchai Siriwattana, Theewarut Suttana, and Ketsanee Khunkong; Phramongkutklao Hospital, Rajathevee, Thailand: Waraporn Tiyanon, Thoranis Chantrarat, and Panthipa Bamungpong; Police General Hospital, Pathumwan, Thailand: Supalerk Pattanaprichakul and Usa Kitmapawanont; Ramathibodi Hospital, Ratchathewi, Thailand: Khanchit Likittanasombat, Bandit Naratreekoon, Kolltis Pongmorakot, Somluck Ninwaranon, and Wacharin Suebjaksing; Ratchaburi Regional Hospital, Muang, Thailand: Doungrat Cholsaringkarl; Siriraj Hospital, Bangkoknoi, Thailand: Warangkana Boonyapisit, Napawan Pornnimitthum, and Satchana Pumprueg; Songklanagarind Hospital, Hat Yai, Thailand: Sirichai Cheewatanakornkul, Ratchanee Sirichai, Arthiya Sriwichian, and Treechada Wisaratapong; Srinagarind Hospital, Muang, Thailand: Songkwan Silaruks, Benjaporn Silaruks, and Kittisak Sawanyawisuth; Thammasat University Hospital, Klongluang, Thailand: Pisit Hutayanon, Siripan Hongsuppinyo, and Oranong Thangpet; Uthai Hospital, Uthai, Thailand: Seksan Chawanadelert, Siriwan Ratchasikaew, and Chuleerat Kongsin; King Chulalongkorn Memorial Hospital, Pathumwan, Thailand: Pairoj Chattranukulchai, Rudeewan Khattaroek, and Yongkasem Vorasettakarnkij; Saint Louis Hospital, Sathorn, Thailand: Boonsert Chatlaong; Sunprasitthiprasong Hospital, Muang, Thailand: Yingsak Santanakorn; Banphaeo Hospital, Banphaeo, Thailand: Khompiya Kanokphatcharakun; Maharaj Nakornsrithammarat Hospital, Muang, Thailand: Piya Mongkolwongroj, Janwamol Phangyota, Atthakorn Wutthimanop, and Thanawan Chaiyapong; Chiang Rai Prachanukroh Hospital, Muang, Thailand: Sasivimon Jai-Aue, Kanchana Sanit, and Wattana Wongtheptien; Naresuan University Hospital, Muang, Thailand: Ongkarn Komson, Thanakorn Laksomya, Ampai Tangsirira, Suthasinee Poomiphol, and Prathana Anekpunyakul.

Turkey: Baskent University Istanbul Training and Research Hospital, Istanbul, Turkey: Armagan Altun, Elif Yildrim, Hatice Aysu Ocal, Didar Aslan, Aycan Arslan, and Ozge Ayar; Uludag University Medical Faculty, Bursa, Turkey: Ali Aydinlar, Hulya Saribocek, Gizem Yavasla, Ozlem Aktas, and Nilay Gungor; Erciyes University Medical Faculty, Kayseri, Turkey: Ramazan Topsakal, Ibrahim Ozdogru, Leyla Kasa, Emine Yilmaz, Muruvvet Buyukpapuc, Mustafa Fehmi Birecikliogl u, and Meryem Boyraz; Istanbul University Cerrahpasa Medical Faculty, Istanbul, Turkey: Zeki Ongen, Fuat Polat, Elif Yilirim, Duygu Genc, Baris Ikitimur, Esra Kozig, Sinem Caliskan, Irem Ermec, Busra Gez, Fatih Koyuncuoglu, and Gamze Avinc; Ankara Diskapi Yildirim Beyazit Training and Research Hospital, Ankara, Turkey: Sadik Acikel, Ekrem Yeter, Ahmet Akyel, Mehmet Ali Felekoglu, Asel Canbolat, Deniz

Alpay, Duygu Ozukaya, Yonca Kanal, Ayla Behnejad Kazancik, Faruk Yilmaz, and Tilbe Kundak; Adana Numune Training and Research Hospital, Adana, Turkey: Durmus Yildiray Sahin, Murat Cayli, Hasan Koca, Funda Balci, Hazal Sag, Taner Seker, Alaa Quisi, Berfu Dincyurek, Ilay Cevik, Yasemin Tuccar, and Esra Sunduz Yigittekin; Ondokuz Mayis University Medical Faculty, Samsun, Turkey: Ozcan Yilmaz, Dilek Zorlu, Elvan Ozcelik, and Cansu Kiris; Cumhuriyet University Medical Faculty, Sivas, Turkey: Mehmet Birhan Yilmaz, Funda Aras, and Osman Beton; Inonu University Medical Faculty, Malatya, Turkey: Hasan Pekdemir, Merve Zengin, Refah Karatas, and Murdem Keten; Cukurova University Medical Faculty, Adana, Turkey: Mesut Demir, Vildan Yuksekdag, Aziz Celik, Hatice Rahim, Didem Uzun Alkan, and Gulsum Daglik; Gaziantep University Medical Faculty Sahinbey Educational Research Hospital, Gaziantep, Turkey: Murat Sucu, Erdi Bilecen, Selin Budeyri, and Sinan Bilecen; Hacettepe University Medical Faculty, Ankara, Turkey: Levent Sahiner, Hikmet Yorgun, Ali Oto, Duygu Ozukaya, Murat Ersanli, and Deniz Koca; Memorial Ankara Hospital, Ankara, Turkey: Ali Oto, Sercan Okutucu, Begum Yetis Sayin, Deniz Koca, Asel Conbolat, Deniz Alpay, Duygu Ozukaya, Mine Caglar, and Sebnem Sebnem; Istanbul University Cardiology Institute, Istanbul, Turkey: Murat Ersanli, Sakine Sakiz, Esra Kozig, Sinem Caliskan, Irem Ermec, Busra Gez, Faith Koyuncuoglu, and Gamze Avinc; Bagcilar Training and Research Hospital, Istanbul, Turkey: Ertugrul Okuyan, Ekrem Bilal Karaayvaz, Rifat Yildirim, Sinan Varol, Fatih Kizkapan, Sakine Sakiz, Esra Kozig, Sinem Caliskan, Irem Emrec, Busra Gez, Faith Koyuncuoglu, and Gamze Avinc; Turkiye Yuksek Ihtisas Training and Research Hospital, Ankara, Turkey: Dursun Aras, Didem Civit, Tuba Gulce Dodurga, Asel Canbolat, Deniz Alpay, Duygu Ozukaya, and Yonca Kanal.

Argentina: CENPEC Investigaciones, Ciudad A. de Buenos Aires, Argentina: Florencia Rolandi, Natalia Vensentini, and Vanesa Hansen; Sanatorio NOSTI, Rafaela, Argentina: Adrian Cesar Ingaramo, Gustavo Alberto Ingaramo, Andres Javier Kleiban, Mariana Zillo, Maria Eugenia Yunis, Martin Racca, Carola Ricotti, Luciana Bergesio, and Osvaldo Jose Angel Costamagna; Sanatorio Jozami, La Banda, Argentina: Gustavo Alberto Sambadaro; Clinica Instituto Medico Adrogue, Adrogue, Argentina: Vanina Fernandez Caputi, Fernando Sokn, Jorge Tronge, Andrea Alvarez D'Amelio, Clara Buzzetti, and Pablo Omar Schygiel; Hospital Centro de Salud Zenon Santillan, San Miguel de Tucuman, Argentina: Hector Luciardi; SOS San Bernardo Medicina Prepaga SA, San Miguel de Tucuman, Argentina: Sofia Graciela Berman; Instituto Ave Pulmo, Mar del Plata, Argentina: Pablo Dragotto and Felicitas Fernandez Voena; Sanatorio de la Mujer, Rosario, Argentina: Andres Javier Kleiban, Maria de los Milagros Mercedes Had, and Karina Shatski; Sanatorio Rio Negro, Cipolletti, Argentina: Nestor Centurion and Elena Emilia Piccirilli; Corporacion Medica de Gral. San Martin S.A., San Martin, Argentina: Rodolfo Andres Ahuad Guerrero, Andrea Alvarez D'Amelio, Vanina Campisi, Mariano Fanuele, and Agustina Ahuad Calvelo; Hospital Provincial Enrique F. Erill, Capital Federal, Argentina: Leonel Adalberto Di Paola, Flavio Javier Zurbrigk, and Anabela Martinelli; Sanatorio Mariano Pelliza, Munro, Argentina: Ricardo Dario Dran and Carlos Hector Gimenez; Instituto Medico CER, Quilmes, Argentina: Javier Egido, Alicia Sossich, Fernando Colombo Berra, Cintia Schenkel, Melina Salinger, and Luciana Montoya; Fundacion Favaloro, Buenos Aires, Argentina: Matias Jose Fosco, Enrique Pablo Gurfinkel,

© 2020 Fox KAA et al. *JAMA Network Open.*

Branco Mautner, Simona Malengo, Emilio Daniel Alaguibe, and Lucia Campo; Hospital Interzonal de Agudos Eva Peron, San Martin, Argentina: Victor Alfredo Sinisi; Hospital Italiano de La Plata, La Plata, Argentina: Luis Rodolfo Cartasegna, Andrea Santoro, Maria Fernanda Alzogaray, Viviana Novas, and Jesica Florencia Tinto; Sanatorio San Martin, Venado Tuerto, Argentina: Oscar Gomez Vilamajo, Romina Cabrini, Javier Matkovich, Valentina Garate, and Maria Eugenia Said Palladino; Hospital Italiano Garibaldi, Rosario, Argentina: Jose Luis Ramos, Fabian Diez, Pablo Dragotto, Laura Susana Sanziani, Cintia Martinelli, and Amaru Lopez; Centro Medico Dra Laura Maffei Investigacion Clinica Aplicada, Ciudad Autonoma Buenos Aires, Argentina: Sonia Sassone, Laura Maffei, Miguel Angel Vallejo, Marcelo Yantorno, Lucas Tonelli, Roxana Del Valle Martinez, Maria Goicoechea, Micaela Vallejo, Jesica Pontoriero, Rocio Munguia, Maria Levantini, Flavia Luciana Cilenti, Graciela Beatriz Perez Prados, and Marina Ingratta; Instituto Cardiovascular de Rosario, Rosario, Argentina: Gerardo Zapata, Anibal Agustin Damonte, Luciana Arias, and Alejandro Meirino; Instituto Cardiovascular Buenos Aires, Ciudad Autonoma Buenos Aires, Argentina: Diego Conde, Jorge Atilio Belardi, Ignacio Nicolas De Urquiza, Alexandra Navarro, and Juan Pablo Costabel; Unidad de Cardiologia Clinica, Buenos Aires, Argentina: Guillermo Giacomi, Maria Paula Giacomi, Ana Laura Tufare, Ramon Carrizo, and María Florencia Edén; Sanatorio Modelo Quilmes SA, Buenos Aires, Argentina: Alberto Alfredo Fernandez, Roberto Nicolás Potito, Silvina Borchowiec, Tatiana Bruno, Adrian Demetrio Hrabar, Jorgelina Casala, Claudia Funosas, and Ana Lucia Cappi; Hospital Dr Jose Maria Cullen, Santa Fe, Argentina: Mario Alberto Berli, Paula Berli, Florencia Berli Milagros, Leandro Thomas, Mauricio Priotti, and Germán Maehara; Hospital Universitario Austral, Pilar, Argentina: Fabian Ferroni, Celso Arabetti, Mariana Foa Torres, and Maria Jose Izaguirre.

Brazil: HUOC-UPE–Hospital Universitário Oswaldo Cruz da Universidade de Pernambuco, Recife, Brazil: Dário Celestino Sobral Filho, Libania Ferreira, Tricia Souto Cysneiro, and Eveline Lustosa; Hospital Santa Marcelina, São Paulo, Brazil: Jefferson Jaber, Luis Soares, and Isabel Santos; Instituto Dante Pazzanese de Cardiologia, São Paulo, Brazil: Luciana Vidal Armaganijan, Dikran Armaganijan, Simone Barroso, Kleber Serafim, and Maria Isabel Del Monaco; Hospital Cardiologico Costantini, Curitiba, Brazil: Costantino Roberto Frack Costantini, José Rocha Faria Neto, Carolina Stoll, Danilo Gonçalves Barroso, Costantino Costantini Ortiz, and Daniele Komar; Santa Casa de Misericórdia de Pelotas, Pelotas, Brazil: André Steffens, Camila Fonseca, and Felipe da Silva Paulitsch; Via Médica, Goiânia, Brazil: Weimar Kunz Sebba Barroso de Souzaem and Lohana Borges Queiroz; Hospital de Messejana Dr Carlos Alberto Studart Gomes, Fortaleza, Brazil: João David de Souza Neto and Dafne Lopes; Consultório Dr José Márcio Ribeiro, Belo Horizonte, Brazil: José Márcio Ribeiro; Hospital Cidade, Salvador, Brazil: Marcelo Silveira Teixeira and Denise Guanaes; Núcleo de Pesquisa Clínica S/S, Curitiba, Brazil: Paulo Ross, Verena Araujo, and Luana Herek; Instituto de Cardiologia do Rio Grande do Sul, Porto Alegre, Brazil: Leonardo Pires, Oscar Pereira Dutra, Silvia Poletti, and Rafael de March Ronsoni; ICSC–Instituto de Cardiologia de Santa Catarina, São José, Brazil: Daniel Moreira and Vera Lucia Pereira; Santa Casa De Curitiba, Curitiba, Brazil: José Carlos Moura Jorge, Leonardo Spolaor, Cristaine Dias,

Mariana Rodrigues Pius, and Juliane Woehl; IMC - –Instituto de Moléstias Cardiovasculares de São José do Rio Preto, São José do Rio Preto, Brazil: Adalberto Menezes Lorga Filho, Eduardo Palmegiani, Priscila Fabri, Paula Galeazzi, Thamyres Santini Arroio Cruz, and Clotildes Queirantes; Hospital São Lucas da PUCRS, Porto Alegre, Brazil: Luiz Bodanese, Tulio Ruaro Reichert, Fernanda Chieza, Rosa Vieira Homem, and Ellen Hettwer Magedanz; Hospital Pró-Cardíaco, Rio de Janeiro, Brazil: Marcelo Westerlund Montera and Roberta Perreira; Incor–Instituto do Coração–HCFMUSP, São Paulo, Brazil: Carlos Henrique Del Carlo, Eliane Fernandes, Gabriela Melo, Esther Coste, Maria Fernanda Correia, Cristiano Pisani, Muhieddine Omar Chokr, Hurgo Bellotti Lopes, Ricardo Clemente Mingireanov, Talita Barbosa, Selma Cristina Quaia Fortunato, and Elaine Fernandes; Hospital Felício Rocho, Belo Horizonte, Brazil: Jamil Abdalla Saad, Thiago da Rocha Rodrigues, Barabara Silva, Crystielle Linhares, Maria Celia Marinho, Tânia Félix Lorenzato Fonseca, and Eduardo Belisario Falchetto; Clínica Paulista de Doenças Cardiovasculares Ltda., São Paulo, Brazil: Fernando Augusto Alves da Costa and Raquel Franchin Ferraz; Hospital Sao Paulo, São Paulo, Brazil: Renato Lopes, Anelise Kawakami, Erika Watanabe, and Mayara Vioto Valois; HRAN–Hospital Regional da Asa Norte, Brasilia, Brazil: Gilson Roberto de Araújo and Lenisa Vila Boas; IMV–Instituto De Medicina Vascular Hospital Mae de Deus, Porto Alegre, Brazil: Euler Roberto Manenti, Máurer Pereira Martins, Adriana Silva, Priscila Aparecida Lodi, Aline Borba, Laisa Borrges Ferreira, Anderson Lacerda dos Reis, Nurma Ramos Pereira, Aline de Cassia Vieira dos Santos, Mariana Moraes Pontalti, Alexandre Guerreiro, Ana Paula Macagnan, Livia Pereira, Patricia Ely Pizzato, Milene Santos, and Rafael Luiz Rech; HMCP–Hospital e Maternidade Celso Pierro–PUC-Campinas, Campinas, Brazil: Jose Francisco Kerr Saraiva, Larissa Trama, Carla Vicente, Camila Ormundo, Midia Costa, Tatiana Siqueira, Marina Caporale, and Talita Silva; ICM–Instituto do Coração de Marília, Marília, Brazil: João Carlos Ferreira Braga, Lilian Stefanie da Silva, Daiane Santos, Alexandre Rodrigues, and Fábio Villaça Guimarães Filho; Unicárdio Unidade Clínica e Cardiológica Ltda., João Pessoa, Brazil: Alexandre Negri and Paulo Gottardo; Centro Multidisciplinar de Estudos Clínicos–CEMEC, São Caetano do Sul, Brazil: Carlos Moncada and Marcos Vinicius Seroqui; Sociedade Hospitalar Angelina Caron, Campina Grande do Sul, Brazil: Dalton Precoma, Lucas Geralde, Cibelle Precoma, Thiago Felipe dos Santos, Cristina Paula Corrêa, and Luiz Gustavo Vieira Torres; Hospital Ipiranga, Sao Paulo, Brazil: Fernando Roquette; CARDRESEARCH–Cardiologia Assistencial, Belo Horizonte, Brazil: Gilmar Reis and Ludmila Carvalho; Hospital de Ensino Padre Anchieta, São Bernardo do Campo, Brazil: Roberto Álvaro Ramos Filho, Caroline Nanzer Vital, José Da Silveira, and Rodrigo Pavani; Hospital Lifecenter, Belo Horizonte, Brazil: Estêvão Lanna Figueiredo, Bruna Edilena Paulino Azevedo, Beatriz Quirino, and Gustavo Fonseca Werner; ICT–Instituto do Coração do Triângulo, Uberlândia, Brazil: Roberto Vieira Botelho and Iara Resende; Hospital São Vicente de Paulo, Rio de Janeiro, Brazil: Cláudio Munhoz da Fontoura Tavares, Rogerio Tadeu Tumelero, Gilmar Junior, Norberto Toazza Duda, Leticia Mortari, and Melissa Mazzoni; IPCEM–Instituto de Pesquisa Clínica para Estudos Multicêntricos–Universidade de Caxias do Sul, Caxias do Sul, Brazil: Helius Carlos Finimundi, Silvia Oss Emmer, Fábio Eduardo Camazzola, Edimar Daros, Juliana Anghinoni Andretta, Bruna Nadin, Cibelle

© 2020 Fox KAA et al. *JAMA Network Open.*

Precoma, and Sara Cardoso Boscato; Hospital de Clínicas de Porto Alegre, Porto Alegre, Brazil: Adriano Kochi, Diego Chemello, Juliani da dos Reis, Sabrina Guizzardi, Priscila Agliardi, and Fernanda Igansi; INACTIVE Trymed Biocancer Centro de Pesquisa Clínica S.A., Belo Horizonte, Brazil: César Cássio Broilo França, Jakeline Alves de Oliveira Gomes, Ana Paula Drummond Wainstein, and Edmar Geraldo Ribeiro; IPCARDI–Instituto de Pesquisa Cardiovascular, Caxias do Sul, Brazil: Fábio Alban and Gisele Biazus; HRPC–ULBRA, Canoas, Brazil: Guido Bernardo Aranha Rosito, Heveline Roesch, Larissa Pacheco, Jordana Alba, Tatiana Vargas, Andressa Degen, and Diego Alcoba; Hospital Agamenon Magalhães, Recife, Brazil: João Batista de Moura Xavier Moraes Junior, Fernanda Freire, Sheylla Ribeiro, and Maria Aparecida Torres de Lacerda; Hospital São Vicente de Paulo, Passo Fundo, Brazil: Rogério Tadeu Tumelero, Gilmar Junior, Norberto Toazza Duda, Melissa Mazzoni, Claudio Munhoz da Fontoura Tavares, and Leticia Mortari; Hospital de Medicina de São José do Rio Preto, São José do Rio Preto, Brazil: Lilia Maia, Osvaldo Lourenço da Silva Júnior, Daniele Cristine Esteves, Osana Maria Coelho Costa Mouco, Maria Angelica Benez Teixeira Lemos, Nadielly Codonho Góes, and Marcelo Arruda Nakazone; IMC–Instituto de Moléstias Cardiovasculares Tatuí, Tatuí, Brazil: Roberto Simões de Almeida, Lucas Frare, and Bruna Unterkircher; Vivecor Clínica Médica e Cardiológica–Unidade Campinas, Campinas, Brazil: Ney Carter do Carmo Borges, Fernanda Jannuzzi, and Marco Antonio Dias; ICDF–Instituto de Cardiologia do Distrito Federal, Brasília, Brazil: Luís Gustavo Gomes Ferreira, Etineia Gomes Lino, Arthur Freitas, Tatiane Viana, Manoel Lucas Correa e Silva, and Angela Lopes.

Chile: Centro de Investigaciones Clínicas de la Universidad Católica, Santiago, Chile: Ramón Corbalán, Ivonne Padilla, and Jara Carmen; Centro Medico Stockins y Larenas, Temuco, Chile: Benjamin Aleck Joseh Stockins Fernandez and Carola Lara; Hospital Sotero del Rio, Santiago, Chile: Humberto Montecinos and Yessica Campisto; Centro de Estudios Cardiologicos y de Medicina Interna, Temuco, Chile: Fernando Lanas, Ana Rebolledo, and Elda Molina; Hospital Padre Alberto Hurtado, Santiago, Chile: Martín Larico Gómez; Hospital Carlos Van Buren, Valparaiso, Chile: Carlos Astudillo and Margarita Vergara; Hospital Complejo Asistencial Barros Luco, Santiago, Chile: Carlos Conejeros; Hospital de Talca, Talca, Chile: Patricio Marin Cuevas; Hospital Clinico de la Fuerza Aerea de Chile, Santiago, Chile: Alejandro Forero; Hospital San Juan de Dios La Serena, La Serena, Chile: Claudio Bugueño Gutiérrez and Patricia Cortes; Hospital Clinico de Magallanes Dr Lautaro Navarro Avaria, Punta Arenas, Chile: Juan Aguilar; Hospital Base Osorno, Osorno, Chile: Sergio Potthoff Cardenas and Nelly Garcia Dominguez; Hospital Base Valdivia, Valdivia, Chile: German Eggers and Jessica Munoz Oyarzon; Clinica Santa Lucia, Santiago, Chile: Cesar Houzvic and Veronica Olguin; Hospital del Salvador, Santiago, Chile: Carlos Rey; Hospital Clinico Regional Dr Guillermo Grant Benavente, Talcahuano, Chile: Germán Arriagada and Claudio Villan Araneda; Hospital Naval Almirante Nef, Viña del Mar, Chile: Gustavo Charme Vilches and Ximena Reyes Alvarez.

Mexico: Unidad de Investigacion Clinica en Medicina S.C., Monterrey, Mexico: Carlos Jerjes Sanchez Diaz, Andres Gerardo Ortiz López, Hector David Rodriguez

Flores, and Claudia Atilano Arias; Unidad de Investigacion Clinica Cardiometabolica de Occidente S.C., Guadalajara, Mexico: Jesus Jaime Illescas Diaz, Maria Ruiz Cornejo, Maricela Vidrio Velazquez, Maria Esther Garcia Muñoz, and Marissa Gonzalez; Hospital General Dr Miguel Silva, Morelia, Mexico: Raul Leal Cantu, Pilar Ocampo, Conne Lizbeth Gonzalez Garcia, and Conne Lizbeth Gonzalez Garcia; Consultorio Médico del Dr Ernesto Germán Cardona Muñoz, Guadalajara, Mexico: Maria Guadalupe Ramos Zavala and Sara Pascoe González; Hospital Angeles del Pedregal S.A. de C.V., Mexico, Mexico: Ricardo Cabrera Jardines; The American British Cowdray Centro Medico ABC, Mexico City, Mexico: Nilda Espinola Zavaleta, Victor Roa Castro, and Salome Altamirano Bellorin; Centro de Especialidades Medicas del Estado de Veracruz Dr Rafael Lucio, Jalapa, Mexico: Enrique Lopez Rosas, Isis Lizeth Alarcon Zaragoza, Sukey Ochoa Aybar, and Lucas Solis Morales; Hospital Cardiologica Aguascalientes, Aguascalientes, Mexico: Guillermo Antonio Llamas Esperón, Irma Espinosa, Ana Ramirez Ibarra, Gabriela Romero Cardona, Edgar Eduardo Barcenas Rodriguez, Edgar Eduardo Barcenas Rodriguez, Hector Alejandro Martinez Resendiz, and Maria Elena Felix Hernandez; Instituto de Cardiologia y Medicina Vascular, Monterrey, Mexico: Gerardo Pozas and Priscila Guajardo; Consultorio Médico del Dr Ernesto Germán Cardona Muñoz, Guadalajara, Mexico: Ernesto Cardona Muñoz and Sandra Hernandez Gonzalez; Centro de Investigación cardiovascular, Acapulco, Mexico: Norberto Matadamas Hernandez and Maria Candelaria Mancilla Ortiz; Instituto Nacional de Neurologia y Neurocirugia Manuel Velasco Suarez, Mexico, Mexico: Adolfo Leyva Rendon and Adriana Ortiz; Centro Medico Nacional 20 de Noviembre, Mexico, Mexico: Norberto Garcia Hernandez and Rafael Trujillo Cortes; Centro para el Desarrollo de la Medicina y de Asistencia Medica Especializada S.C., Culiacan, Mexico: Manuel de los Rios Ibarra, Olivia Elena Espinoza Lopez, Luis Gerardo Gonzalez Salas, and Nora Mendoza; Virgen Cardiovascular Research SC, Guadalajara, Mexico: Luis Ramon Virgen Carrillo, Oscar Garcia Garciia, Jose Vega Gonzalez, Humberto Jimenez, and Aldo Guasco Herrera; Unidad de Investigación Clinica en Medicina, S.C., Monterrey, Mexico: David Lopez Villezca; Instituto Cardiovascular de Monclova, S de RL de CV, Monclova, Mexico: Carlos Hernandez Herrera, Daniel Martinez Vasquez, Karla Yaneth Arizpe Aguilar, and Aline Cecilia Meza Ramos; Hospital Angeles Chihuahua, Chihuahua, Mexico: Juan Jose Lopez Prieto; Centro para el Desarrollo de la Medicina y de Asistencia Medica Especializada S.C., Saltillo, Mexico: Rodolfo Gaona Rodriguez and Patricia Padilla Macias; Hospital de Jesus. I.A.P., Cuauhtemoc, Mexico: Efrain Villeda Espinosa, Angelica Villeda, Artuto Gil Sanchez Aguilar, Margarita Abreo, and Miriam Chavarria; Unidad de Investigacion en Salud de Chihuahua SC, Sede Juarez, Ciudad Juarez, Mexico: David Flores Martinez; Hospital Angeles Puebla, Puebla, Mexico: Jose Velasco Barcena; Unidad de Investigacion en Salud de Chihuahua SC, Sede Chihuahua, Chihuahua, Mexico: Omar Fierro Fierro, Maria de la Merced Velazquez Quintana, Jorge Morales, Anais Rascon, Luz Eighty Garcia Prieto, Rosalva Avena, and Lesly Facio; Cardioarritmias e Investigacion S.C., San Luis Potosi, Mexico: Ignacio Rodriguez Briones, Bernardo Vargas Berrueta, Monica Ramos Gonzalez, and Irene Elizabeth Lira González; Hospital Central Dr Ignacio Morones Prieto, San Luis Potosi, Mexico: Jose Luis Leiva Pons, Jorge Carrillo Calvillo, Natalia Nikitina, and Teresa Izquierdo; Hospital de Especialidades

Centro Medico Puerta de Hierro, Zapopan, Mexico: Humberto Alvarez Lopez and Gabriela Santana; Clinica de Estudios Medicos SC, Guadalajara, Mexico: Rafael Olvera Ruiz; Instituto Nacional de Ciencias Médicas y Nutricion Dr Salvador Zubiran, Mexico, Mexico: Carlos Gerardo Cantu Brito and Fernando Flores Silva; Centro de Investigacion Cardiologica EPI M, Mexico, Mexico: Eduardo Julian Jose Roberto Chuquiure Valenzuela; CardioPrevent SC, Durango, Mexico: Roxana Reyes Sanchez, Ricardo Alvarado Ruiz, Lidia Martínez Lares, and Paulina Sida Perez; Centro de Investigacion Alberto Bazzoni S.A. de C.V., Torreon, Mexico: Alberto Esteban Bazzoni Ruiz, Erika Orpinel Almanza, Karla Daniela Monreal de Luna, Aglaé Romo Cerda, Juan Carlos Ubillo Davila, Sandra Roccio Fuentes Ortiz, Rocio Guadalupe Contreras Corona Alejandro Maximiliano Bacelos Lopez, and Jose Luis Recio Gonzalez; Phylasis Clinicas Research S. de R.L. de C.V., Cuautitlan Izcalli, Mexico: Oscar Martin Lopez Ruiz, Fatima Perez, Luis Alberto Martinez Cruz, Julio Alberto Perez Sanchez, Nayeli Soto, Eduardo Montiel, Norma Juarez Garcia, Keren Sharon Perez Mendoza, and Oscar Nandayapa Flores; Centro para el desarrollo de la Medicina y de Asistencia Medica Especializada S.C., sede Mexico, Distrito Federal, Mexico: Roberto Arriaga Nava and Patricia Padilla Macias; Accelerium S de RL de CV, Monterrey, Mexico: Jesus David Morales Cerda, Antonio Salinas, Sergio Piña Toledano, and Euince Aide Fernandez Zuñiga; Centro de Investigacion Cardiovascular y Metabolica, Tijuana, Mexico: Pedro Fajardo Campos, Daniela Cienfuegos, Karina Godoy, Silvia Verdugo Dojaquez, Elena Claudia Mora Respardo, Pablo Alfonso Madero Ayala, Marittza Arasely Uribe Rios, Guadalupe Susan Ramos Garcia, Pedro Fajardo Garcia, Adriana Garcia Mejia, Patricia Ortiz, Glenda Hernandez, and Irene Ortega; Universidad Autonoma de Nuevo Leon, Hospital Universitario Dr Jose Eleuterio Gonzalez, Monterrey, Mexico: Mario Benavides Gonzalez, Jose Esparza Negrete, and Carlos Rivera Ramos.

Austria: LKH–Universitätsklinikum Graz, Graz, Austria: Marianne Brodmann, Thomas Gary, and Claudia Wöhrer; Barmherzige Brueder Konventspital Linz, Linz, Austria: Kurt Lenz; Praxis für Innere Medizin–Dr Claus Hagn–Voels, Voels, Austria: Claus Hagn; Ordination–Dr Foechterle–Linz, Linz, Austria: Johannes Foechterle and Johannes Foechterle; Landeskrankenhaus Feldkirch, Feldkirch, Austria: Heinz Drexel, Karl-Martin Ebner, Abdurahman Said, Daniela Zanolin, Claudia Stöcklöcker, Alexandra Schuler, Alexander Vonbank, Susanne Wäger, Peter Schwerzler, and Helga Winkler; Wilhelminenspital der Stadt Wien, Wien, Austria: Kurt Huber, Claudia Wegmayr, Ioannis Tentzeris, Brigit Vogel, and Johannes Riedl; SMZ Süd–Kaiser Franz Josef Spital Wien, Wien, Austria: Andrea Podczeck-Schweighofer, Fritz Freihoff, and Thomas Priesnitz; Hanusch Krankenhaus der WKGG Wien, Wien, Austria: Michael Winkler and Roland Breier; LKH Kirchdorf, Kirchdorf an der Krems, Austria: Bruno Schneeweiss, Bernadette Bruckner, Thomas Mark and Adrian Mirtl; LKH–Bad Ischl, Bad Ischl, Austria: Alfons Gegenhuber; Krankenhaus der Barmherzigen Brueder Wien, Wien, Austria: Wilfried Lang and Agnes Lischka-Lindner; AKH–Medizinische Universität Wien, Vienna, Austria: Sabine Eichinger-Hasenauer and Lisbeth Eischer; Ordination–Dr Kaserer–Salzburg, Salzburg, Austria: Peter Kaserer; Humanomed Zentrum–Althofen, Althofen, Austria: Josef Sykora; KRAGES Burgenlaendische Krankenanstalten–LH Güssing, Güssing, Austria:

© 2020 Fox KAA et al. *JAMA Network Open.*

Heribert Rasch; LKH–Universitätsklinikum der PMU Salzburg, Salzburg, Austria: Bernhard Strohmer, Uta Hoppe, Lynne Hinterbuchner, and Erika Prinz.

Belgium: Parqué, Jean-Luc, Mouscron, Belgium: Jean-Luc Parqué; BVBA Dr Luc Capiau, Wetteren, Belgium: Luc Capiau, Hilde Capiau, and Farah Banaeian; AZ Sint-Maarten, Mechelen, Belgium: Geert Vervoort, Ellen Deweerd, Erwin Raymenants, Oscar Semeraro, Muriel Delvigne, Tom Vydt, Hans Rombouts, Chris Scheurwegs, Ellen Potoms, Inez Mestdagh, and Sofie Drieghe; ZNA Stuivenberg, Antwerpen, Belgium: Bart Wollaert, Benjamin Scott, Tim Weyn, Emile Keyzer, Marleen Bogaert, Martine Oreglia, Inge Joris, Dirk Denie, Christine Jacobs, and Ingrid Develter; AZ KLINA, Brasschaat, Belgium: Frank Cools, Dieter De Cleen, Steven Hellemans, Johan Salembier, Jef Verheyen, Walter Smolders, Nancy Simons, and Veerle Thyssen; Private Practice Cardiology, De Pinte, Belgium: Geert Hollanders; VZW Regionaal Ziekenhuis Jan Yperman, Ieper, Belgium: Jan Vercammen, Veerle Soufflet, Jan De Keyser, Dries De Cock, and Melissa de Vos; Maria Ziekenhuis Noord-Limburg vzw, Overpelt, Belgium: Dirk Faes, Bert Vanhauwaert, Dirk Van Lier, Cathy Kuppens, and Ann Vandorpe; SPRL MG Balthazar & Ballard, Natoye, Belgium: Yohan Balthazar and Anne Christine Billiaux; Centre Hospitalier Hutois, Huy, Belgium: Marc Delforge, Patrick Maréchal, and Francoise Gits; CHU UCL Namur, Mont-Godinne, Yvoir, Belgium: Olivier Xhaet and Fabien Dormal; Heilig Hart Ziekenhuis, Mol, Belgium: Harry Striekwold and Dirk Vandenbroeck; AZ Turnhout–Campus Sint-Elisabeth, Turnhout, Belgium: John Thoeng; AZ Sint-Lucas–Campus Sint-Lucas, Gent, Belgium: Kurt Hermans, Hans Vandekerckhove, Chantal Smessaert, Hugues Verloove, Katarina Van Beeumen, Jan Nimmegeers, Christophe Borin, Frauke Gorre, An-Kristin Ascoop, and Nancy de Weerdt; Cliniques du Sud-Luxembourg, Arlon, Belgium: Georges Mairesse and Monique Raepers; AZ Delta, Roeselare, Belgium: Wim Anné, Emma Vanhalst, Peter Pollet, Anne Dewispelaere, Florian Demuynck, Martin De Meyer, and Tine Casier; CHU de Charleroi–Hôpital André Vésale, Montigny-le-Tilleul, Belgium: Ivan Blankoff, Veronica Piamonte, and Asuncion Conde Y Bolado; Huisartsen Het Laar, Merksem, Belgium: Michel Beutels, Peter Vandenbossche, Sophie Deckers, and Lisa Helvasto; AZ Zeno, Knokke-Heist, Belgium: Stefan Verstraete, Karine Pieters, Flor Kerkhof, and Ann Dhondt; BVBA Peter Vandergoten Cardiologie, Overijse, Belgium: Peter Vandergoten; Clinique Saint Jean, Brussel, Belgium: Philippe Purnode, Joseph Richa, and Severine Tahon; CHU Ambroise Paré, Mons, Belgium: Pascal Godart, Anna-Maria Barbuto, and Marianne Blockmans; AZ Groeninge–Kennedylaan, Kortrijk, Belgium: Tim Boussy, David Derthoo, Christiaan De Niel, Van Hulle Tim, Hannelore Van Eeckhoutte, Nele Bouckaert, and Marlies De Coninck; CHC–Cliniques de l'Espérance, Montegnée, Belgium: Philippe Desfontaines and Gabriella Tincani; AZ Glorieux, Ronse, Belgium: Alex Heyse, Charlotte Vantomme, Becker Alzand, Frederik Van Durme, Isabelle Stockman, and Marloes Everaert; AZ Nikolaas, Belgium: Joeri Voet, Lies Vergauwen, Martine Lauwers, and Yasmina Steyaert; RZ Heilig Hart Tienen Campus Mariendal, Tienen, Belgium: Axel De Wolf and Chris Brike.

Czech Republic: Ordinace interniho lekarstvi, Praha 2, Czech Republic: Jan Bultas, Hana Lubanda, Milan Zidek, Eva Zidkova, and Alena Lorenzova; Fakultni nemocnice v Motole, Praha 5, Czech Republic: Petr Jansky, Petra Antonova, Jiri Zika, Jana

© 2020 Fox KAA et al. *JAMA Network Open.*

Golova, Alexandra Lindourkova, Jaromir Chlumsky, Petr Potuznik, and Renata Kratochvilova; Nemocnice na Frantisku, Praha 1, Czech Republic: Rudolf Spacek, Lucie Mahdalikova, Lucie Bockova, Adam Sulc, and Lenka Dastychova; InterKardioML s.r.o., Marianske Lazne, Czech Republic: Vilma Machova; Privatni interni ambulance, Brno, Czech Republic: Ondrej Ludka and Alexandra Ludkova; General practitioner s.r.o., Ostrava–Vitkovice, Czech Republic: Josef Olsr and Daniel Michalik; Thomayerova nemocnice, Praha, Czech Republic: Lubos Kotik and Marketa Lajnerova; Nemocnice Slany, Slany, Czech Republic: Blazej Racz, Michaela Horejsi, Lenka Janska, and Tomas Drasnar; Poliklinika Masarykuv Dum, s.r.o, Trutnov, Czech Republic: Richard Ferkl; Privatni interni a kardiologicka ambulance, Chrudim, Czech Republic: Jan Hubac and Vera Hubacova; Ustredni vojenska nemocnice Praha, Praha 6, Czech Republic: Ilja Kotik, Jaroslava Novakova, Petr Krca, and Eva Krcova; Nemocnice Znojmo, Znojmo, Czech Republic: Zdenek Monhart, Eva Prochazkova, and Maria Majerníkova; Poliklinika Humanitas, Bilovec, Czech Republic: Hana Burianova and Pavel Hanzelka; Poliklinika RAVAK, Pribram 8, Czech Republic: Ondrej Jerabek, Martina Valtova, and Iva Kopeckova; Kardio-Pisova, s.r.o., Hradec Kralove, Czech Republic: Jana Pisova and Lenka Chmelickova; Kardiologicka ambulance-Petrova, Usti nad Labem, Czech Republic: Iveta Petrova and Lenka Palubova; Orlickoustecka nemocnice, a.s., Usti nad Orlici, Czech Republic: Vratislav Dedek and Lucie Capova; Avicena–Kardiologie Interna s.r.o., Praha 1, Czech Republic: Michaela Honkova and Jakub Honek; Interni lekarstvi–ALINPED s.r.o., Praha 6, Czech Republic: Petr Podrazil; Krajska zdravotni, a.s.–Nemocnice Teplice, o.z., Teplice, Czech Republic: Petr Reichert and Lenka Zizkova; Fakultni nemocnice Brno, Brno, Czech Republic: Jindrich Spinar and Ruzena Labrova; Fakultni nemocnice u sv. Anny v Brne, Brno, Czech Republic: Miroslav Novak, Jiri Vitovec, Veronika Vyhlidalova, and Jolana Lipoldova; Fakultni nemocnice v Motole, Praha 5, Czech Republic: Vaclav Durdil, Alexandra Lindourkova, and Lucie Riedlbauchova; Nemocnice Novy Jicin a.s., Novy Jicin, Czech Republic: Katarina Plocova, Martin Barsovsky, and Jaroslav Sulitka; Krajska zdravotni, a.s. - Masarykova nemocnice v Usti nad Labem, o.z., Usti nad Labem, Czech Republic: Jiri Lastuvka, Sarka Smetankova, Lenka Zizkova, and Jakub Sveceny.

Denmark: Rigshospitalet, Copenhagen, Denmark: Jørn Nielsen; Århus Universitetshospital, Arhus, Denmark: Steen Husted and Marianne Leth; Bispebjerg Hospital, Copenhagen NV, Denmark: Helena Dominguez and Henrik Nielsen; Sydvestjyds Sygehus Esbjerg, Esbjerg, Denmark: Ulrik Hintze, Anne-Mette Oksbjerg Svenningsen, Joan Hummelshøj, Lene Margrethe Tanggaard, and Annette Nygaard; Hvidovre Hospital, Hvidovre, Denmark: Søren Rasmussen; Næstved Sygehus, Næstved, Denmark: Arne Bremmelgaard and Christina Ellervik; Fredericia Sygehus, Fredericia, Denmark: John Markenvard and Kirsten Vesterager; Lægerne Nordens plads, Frederiksberg, Denmark: Jan Børger; Tønder Lægehus Nord, Tonder, Denmark: Jorgen Solgaard; Lægerne Dommergaarden, Vejle, Denmark: Ebbe Eriksen, Peter Simonsen, and Jakob Tilma; Lægerne Henrichsen Bonde og Løkkegaard, Lyngby, Denmark: Thomas Løkkegaard; Michael Bruun, Struer, Denmark: Michael Bruun; Lægehuset i Jerslev, Jerslev, Denmark: Jacob Mertz; Herlev Hospital, Herlev, Denmark: Morten Schou, Krud Skagen, Helena Dominguez,

and Annie Therkelsen; Frederiksberg Hospital, Fredriksberg, Denmark: Helena Dominguez, Jesper Hansen, Anita Meier, Ilan Raymond, and Dorte Raae; Holbæk Sygehus, Holbæk, Denmark: Michael Olsen, Magnus Jensen, Natasaha Roseva-Nielsen, Lise Jensen, and Therese Bang-Hansen.

Finland: Tampereen yliopistollinen sairaala, Tampere, Finland: Pekka Raatikainen, Kati Helleharju, Hannaleena Nappila, Virpi Palomaki, and Olli Arola; Turun yliopistollinen keskussairaala, Turku, Finland: Juhani Koistinen, Tuija Vasankari, and Carmela Viitanen.

France: Hôpital Nord - CHU Marseille, Marseille, France: Franck Paganelli, Didier Sanchez, Pauline Armangau, Deborah Setbon, Audrey Morioni, and Floriane Robin; Cabinet medical, Bordeaux, France: Joël Ohayon; Groupe Hospitalier Sud–Hôpital Haut-Lévêque, Pessac, France: Frédéric Casassus, Stéphane Lafitte, Eléonore Casassus, and Elodie Ducasse; Hôpital Européen Georges Pompidou, Paris, France: Jean-Yves Le Heuzey; CHU de Toulouse–Hôpital Rangueil, Toulouse, France: Michel Galinier and Nathalie Rosolin; Clinique Saint Joseph–Groupe Hospitalier Prive du Centre Alsace, Colmar, France: Yannick Gottwalles; Hôpital Nord–CHU Marseille, Marseille, France: Franck Paganelli, Didier Sanchez, Audrey Morioni, Floriane Robin, Deborah Setbon, and Pauline Armangau; Centre Hospitalier Public du Cotentin, Cherbourg–Octeville, France: Philippe Loiselet; Cabinet du Dr Jean-Joseph Muller, Strasbourg, France: Jean-Joseph Muller; Centre Hospitalier de Chateauroux, Chateauroux, France: Mohamed Bassel Koujan; CH de Frejus-Saint Raphael, Frejus, France: André Marquand; Centre Hospitalier du Val d'Ariege, Saint Jean de Verges, France: Sylvain Destrac; Centre Cardiologique du Nord, Saint-Denis, France: Olivier Piot and Baptiste Dubois; Centre Hospitalier de Pau, Pau, France: Nicolas Delarche, Josette Couleru, Xavier Giry, Christine Murguet-Badia, and Anne Colomes; Nouvelles Cliniques Nantaises, Nantes, France: Jean-Pierre Cebron and Nathalie Decarsin; Cabinet medical prive, Plan de Cuques, France: Maxime Guenoun; Cabinet prive, Paris, France: Dominique Guedj-Meynier; CH d'Albi, Albi, France: Lokesh A G, Daniel Galley and Christine Beltra; Hôpital Saint Joseph–Paris, Paris, France: Mathieu Zuber and Anne Bonnetain; Hôpital Bichat–Claude Bernard, Paris, France: Pierre Amarenco and Agnes Kemmel; Centre Hospitalier de la côte Basque, Bayonne, France: Emmanuel Ellie; Cabinet prive de cardiologie, Paris, France: James Kadouch; Centre Hospitalier de Beziers, Beziers, France: Pierre-Yves Fournier; Cabinet prive de cardiologie, Houilles, France: Jean-Pierre Huberman; Cabinet prive de cardiologie, Saint André lez Lille, France: Nestor Lemaire; CH de Mulhouse–Hôpital Emile Muller, Mulhouse, France: Gilles Rodier; Centre Hospitalier–La Rochelle, La Rochelle, France: Xavier Vandamme; Groupe Hospitalier Pellegrin–Hôpital Pellegrin, Bordeaux, France: Igor Sibon, Sylvain Ledure, Sabrina Debruxelles, Camille Girollet, Stephane Olindo, Mathilde Poli, Pauline Renou, Sharmila Sagnier, Nathalie Heyvang, and Ledure Sylvain; CHU Poitiers–Hôpital la Milétrie, Poitiers, France: Jean-Philippe Neau; CHU Nice–Hôpital Saint Roch, Nice, France: Marie Hélène Mahagne and Alain Suissa; Cabinet Medical Marseille (GP), Marseille, France: Antoine Mielot; Cabinet Medical, Seysses, France: Marc Bonnefoy; Cabinet Médical, Le Pradet, France: Jean-Baptiste Churet; Cabinet medical, Toulon, France: Vincent Navarre; Cabinet medical privé, Roquevaire, France: Frederic Sellem; Cabinet medical privé,

© 2020 Fox KAA et al. *JAMA Network Open.*

Saint Mandrier sur Mer, France: Gilles Monniot; Cabinet médical privé, Seysses, France: Jean-Paul Boyes; Docteur Bernard Doucet, Wassy, France: Bernard Doucet; Centre Hospitalier de Langres, Langres, France: Michel Martelet; Cabinet medical prive, Aubagne, France: Désiré Obadia; Cabinet prive de cardiologie, Salon de Provence, France: Bernard Crousillat; Centre Hospitalier de Langres, Langres, France: Joseph Mouallem; Centre Hospitalier d'Arras, Arras, France: Etienne Bearez and Corinne Lepot; Cabinet medical–Jean Philippe Brugnaux, Paris, France: Jean Philippe Brugnaux; CHU de Reims, Houilles, France: Alain Fedorowsky; CHU de Reims, Reims, France: Pierre Nazeyrollas; Centre Hospitalier de la côte Basque, Bayonne, France: Jean-Baptiste Berneau, Angeline Barreau, Aurélie Pons, and Estelle Corrihons; APRCL, Bordeaux, France: Frédéric Chemin, Margaux Treuil-Peraldi, Elodie Ducasse, Nadege Ansoult, and and Caroline Moreau.

Germany: Staedtisches Klinikum Dresden Standort Dresden-Friedrichstadt, Dresden, Germany: Sebastien Schellong, Andreas Ulbrich, Thomas Wolf, Carsen Mueller, Johannes Ullrich, Roswitha Frommhold, Kerstin Spranger, Lutz Pomper, Birgit Voigts, Slyvia Weichelt, Juliane Gehre, and Mario Hahn; Vivantes Klinikum Neukoelln, Berlin, Germany: Harald Darius, Friederike Girke, Katja Schirmer, Sandra Gaulke, Thomas Braun, Tanja Toennishoff, Roya Sartipi, Viola Jaerisch, Elke Luczak, Carsten Meincke, Stefan Sommer, and Astrid Maselli; Praxis Dr Koeniger, Ebrach, Germany: Georg Koeniger; Praxis, Berlin, Germany: Andreas Kopf; Gemeinschaftspraxis, Loehne, Germany: Uwe Gerbaulet; Gemeinschaftspraxis, Camburg, Germany: Bernd-Thomas Kellner; Gemeinschaftspraxis Internisten Kelkheim, Kelkheim, Germany: Thomas Schaefer, Gabriele Jass and Sabrina Diez; Gesundheitszentrum Gelstertal, Grossalmerode, Germany: Jan Purr and Ute Weiser; Praxis Dr Eißfeller, Woellstein, Germany: Enno Eißfeller and Karla Kirchner-Volker; Gemeinschaftspraxis Dr Zauzig, Dr Weber, Koeln, Germany: Heinz-Dieter Zauzig; Praxis Dr Riegel, Wolfsburg, Germany: Peter Riegel; Cardiologicum Dresden und Pirna, Dresden, Germany: Christoph Axthelm, Juergen Wurziger, Anja Quietzsch, Janet Geyer, Hartmut Hohensee, Cornelia Fritz, Adrienne Heuer, and Benjamin Schaefer; Praxis Dr Heinz, Bergisch Gladbach, Germany: Gerd-Ulrich Heinz and Afra Heinz; Praxis Dr Menke, Hannover, Germany: Holger Menke; Gemeinschaftspraxis, Berlin-Neukoelln, Germany: Andreas Pustelnik and Sabine Pustelnik; Ihr Landarzt Praxis fuer Familienmedizin, Neubukow, Germany: Stefan Zutz and Andrea Talkenberger; Praxis Dr Eder, Karlsfeld, Germany: Wolfgang Eder and Zuzana Babjakova; Praxis Dr Rehling, Sand am Main, Germany: Guenter Rehling; Praxis Dr med. Glatzel, Hannover, Germany: Dirk Glatzel; Gemeinschaftspraxis Prof. Ludwig, Dr Honl, Willich, Germany: Norbert Ludwig; Praxis Dr med. Sandow, Berlin, Germany: Petra Sandow; Praxis Dr med. Wiswedel, Fuerth, Germany: Henning Wiswedel; Praxis Dr med. Wildenauer, Bad Brueckenau, Germany: Cosmas Wildenauer, Wolfgang Wildenauer, and Svetlana Wildenauer; Klinikum Pirna GmbH, Pirna, Germany: Steffen Schoen, Christoph Reichelt, Steffi Turbanisch, Romy Schoene, Stephanie Zincke, and Marian Christoph; Internistische Facharztpraxis Dr Schwarz, Zwenkau, Germany: Toralf Schwarz and Heike Riessbeck; Praxis Dr med. Babyesiza, Engelskirchen-Ruenderoth, Germany: Adyeri Babyesiza and Regina Voelkel-Babyesiza; Praxis Dr med. Kropp, Luebeck, Germany: Maximilian Kropp and Sylvia Simon; Praxis Dr med. Zimny, Bad Pyrmont, Germany: Hans-Hermann

© 2020 Fox KAA et al. *JAMA Network Open.*

Zimny, Marianne Hintze, and Hildegard Floegel; Gemeinschaftspraxis Dres. Kahl, Ibrom, Fredenbeck, Germany: Friedhelm Kahl; Praxis Dr med. Caspar, Ottenbach, Germany: Andreas Caspar; Praxis Dr med. Omankowsky, Berlin, Germany: Sabine Omankowsky; Gesundheitszentrum Halle-Neustadt, Halle, Germany: Torsten Laessig; Praxis Dr med. Hartmann, Krombach, Germany: Hermann-Josef Hartmann, Diana Amrhein, Kartin Tauber, and Ramona Heeg; Gemeinschaftspraxis Dres. med. Lehmann, Fuldatal, Germany: Gunter Lehmann and Aileen Grytzmann; Praxis Dr Bindig, Geogensgmuend, Germany: Hans-Walter Bindig and Katharina Bindig; Praxis Dr Hergdt, Obermichelbach, Germany: Gunter Hergdt and Susanne Hergdt; Praxis Dr Reimer, Anderbeck, Germany: Dietrich Reimer and Cornelia Reimer; Praxis Dr Hauk, Nussbach, Germany: Joachim Hauk and Christl Hubrich; GP Wuttke/Michel, Lutherstadt Eisleben, Germany: Holger Michel; Praxis Drs Erdle und Budelmann, Ense, Germany: Werner Erdle; Praxis Dr Dorsch, Paderborn, Germany: Wilfried Dorsch and Aneta Tetlak; Praxis Dr Dshabrailov, Osnabrueck, Germany: Janna Dshabrailov; Praxis Dr Rapp, Haigerloch, Germany: Karl-Albrecht Rapp; Fachuebergreifende Praxisgemeinschaft Dr med. H. Boeneke/ R. P. Vormann, Lienen–Kattenvenne, Germany: Reinhold Vormann and Daniela Nicolaus; Praxis Dr. Mueller, Berlin, Germany: Thomas Mueller; Praxis Dr Mayer, Langenfeld, Germany: Peter Mayer; Praxis Dr Horstmeier, Harsewinkel, Germany: Uwe Horstmeier and Jan Zak; Praxis Birkenallee, Papenburg, Germany: Volker Eissing and Gabriele Menken; Gemeinschaftspraxis Dres Hey und Krenzel, Paderborn, Germany: Heinz Hey; Praxis Dr Leuchtgens, Bad Woerishofen, Germany: Heinz Leuchtgens; Praxis Dr Lilienweiss, Kamen, Germany: Volker Lilienweiss; Berufsausübungsgemeinschaft Dres. med. Kolitsch / Mueller, Katzhuette, Germany: Heiner Mueller, Kruth Kolitsch, Anja Fleck, and Katja Bergner; Praxis Dr med. Christian Schubert, Leipzig, Germany: Christian Schubert; Praxis Dr med. Herrmann Lauer, Viersen, Germany: Herrmann Lauer; Praxis Dipl. med. Thomas Buchner, Neustrelitz, Germany: Thomas Buchner; Praxis Dr med. Gunter Brauer, Cottbus, Germany: Gunter Brauer and Diana Kroll; Praxisgemeinschaft Dr med. H. Gliem und FA S. Kamin, Goehren, Germany: Susanne Kamin; Praxis Dr med. Karsten Mueller, Graefenhainichen, Germany: Karsten Mueller and Gabriela Mueller; Fachärztin für Allgemeinmedizin, Apolda, Germany: Sylvia Baumbach and Kerstin Schmidt; Praxis Dr med. Abdel-Qader, Winsen, Germany: Muwafeg Abdel-Qader, Dagmar Mohrmann and Anke Krueger; Gemeinschaftspraxis Stenzel, Ebert, Otto, Riesa, Germany: Hans-Holger Ebert, Lisa Mann, Helen Mortan, Antje Richter, Gunter Stenzel, and Romy Meinecke; Universitaetsklinikum Hamburg-Eppendorf, Hamburg, Germany: Carsten Schwencke, Stefanie Boehme, Manfred Geiger, Detlef Mathey, and Kerstin Flint; Staedtisches Klinikum Dresden Standort Dresden-Friedrichstadt, Dresden, Germany: Sebastian Schellong, Thomas Wolf, Carsen Mueller, Lutz Pomper, Andreas Ulbrich, Juliane Gehre, Johannes Ullrich, Roswitha Frommhold, Kerstin Spranger, Birgit Voigts, Sylvia Weichelt, and Mario Hahn; Universitaetsklinikum Ulm, Ulm, Germany: Peter Bernhardt, Dominik Buckert and Uta Dichristin; FAZ Dresden-Neustadt GbR, Dresden, Germany: Laszlo Karolyi, Yvonne Nickstadt, Kartin Boehme, Christin Rade, Kerstin Bonin, Kerstin Mikes, and Franziska Guenther; Praxis Dr med. Britta Sievers, Henstedt-Ulzburg, Germany: Britta Sievers and Veselin Mitrovic; Charite–Campus Virchow-Klinikum, Berlin, Germany: Wilhelm Haverkamp, Nesrin Nasser, Marika

Saegebarth, Philipp Lacour, Paula Muenkler, Luis Weitbrecht, and Katja Hubert; Vivantes Klinikum Hellersdorf, Berlin, Germany: Jens-Uwe Roehnisch, Susanna Scharrer, Florian Busch, Katja Helgert, Vanessa Klein, Julainne Wettengel, Susanne Zeifele, Carola Ravenhorst, and Maria Schuppe.

Hungary: Szent Rokus Korhaz es Intezmenyei, Budapest, Hungary: Andras Papp; MeDOC EGeszsegkozpont, Budapest, Hungary: Andras Vertes; Szantai MED Bt., Hodmezovasarhely, Hungary: Gabor Szantai; Kanizsai Dorottya Korhaz, Nagykanizsa, Hungary: Andras Matoltsy and Tunde Kiss; UNO Medical Trials Kft., Budapest, Hungary: Nikosz Kanakaridisz; Debreceni Egyetem, Debrecen, Hungary: Zoltan Boda; Tolna Megyei Balassa Janos Korhaz, Szekszard, Hungary: Erno Kis and Jusztina Feil; COROMed-SMO Kft., Pecs, Hungary: Balazs Gaszner; CEE Research Kft., Kisvarda, Hungary: Ferenc Juhasz, Zsuzsanna Hollo and Peter Jen; Dental-Med Co. Bt., Satoraljaujhely, Hungary: Gizella Juhasz, Zsolt Ples, Veronika Forgo, Szilvia Gergely, and Petra Gombos; Gottsegen Gyorgy Orszagos Kardiologiai Intezet, Budapest, Hungary: Sandor Kancz and Eszter Fulop; Szent Janos Korhaz es Eszak-budai Egyesitett Korhazak, Budapest, Hungary: Zoltan Laszlo, Boglarka Vandrus, and Zoltan Radics; Obudai Egeszsegugyi Centrum Kft., Budapest, Hungary: Zsolt May and Fruzsina Sztanyik; Semmelweis Egyetem, Budapest, Hungary: Bela Merkely, Anna Hermecz, Marianna Szabo, and Nora Sydo; Fejer Megyei Szent Gyorgy Egyetemi Oktato Korhaz, Szekesfehervar, Hungary: Ebrahim Noori and Maria Hollósiné Kovács; Pecsi Tudomanyegyetem, Pecs, Hungary: Tamas Habon, Kalman Toth, and Szilvia Kovacsne Levang; SzSzB Megyei Korhazak es Egyetemi Oktatokorhaz, Nyiregyhaza, Hungary: Peter Polgar and Attila Szilagyi; Sopron Medical Egeszsegugyi Szolgaltato Kft., Csorna, Hungary: Gabriella Szalai; Belinus Bt., Debrecen, Hungary: Sandor Vangel; Bacs-Kiskun Megyei Korhaz, Kecskemet, Hungary: Andras Nagy; QUALICLINIC Eu-i Szolg. es Kutatasszervezo Kft, Budapest, Hungary: Gabriella Engelthaler, Istvan Szombati, Gabor Bogye, and Eszter Kiralyhazine Gyorke; Clinexpert Kft., Budapest, Hungary: Judit Ferenczi, David Gulyas, Emese Dohovits, Anette Toth, Reka Szalo, and David Cseresznyek.

Italy: Azienda Socio Sanitaria Territoriale degli Spedali Civili di Brescia (Presidio Spedali Civili), Brescia, Italy: Giuliana Martini; Arcispedale S. Maria Nuova Azienda Ospedaliera di Reggio Emilia, Reggio Emilia, Italy: Leone Maria Cristina and Attilia Pizzini; Ospedale degli Infermi, Rimini, Italy: Eros Tiraferri; Azienda Ospedaliera Pugliese Ciaccio, Catanzaro, Italy: Rita Santoro; Azienda Socio Sanitaria Territoriale di Cremona (Istituti Ospitalieri di Cremona), Cremona, Italy: Sophie Testa and Cinzia Zecca; Azienda Ospedaliera Universitaria “Federico II,” Napoli, Italy: Giovanni Di Minno and Pasquale Ambrosino; Fondazione IRCCS CA' Granda Ospedale Maggiore Policlinico, Milano, Italy: Marco Moia and Simon Braham; Azienda Socio Sanitaria Territoriale Niguarda (Grande Ospedale Metropolitano Niguarda), Milano, Italy: Teresa Maria Caimi, Roberta Fritella, Daniela Piazzolla, Roberta Frittella, Elena Mollica Poeta, and Stefania Brusorio; Azienda Ospedaliero-Universitaria “Policlinico-Vittorio Emanuele,” Catania, Italy: Maria Tessitori; Azienda Ospedaliera di Perugia Ospedale S. Maria della Misericordia, Perugia, Italy: Giancarlo Agnelli, Federica Macellari, Erika D’Agostini, and Marta Fedele; A.O.U. Senese Policlinico Santa Maria alle Scotte, Siena, Italy: Roberto Cappelli and Michele Voglino; Azienda

© 2020 Fox KAA et al. *JAMA Network Open.*

Ospedaliera Universitaria Careggi, Firenze, Italy: Daniela Poli; Azienda Ospedaliero Universitaria di Parma, Parma, Italy: Roberto Quintavalla and Piera Maria Ferrini; Ospedale della Val di Chiana Santa Margherita, Cortona, Italy: Franco Cosmi; IRCCS Ospedale Casa Sollievo della Sofferenza, San Giovanni Rotondo, Italy: Raffaele Fanelli, Raimondo Massaro, and Carmela DArienzo; Azienda Ospedaliera Bianchi Melacrino Morelli, Reggio Calabria, Italy: Vincenzo Oriana, Gaterina Latella, and Gianluca Sottilota; Presidio Ospedaliero SS. Trinità Sora, Sora, Italy: Raffaele Reggio; Azienda Ospedaliera Nazionale Santi Antonio e Biagio e Cesare Arrigo, Alessandria, Italy: Roberto Santi; Ospedale di Bentivoglio, Bentivoglio, Italy: Leonardo Pancaldi and Giulio Boggian; Policlinico Universitario Agostino Gemelli, Roma, Italy: Raimondo De Cristofaro and Leonardo Di Gennaro; Azienda Ospedaliera Universitaria Policlinico Sant'Orsola Malpighi, Bologna, Italy: Giuliana Guazzaloca, Gualtiero Palareti, and Luisa Salomone; Ospedale Santa Maria Goretti, Latina, Italy: Angelo De Blasio and Carlo Ciabatta; Azienda Socio Sanitaria Territoriale Sette Laghi (Presidio Ospedale di Circolo e Fondazione Macchi), Varese, Italy: Jorge Salerno Uriate, Vincenzo Guerrieri, Benedetta Montanari, Alfredo Bianchi, and Riccardo Gorla; Ospedale San Paolo, Savona, Italy: Flavia Lillo, Franca Minetti and Cinzia Scarone; Azienda Socio Sanitaria Territoriale di Monza (Presidio San Gerardo), Monza, Italy: Enrico Maria Pogliani and Monica Carpenedo; Istituto Auxologico Italiano -I.R.C.C.S., Milano, Italy: Grzegorz Bilo and Luca Grappiolo; Azienda Ospedaliera Card. G. Panico, Tricase, Italy: Michele Accogli and Simona Longo; Presidio Ospedaliero ASUR-Senigallia, Senigallia, Italy: Antonio Mariani, Nino Ciampani, Francesca Calcagnoli, and Anna Patrignani; ASL CN1 Ospedale Maggiore Strada Statale Trinità, Fossano, Italy: Mauro Feola; Fondazione IRCCS Policlinico San Matteo, Pavia, Italy: Arturo Raisaro and Katharina Granzow; Ospedale San Giuseppe e Melorio, Santa Maria Capua Vetere, Italy: Luciano Fattore and Cosimo Nave; Azienda Sanitaria Locale 2 Olbia, Olbia, Italy: Andrea Mauric; USL 2 Umbria - Distretto di Perugia, Perugia, Italy: Fabrizio Germini, Giuliana Duranti, Gregorio Baglioni, Valerio Pannacci, Fulvio Forcignanò, and Angelo Giombolini; AUSL 2 Umbria, Perugia, Italy: Luca Tedeschi, Maria Settimi, and Mario Berardi; Medicina di Gruppo–Borgo di Terzo, Bergamo, Italy: Sergio Nicoli; ASL Arezzo, Arezzo, Italy: Paolo Ricciarini and Roberto Nasorri; ASL CN 1, Cuneo, Italy: Antonio Argena, Pierdomenico Bossolasco and Bartolomeo Allasia; AUSL Parma, Parma, Italy: Paolo Ronchini; ASL Bergamo, Bergamo, Italy: Claudio Bulla, Luciano Foppa, Alessandro Filippi, Maria Lusia Bottarelli, and Antonino Tomasello; ASL Milano, Milano, Italy: Filippo Tradati; Azienda Ospedaliera Sant'Andrea-Università di Roma La Sapienza, Roma, Italy: Massimo Volpe; Azienda Ospedaliera di Rilievo Nazionale A. Cardarelli, Napoli, Italy: Maria D'Avino and Domenico Caruso; Azienda Ospedaliero Universitaria Pisana, Pisa, Italy: Maria Grazia Bongiorni and Luca Segreti; Presidio Ospedaliero della Misericordia, Grosseto, Italy: Silva Severi and Marco Breschi; Azienda Ospedaliero Universitaria Ospedali Riuniti, Torrette di Ancona, Italy: Alessandro Capucci, Federico Guerra, Kate Pozniak Graziela Rangel, and Marta Marcinekova; Istituto Clinico Humanitas, Rozzano, Italy: Corrado Lodigiani, Elena Banfi, and Veronica Pacetti; ASL 3 Genovese di Genova, Genova, Italy: Enrico Salomone; Azienda Ospedaliero Universitaria Ospedali Riuniti di Foggia, Foggia, Italy: Gaetano Serviddio, Rosanna Villani, and Aurelio Lo Buglio;

Centro Cardiologico Monzino-IRCCS, Roma, Italy: Claudio Tondo, Viviana Biagioli, and Eleonora Russo; Azienda Ospedaliera di Perugia Ospedale S. Maria della Misericordia, Terni, Italy: Giuseppe Ambrosio, Giuseppe Ciliberti, and Ezio Mesolella; Azienda Ospedaliera S. Anna e S. Sebastiano, Caserta, Italy: Paolo Golino and Giovanni Cimmino; Azienda Servizi Sanitari 1 Triestina, Trieste, Italy: Carmine Mazzone and Andrea Di Lenarda; Anthea Hospital, Reggio Calabria, Italy: Saverio Iacopino, Giuseppe Campagna Pierpaolo Occhilupo, and Roberta Spirito.

The Netherlands: Stichting Trombosedienst Maastricht, Maastricht, Netherlands: Hugo ten Cate and Marieke Pavlicic; Noordwest Ziekenhuisgroep, Alkmaar, Netherlands: J.H. Ruiter; St. Jans Gasthuis, Weert, Netherlands: Andreas Lucassen and H.C. Klomps; Trombosedienst Apeldoorn-Zutphen, Apeldoorn, Netherlands: Henk Adriaansen, Mirjam Debordes, and Jan Jaap van Putten; Trombosedienst Ziekenhuis Rivierenland, Tiel, Netherlands: Maarten Bongaerts, Laurens Westerman, Jannie Boon, and Tineke Kloosterman; Stichting Cardiologie Amsterdam, Amsterdam, Netherlands: Mathijs Pieterse, Misannia Boersma-Slootweg, Salcudean Tania, Stan Vergos, and Kris Gorrebeeck; WECOR, Etten-Leur, Netherlands: Coen van Guldener; Onze Lieve Vrouwe Gasthuis, Locatie Oost, Amsterdam, Netherlands: Johannes Herrman, Femmy Bosman, Sara Dols, Laura Breukel, Jennifer Knaake, Sander Niehe, Elise Van Dongen, Sandra Bruin, and A.J.K. Roelse; Bethesda Diabetes Research Center, Hoogeveen, Netherlands: S.H.K. The, Josien Krikken, Eileen Van Warners, and Floor Geerlings; Franciscus Gasthuis, Rotterdam, Netherlands: P.R. Nierop, Ingrid Danse, Nel Slingerland, Jurgen Akkerhuis, Sweder Van de poll, Marja van der Knaap, Irene Kort, and Margaret Dirks; Diaconessenhuis Meppel, Meppel, Netherlands: Pieter Hoogslag, J.H. Geertman, and Agaath Stallinga-de Vos; ETZ Elisabeth, Tilburg, Netherlands: Walter Hermans, Paul Melman, Tim van der Kley, Guido van Leeuwen, A. Pronk, Riny van de Loo, and Jeanne de Graauw; Gelre Ziekenhuizen, Apeldoorn, Apeldoorn, Netherlands: B.E. Groenemeijer, Anastazia Jerzewski, E. M. Koomen, Suzanne Jansen, and Rina Mulder; Slingeland Ziekenhuis, Doetinchem, Netherlands: W. Terpstra, J.M.C. Van Hal, and Anneke Grunewald; Huisartsenpraktijk Ewijk, Ewijk, Netherlands: Cees Buiks and Paul Buiks; St. Antonius Ziekenhuis, Nieuwegein, Netherlands: L.V.A. Boersma, Pim Tonino, Jur ten Berg Josine te Kaat, Wesley Jetten, Malu Kelderman, Judith De Graaf, Mike Bosschaert, Boudewijn Uppelschoten, Irene Vogel, Donia Mohamed, and Madeleine van der Perk.

Norway: Oslo Universitetssykehus HF, Ullevål, Oslo, Norway: Ingrid Dominguez, Dan Atar, Bjørn Løvås, Trude Berge, Eivind Berge, and Hege Claussen; Østlandske Hjertesenter, Moss, Norway: Per Anton Sirnes and Vivi Nilsen; Asker Legesenter, Asker, Norway: Erik Gjertsen; Ålesund Hospital, Ålesund, Norway: Torstein Hole, Rigmor Bøen, and Silje Rasmussen; Vestlandske Hjertesenter AS, Bergen, Norway: Knut Erga; Spesialistgruppen, Nesttun, Norway: Arne Hallaråker and Marie O'Donovan; Trondheim Hjeresenter, Trondheim, Norway: Gunnar Skjelvan and Sissel Strand; Gransdalen Legesenter, Oslo, Norway: Anders Østrem; Løvenstadtunet Legesenter, Løvenstad, Norway: Beraki Ghezai; Nymoen Legekontor (General Practice), Kongsberg, Norway: Arne Svilaas and Kristin Ringdalen; Stavanger Medisinske Senter AS, Stavanger, Norway: Peter Christersson; Hallset legesenter,

Trondheim, Norway: Torbjørn Øien and Aase Jekthammer; Langbølgen legesenter, Oslo, Norway: Svein Høegh Henrichsen and Anne Berit Lensebraaten; Sykehuset i Vestfold HF, Tønsberg, Norway: Jan Erik Otterstad and Karin Ausen; Vestfold Hjertesenter, Sandefjord, Norway: Jan Berg-Johansen and Hege Antonsen.

Poland: Instytut Kardiologii im. Prymasa Tysiaclecia Kardynala Stefana Wyszynskiego, Warszawa, Poland: Janina Stepinska, Elzbieta Kremis, and Anna Konopka; SPZOZ w Lubartowie, Lubartow, Poland: Andrzej Gieroba; Indywidualna Specjalistyczna Praktyka Lekarska Małgorzata Biedrzycka, Starogard Gdanski, Poland: Malgorzata Biedrzycka and Lukasz Biedrzycki; Prywatny Gabinet Lekarski, Piotrkow Trybunalski, Poland: Michal Ogorek and Aleksandra Szczepanska; Wojewodzki Szpital Zespolony w Kielcach, Kielce, Poland: Beata Wozakowska-Kaplon, Radoslaw Bartkowiak, Kamila Wesolowska, Rafal Bzymek, and Elzbieta Jaskulska-Niedziela; Dolnoslaski Szpital Specjalistyczny im.T.Marciniaka- Centrum Medycyny Ratunkowej, Wroclaw, Poland: Krystyna Loboz-Grudzien, Edyta Szuchnik, Magdalena Dziuba, Barbara Rzyczkowska, and Maria Loboz-Rudnicka; Mazowiecki Szpital Specjalistyczny Sp. z o.o., Radom, Poland: Jaroslaw Kosior and Ewa Pawlik-Rak; Samodzielny Publiczny Szpital Wojewodzki, Gorzow Wielkopolski, Poland: Wieslaw Supinski, Anna Szulowska, and Jerzy Bartnik; Szpital Wojewodzki nr 2 w Rzeszowie, Rzeszow, Poland: Jerzy Kuzniar, Elzbieta Korczowska, and Janusz Romanek; Mazowiecki Szpital Specjalistyczny im. Dr Józefa Psarskiego w Ostrołęce, Ostroleka, Poland: Roman Zaluska, Janusz Jankielewicz, Dariusz Andrzejewski, Dorota Kruczyk, Przemyslaw Chojnowski, Wojciech Rogowski, and Adam Komlo; Niepubliczny Zaklad Opieki Zdrowotnej, Nowa Sol, Poland: Jaroslaw Hiczkiewicz and Wojciech Faron; NZOZ–Przychodnia Zespolu Lekarzy Rodzinnych w Szamocinie, Szamocin, Poland: Lucyna Swiatkowska-Byczynska and Wojciech Wieczorek; Szpital Specjalistyczny im. S. Zeromskiego SPZOZ, Krakow, Poland: Lech Kucharski, Joanna Araminowicz, Diana Kociolek, Agnieszka Orda, and Adriana Jaremczuk-Kaczmarczyk; Specjalistyczne Centrum Medyczne CORDIMED, Gdansk, Poland: Marcin Gruchala, Bartosz Curyllo, and Anna Frankiewicz; Miejskie Centrum Medyczne im. dr Karola Jonschera w Łodzi, Lodz, Poland: Piotr Minc, Justyna Rychta, Jolanta Niedek, Halina Krystyna Kowalczyk, and Karol Cieslak; NZOZ Amamed, Warszawa, Poland: Maciej Olszewski and Pawel Szalecki; Clinmedica Research OMC, Skierniewice, Poland: Grzegorz Kania, Pawel Wojewoda, and Agnieszka Sidor; Zespol Opieki Zdrowotnej, Ostrowiec Swietokrzyski, Poland: Malgorzata Krzciuk, Piotr Walasik, Anna Szpotowicz, Anna Barszcz, and Malgorzata Ozgowicz; COPERNICUS Podmiot Leczniczy Sp. z o. o., Gdansk, Poland: Zbigniew Lajkowski; Wojskowy Instytut Medyczny, Warszawa, Poland: Bozenna Ostrowska-Pomian, Agata Krzesiak-Lodyga, Ewelina Kowal, Anna Jackun-Podlesna, and Katarzyna Kalin; Wojewodzki Szpital Specjalistyczny we Wroclawiu, Wroclaw, Poland: Jerzy Lewczuk, Marzena Stopyra-Poczatek, Agata Kaczmarzyk-Radka, Magdalena Wilgat-Szecowka, Renata Romaszkiewicz, and Daniel Blaszczyk; Szpital Wojewodzki im. M. Kopernika w Koszalinie, Koszalin, Poland: Elzbieta Zinka, Agata Pawelska-Buczen, Karolina Zakutynska-Kowalczyk, and Anita Biernacka; Indywidualna Specjalistyczna Praktyka Lekarska dr n. med. Agnieszka Karczmarczyk Specjalista Chorob, Szczecin, Poland: Agnieszka Karczmarczyk and Robert Kaliszczak; I Szpital Miejski im dr

© 2020 Fox KAA et al. *JAMA Network Open.*

E.Sonnenberga, Lodz, Poland: Malgorzata Chmielnicka-Pruszczynska and Dorota Lesniewska-Krynska; SPSK Nr 7 SUM w Katowicach Gornoslaskie CM im. prof. Leszka Gieca, Katowice, Poland: Iwona Wozniak-Skowerska, Maria Trusz-Gluza, and Seweryn Nowak; Samodzielny Publiczny Centralny Szpital Kliniczny, Warszawa, Poland: Grzegorz Opolski, Agnieszka Kolodzinska, and Marek Kiliszek; Szpital Wielospecjalistyczny im. dr Ludwika Blazka w Inowroclawiu, Inowroclaw, Poland: Marek Bronisz, Jacek Szafranski, Artur Jarzebowski, Malgorzata Brzustowska, and Marcin Mielcarek; NZOZ ALL-MED Centrum Medyczne Specjalistyczne Gabinety Lekarskie, Lodz, Poland: Marcin Ogorek, Nikolina Roszczyk, Dorota Rozewska-Furmanek, and Pawel Ptaszynski; NZOZ Medicus Sp. z o.o., Gliwice, Poland: Grazyna Glanowska and Przemyslaw Wilczewski; Szpital Wojewodzki im. Prymasa Kardynala Stefana Wyszynskiego, Sieradz, Poland: Piotr Ruszkowski, Jerzy Leszczynski, Monika Smichura, and Mateusz Splawski; Wojewodzki Szpital Zespolony im. L. Rydygiera w Toruniu, Torun, Poland: Grzegorz Skonieczny, Krystyna Jaworska, Lukasz Bernat, Anna Raczynska, Malgorzata Troszczynska, Agnieszka Metzgier-Gumiela, Maria Jaworska-Drozdowska, Tomasz Traczyk, Grzegorz Trzcinski, and Ryszarda Piotrowicz; Szpital Powiatowy SP ZOZ, Olawa, Poland: Ryszard Sciborski, Ewa Bekieszczuk, Beata Dolecka and Joanna Starak-Marciniak; SP CSK im. prof. K. Gibinskiego SUM, Katowice, Poland: Boguslaw Okopien, Malgorzata Klata, Iwona Kobielusz-Gembala, Marcin Basiak, Witold Szkrobka, and Tomasz Dybala; Szpital Specjalistyczny im.H.Klimontowicza w Gorlicach, Gorlice, Poland: Piotr Kukla, Wojciech Kurdzielewicz, Elzbieta Broton, Maciej Kluczewski, and Marcin Czamara; Medicus Bonus Sp z o.o., Wagrowiec, Poland: Krzysztof Galbas and Agata Markiewicz; Indywidualna Specjalistyczna Praktyka Lekarska w Dziedzinie Kardiologii lek. med. Krzysztof Cymerman, Gdynia, Poland: Krzysztof Cymerman and Alina Cieszynska; Poradnia Kardiologiczna Jarosław Jurowiecki, Gdansk, Poland: Jaroslaw Jurowiecki, Monika Machnikowska, Jolanta Neubauer-Geryk, Sebastian Tybura, and Monika Figura-Chmielewska; NZOZ Pro-Cordis Sopockie Centrum Bad. Kardiolog., Sopot, Poland: Pawel Miekus; NZOZ Centrum Medyczne HCP, Poznan, Poland: Waldemar Myszka, Agata Nowak, Aleksandra Wierzbicka, and Karolina Majewska; Centrum Medyczne Medyk, Rzeszow, Poland: Stanislaw Mazur, Ewa Miedlar, Monika Ambicka, Karolina Bugajska, Anna Starok, Artur Chmielowski, and Michal Mazur; Lecznice Citomed Sp. z o.o., Zespol Przychodni, Torun, Poland: Roman Lysek, Grazyna Jaguszewska, Aleksandra Lysek-Jozefowicz, Grazyna Szumczyk-Muszytowska, and Lukasz Wojnowski; NZOZ Przychodnia Specjalistyczna “Medica” Bogdan Walko, Lublin, Poland: Jacek Baszak, Bogdan Walko, Malgorzata Jargiello-Baszak, and Wojciech Brzozowski; NZOZ Vitamed, Bydgoszcz, Poland: Teresa Rusicka-Piekarz, Andrzej Galaj, Malgorzata Zyczynska-Szmon, Waldemar Gadzinski, Joanna Luka, Rafal Cichomski, and Marian Krzyzanowski; Uniwersyteckie Centrum Kliniczne, Gdansk, Poland: Grzegorz Raczak, Ewa Staniszewska, Pawel Gutknecht, Agnieszka Niemirycz-Makurat, Michal Wrobel, Ewa Kochanska, Mateusz Sciborski, Sebastian Tybura, Ewa Lewicka, Ludmila Danilowicz-Szymanowicz, Malgorzata Szwoch, Monika Figura-Chmielewska, Jolanta Skalska, Barbara Opielowska-Nowak, Lukasz Drelich, Justyna Kabat, and Beata Dudzik-Richter; Centrum Medyczne CDS, Wroclaw, Poland: Ewa Domanska, Malgorzata Guziewicz, Dorota Kustrzycka-

Kratochwil, and Malgorzata Sukiennik-Kujawa; Krakowski Szpital Specjalistyczny im. Jana Pawla II, Krakow, Poland: Jadwiga Nessler, Ewelina Lichota, Ewa Gasior, Marta Wegrzynowska, Pawel Rostoff, and Rafal Mariankowski; Kutnowski Szpital Samorzadowy Sp. z o.o., Kutno, Poland: Jozef Lesnik.

Russia: Medical Centre "Alians," Kirovsk, Russia: Vera Eltishcheva; SBEI HPE "Orenburg State Medical University" of the MoH of the RF, Orenburg, Russia: Roman Libis, Irina Kulchenkova, and Natalia Shkatova; SAIH "Republican Clinical Hospital #2," Kazan, Russia: Gadel Kamalov; SBIH of Novosibirsk Region "Clinical Emergency Hospital #2," Novosibirsk, Russia: Dmitry Belenky, Olga Volodicheva, and Olga Kungurtseva; StP SHI Clinical hospital of St. Luka, Saint-Petersburg, Russia: Liudmila Egorova; SBEI HPE "Yaroslavl State Medical University" of the MoH of the RF, Yaroslavl, Russia: Alexander Khokhlov, Olga Sinitsina, Svetlana Speshilova, Svetlana Chugunnaya, and Elena Lileeva; State Autonomous Healthcare Institution "City Clinical Hospital #7," Kazan, Russia: Eduard Yakupov; SBIH of Moscow "City Clinical Hospital # 17," Moscow, Russia: Dmitry Zateyshchikov and Irina Zotova; FSBI "Scientific-research Institute for Complex Problems of Cardiovascular Disease," Kemerovo, Russia: Olga Barbarash, Evgeniya Zhuravleva, Elena Gorbunova, Tatyana Gorshkova, Yulia Belenkova, and Tatiana Kupriyanova; SBIH of Novosibirsk Region "Clinical Emergency Hospital #2," Novosibirsk, Russia: Olga Miller, Olga Kungurtseva, and Marina Blinkova; SHI "Regional Clinical Hospital," Tver, Russia: Evgeniy Mazur, Dmitry Platonov, and Yurij Orlov; SPb SBIH "City Hospital # 38 n.a. N.A. Semashko," Saint-Petersburg, Russia: Konstantin Zrazhevskiy; SPb SBIH "City Pokrovskaya Hospital," Saint-Petersburg, Russia: Tatyana Novikova and Fatima Bitakova; City Clinical Hospital n.a. Botkin, Moscow, Russia: Yulia Moiseeva; SHI "Center of Occupational Pathology," Saint-Petersburg, Russia: Elena Polkanova; SBIH of Moscow "City Clinical Hospital #61," Moscow, Russia: Konstantin Sobolev and Svetlana Erofeeva; TSBIH "Krasnoyarsk Interdistrict Clinical Hospital of Emergency Medical Care n.a. N.S. Karpovich," Krasnoyarsk, Russia: Maria Rossovskaya; DCH on Station Chelyabinsk of JSC "Russian Railways," Chelyabinsk, Russia: Yulia Shapovalova and Ashot Agakhanyan; FSBI "Polyclinic #3" of the Department for Presidential Affairs of the RF, Moscow, Russia: Alla Kolesnikova; FSBSI "Scientific Research Institute of Therapy and Preventive Medicine," Novosibirsk, Russia: Konstantin Nikolaev and Alla Ovsyannikova; FSBI "Russian Cardiological Scientific and Industrial Complex" of the MoH of the RF, Moscow, Russia: Oksana Zemlianskaia, Elizaveta Panchenko, and Ekaterina Kropacheva; SBIH of Moscow "City Clinical Hospital # 51," Moscow, Russia: Anna Zateyshchikova and Ekaterina Volchkova; SPb SBIH "City Polyclinic #109," St. Petersburg, Russia: Victor Kostenko; FSBI "Research Institute for Cardiology" of Siberian Branch RAMS, Tomsk, Russia: Sergey Popov and Roman Batalov; FSAEI HE "First Moscow State Medical University n.a. I.M. Sechenov" of the MoH of the RF, Moscow, Russia: Maria Poltavskaya, Anait Dumikyan, and Olga Machilskaya; LLC "Alliance Biomedical–Russian Group," Saint-Petersburg, Russia: Anton Edin and Tamara Kolesova; FSBI "Polyclinic # 5" of the Department for Presidential Affairs of the RF, Moscow, Russia: Elena Aleksandrova and Olga Kropova; FSAEI HE "First Moscow State Medical University n.a. I.M. Sechenov" of the MoH of the RF, Moscow, Russia: Oksana Drapkina, Vladimir Ivashkin, and Olga Korneeva; SPb

© 2020 Fox KAA et al. *JAMA Network Open.*

SBIH “City Pokrovskaya Hospital,” Saint-Petersburg, Russia: Alexander Vishnevsky and Pavel Karchicyan; FSBMEI HPE “Military Medical Academy n.a. S.M. Kirov” of the MoD of the RF, St. Petersburg, Russia: Oleg Nagibovich, Galina Nagibovich, Galina Monako, and Elena Kuznetsova; Therapeutic Department of Clinical Hospital n.a. N.A. Semash, Yaroslavl, Russia: Petr Chizhov, Elena Novikova, Tatyana Medvedeva, Yulia Ivanova, and Marina Gurmach; RBIH “Cardiological Dispensary,” Ivanovo, Russia: Svetlana Rachkova, Olga Lebedeva, Elena Shutemova, Svetlana Romanchuk, and Margarita Ovchinnikova; SPb SBHI “City Policlinic #74,” Kronshtadt, Russia: Mikhail Sergeev, Daria Konyushenko, and Polina Kuchuk; Alupka City Hospital, Alupka, Russia: Borys Kurylo; State Institution “Crimean State Medical University Named After S.I. Georgievskiy,” Simferopol, Russia: Alexey Ushakov.

Spain: Hospital de la Santa Creu i Sant Pau, Barcelona, Spain: Xavier Vinolas, Enrique Rodriguez F, Jose Mara Guerra, Concepcion Alonso, Marcos Rodriguez Garcia, Douglas Alvarez, Anna Espallargas, Enrique Pena Garcia, and Francisco Mendez Zurita; Policlinic Viladecans, Viladecans, Spain: Pere Alvarez Garcia, Nieves Palomo Merchan, and Vianeth Rios; Complejo Hospitalario Universitario A Coruña, A Coruña, Spain: Maria Fernanda Lopez Fernandez, Beatriz Herrero Maeso, Maria del Carmen Gomez Castillo, Marta Fernandez, Diego Otero Tomera, Javier Batlle Fonrodona, Joana Costa Pinto Prego de Faria, and Teresa Martinez; Hospital Universitario Virgen de las Nieves, Granada, Spain: Luis Tercedor Sanchez, Alejandro Molina Leyva, Rosa Macias, Manuel Molina, Miriam Jimenez, Francisco Jose Bermudez Jimenez, Daniel Castro Fernandez, Maria Jose Jimenez Fernandez, Mercedes Cabrera Ramos, Miguel Alvarez, Maria Molina Jiménez, and Carolina Torres; Centro de Salud El Cristo, Oviedo, Spain: Salvador Tranche Iparraguirre, Norberto Sierra, and Joaquin Aracil Villar; EAP Mataro-6 (Gatassa-El Maresme), Mataro, Spain: Pere Toran Monserrat, Antonio Negrete Palma, Stela Sanchez Parra, and Josep Sorribes Lopez; Centro de Salud la Orden, Huelva, Spain: Emilio Marquez Contreras; CAP Llança, Llança, Spain: Jordi Isart Rafecas and Stela Sanchez Parra; Centro de Especialidades Virgen de la Cinta, Huelva, Spain: Juan Motero Carrasco; Hospital Universitario Puerta de Hierro Majadahonda, Majadahonda, Spain: Pablo Garcia Pavia, Mateo Cordoba, Ana Briceno Hinojo, Maria del Carmen Gutierrez del Val, and Ariadna Gonzales Segovia; Complejo Hospitalario Ruber Juan Bravo, Madrid, Spain: Casimiro Gomez Pajuelo, Manuela Pereda Armayor, Miguel Perez Carasa, and Carmela Alonso; Hospital Universitario Ramon y Cajal, Madrid, Spain: Luis Miguel Rincon Diaz, Miguel Castillo Orive, Paz Gonzalez, and Irene Lasuncion; Hospital Universitario de Burgos, Burgos, Spain: Luis Fernando Iglesias Alonso and Carolina Santolaya; Hospital Universitario Severo Ochoa, Leganes, Spain: Angel Grande Ruiz; Hospital Universitari de Tarragona Joan XXIII, Tarragona, Spain: Jordi Merce Klein, Miriam Garcia Bermudez, Veronica Quintern, and Elisabet Serralvo; Complejo Hospitalario Universitario de Santiago, Santiago de Compostela, Spain: Jose Ramon Gonzalez Juanatey, Lilian Grigorian, Pilar Mazon, Venesa Garcia Millan, Ana Seoane Blanco, and Maria Moure Gonzalez; Hospital Universitario Virgen del Rocio, Sevilla, Spain: Gonzalo Baron Esquivias, Marga Gavira Saenz, and Mariela Campo Moreno; C.A.P. Can Vidalet, Esplugués de Llobregat, Spain: Ines Monte Collado; C.A.P. Sitges, Stiges, Spain: Herminia Palacin Piquero and Stela Sanchez

Parra; CAP Sardenya, Barcelona, Spain: Carles Brotons Cuixart; C.A.P. Horta, Barcelona, Spain: Esther Fernandez Escobar, Maria Rodriguez Morato, Carmen Rodrigo, Maribel Martines Mena, Ana Domenech Borras, Maria Teixido Fontanillas, Miguel Casanova Gil, Maria Ubeda Pastor, Aileen Austria, Edelmira Barraquer Feu, Xavier Robiro Robiro, and Stela Sanchez Parra; C.A.P. El Clot, Barcelona, Spain: Joan Bayo i Llibre, Carme Roca Saumell, Maria Rosa Senan Sanz, and Stela Sanchez Parra; Hospital Universitario Central de Asturias, Oviedo, Spain: Cecilia Corros Vicente and Norberto Sierra; Hospital Virgen del Mar, Almeria, Spain: Manuel Vida Gutierrez; Corporacio Sanitaria Parc Tauli, Sabadell, Spain: Francisco Epelde Gonzalo and Roser Renom; Complejo Asistencial de Avila, Avila, Spain: Carlos Alexandre Almeida Fernandez and Sara Saez Jimenez; Centre d'Atencio Primaria Hostalets de Balenya, Hostalets de Balenyà, Spain: Encarnacion Martinez Navarro, Nuria del Val Plana, Gemma Tobajas, Marc Roca, and Laia Riquelme Sola; CAP Doctor Subirós, La Jonquera, Spain: Jordi Isart Rafecas, Enrique Escriva Montserrat, Stela Sanchez Parra, and Inma Pareja Ibar; EAP Mataró-3 (Rocafonda–Palau), Mataro, Spain: Juan Jose Montero Alia, Pilar Montero Alia, Ana Ferrer, and Stela Sanchez Parra; Centro de Salud Paulino Prieto, Oviedo, Spain: Maria Barreda Gonzalez and Norberto Sierra; Centre d'Atencio Primaria El Remei, Vic, Spain: Maria Angels Moleiro Oliva, Manel Terns Riera, Roger Codinachs Alsina, Marta Dachs, and Anna Bartes; CS de Ventanielles, Oviedo, Spain: Jose Iglesias Sanmartin, Norberto Sierra and Aida Iglesias Garcia; EAP Mataró-1 (La Riera), Mataro, Spain: Mercedes Jimenez Gonzalez, Pilar Montero Alia, Stela Sanchez Parra, and Juan Jose Montero Alia; CAP Canet de Mar, Canet de Mar, Spain: Maria del Mar Rodriguez Alvarez, Eva Calvo Martinez, Anna Cortada Cabrera, Monserrat Olle Borque, Rolando Armitano Ochoa, Martin Cebollada del Misterio, Johan Cabeza Ramirez, Victoria Marina Ortega, Carmen Gines Garcia, Antonio Branjovich Tijuan, Eugeni Fernandez Mas, Stela Sanchez Parra, Benardi Jimeno Besa, and Rosa Blanca Munoz Munoz; CAP La Llantia, Mataró, Spain: Juan Herreros Melenchon; Hospital Son Llatzer, Palma de Mallorca, Spain: Tomas Ripoll Vera, Joan Torres Marques, Javier Fosch, Salvador Marcus, Catalina Melia, and Yolanda Gomez Perez; Hospital Clinico Universitario Virgen de la Victoria, Malaga, Spain: Manuel Jimenez Navarro, Miguel Lopez, Gloria Millan Vazquez, Natalia Andere, Lola Gomez, Hugo Nelson Orellana Figueroa, Victor Becerra Munoz, and Alicia Guerrero Molina; Policlinico de Vigo-Povisa, Vigo, Spain: Maria Vazquez Caamano, Marcelo Sanmartin Fernandez, and Luisa Sanchez Mendez; Hospital de Basurto, Bilbao, Spain: Maria Fe Arcocha Torres, Izaskun Prieto, Aranza Manzanal Rey, and Ane Elorriaga Madariaga; Hospital San Pedro de Alcantara, Caceres, Spain: Gonzalo Marcos Gomez, Carolina Ortiz Cortes, Javier Mendoza Vazquez, Pablo Sanchez Calderon, Luis Enrique Lezcano Gort, Carmen Simon Valero, Gema Cancho Corchado, and Maria Isabel Cotilla Marco; Hospital Meixoeiro, Vigo, Spain: Andres Iniguez Romo, Maria Belen Lage Bouzamayor, Alicia Martin Vila, Marta Llobet Molina, Carlos Maria Diaz Lopez, Marisol Amaro, Saleta Barbeira, Pablo Juan Salvadores, Diana Montes, and Seila Costas; Centro de Salud Vallobin-La Florida, Oviedo, Spain: Miguel Angel Prieto Diaz, Eduardo Hevia Rodriguez, and Norberto Sierra.

Sweden: Karolinska Universitetssjukhuset i Solna, Stockholm, Sweden: Mårten Rosenqvist; Andersbergs Hälsocentral, Gävle, Sweden: Alexander Wirdby;

Centrumkliniken, Stockholm, Sweden: Jan Lindén; Capio vårdcentral Ringen, Stockholm, Sweden: Kerstin Henriksson; Vårdcentralen Norrmalm, Skövde, Sweden: Micael Elmersson; Vårdcentralen Eda, Charlottenberg, Sweden: Arnor Egilsson; Landstinget Dalarna, Gagnef, Sweden: Karin Floren and Ulf Börjesson; Kvarnsvedens vårdcentral, Borlänge, Sweden: Gunnar Svärd; S3 Clinical Research Centers, Vällingby, Sweden: Susanna Dzeletovic and Bo Liu; Husläkarna Österåker, Åkersberga, Sweden: Anders Lindh and Rose-Mari Kangert; Vårdcentralen Kristinehamn, Kristinehamn, Sweden: Lars-Bertil Olsson and Catarina Grässjö; Kils Vårdcentral, Kil, Sweden: Mikael Gustavsson; Sunne vårdcentral, Sunne, Sweden: Lars Andersson; Luthagens specialistmottagning, Uppsala, Sweden: Lars Benson, Karina Rosenberg, and Ann Mannermyr; Husläkarna i Margaretelund, Åkersberga, Sweden: Claes Bothin; Hagakliniken, Göteborg, Sweden: Ali Hajimirsadeghi, Mina Shayesteh, Ruth Lissledal, and Jessica Nyström; Arvika sjukhus, Arvika, Sweden: Björn Martinsson and Birgitta Jansson; Vårdcentralen Hjorten, Trollhättan, Sweden: Marianne Ericsson; Stockholm Heart Center, Stockholm, Sweden: Åke Ohlsson, Lisa Bastani, Kristina Skoglund, Faris Al-Khalili, and Pia Löf; Karlskoga lasarett, Karlskoga, Sweden: Håkan Lindvall and Ann-Britt Ekstrand; Skånes Universitetssjukhus, Malmö, Sweden: Peter Svensson and Camilla Nilsson; Capio vårdcentral Lidingö, Lidingö, Sweden: Katarina Thörne; Capio vårdcentral Solna, Solna, Sweden: Hans Händel; Skånes Universitetssjukhus, Lund, Lund, Sweden: Pyotr Platonov and Anna Osberg; Östersunds Sjukhus, Östersund, Sweden: Fredrik Bernstenand, Orjan Stromqvist, Björn Eriksson, Agneta Lindberg, and Annica Olofsson; Hässleholms Sjukhus, Hässlehom, Sweden: Ingar Timberg and Mari Stjernberg; Karolinska Trial Alliance (KTA) PRIM, Stockholm, Sweden: Milita Crisby, Helena Aaröe, Katarina Risbecker, Daniel Bengtsberg, Sirin Gudmundsson, and Annelie Billger; Länssjukhuset Ryhov, Jönköping, Sweden: Jan-Erik Karlsson, Dawid Kusiak, Grzegorz Bonkowski, Annika Koch, and Jessika Samuelsson; Ljungby Lasarettet, Ljungby, Sweden: Agneta Andersson, Marita Millborg, and Ann-Marie Ohlin; Närsjukhuset Köping, Köping, Sweden: Lennart Malmqvist, Gull-Britt Eriksson, and Susanne Hahn; Länssjukhuset Halmstad, Halmstad, Sweden: Johan Engdahl and Lisbeth Andersson; Capio Citykliniken Lund, Lund, Sweden: Jörgen Thulin and Carl-Johan Lindholm; Hjärt&Kärlcentrum, Södertälje, Sweden: Aida Hot-Bjelak, Eva Ahbeck, and Marie Aman; Blekingesjukhuset Karlshamn, Karlshamn, Sweden: Steen Jensen, Katarina Mansson, and Carl Thorsen; Centralsjukhuset Karlstad, Karlstad, Sweden: Per Stalby, Johan Lugnegård, Hakan Ahlmark, Anna Pedersen, and Annika Lettenström.

Switzerland: Universitaetsspital Zuerich, Zurich, Switzerland: Jan Steffel, Ellen Saga, and Stephan Winnik; Spital Uster, Ulster, Switzerland: Johann Debrunner, Daniela Amstutz, and Alexander Westphalen; Kantonsspital Baden AG, Baden, Switzerland: Juerg H. Beer, Alexandra Grau, Isberg Henriette, Christian Gustav Lutz, Kirsten Steden, Laura Boos, Philipp Baumgartner, Jana Efe, Marcel Frick, and Emanuel Aegerter; Geneva University Hospital, Genève 14, Switzerland: Dipen Shah, Jasmine Bruegger, Alexandre Guinand, and Guillermet Elise.

Ukraine: GI L.T.Malaya Therapy National Institute of the NAMS of Ukraine, Kharkiv, Ukraine: Iurii Rudyk, Valeriya Nemtsova, Olena Medentseva, and Sergiy

Pyvovar; CI of Healthcare Kharkiv CCH #8 Dept of Therapy Kharkiv MA of PGE of MOHU, Kharkiv, Ukraine: Vira Tseluyko and Olga Romanenko; Kyiv City Clinical Hospital #1, Kyiv, Ukraine: Oleksandr Karpenko, Liudmyla Todoriuk, and Svitlana Kizim; Cherkasy Regional Cardiological Center, Cherkasy, Ukraine: Svitlana Zhurba and Olga Proshak; Railway Transport Kharkiv CH #1 of Healthcare Center Branch of PJSC Ukr Railway, Kharkiv, Ukraine: Igor Kraiz and Ellina Kamenska; SI NSC M.D. Strazhesko Institute of Cardiology of NAMSU, Kyiv, Ukraine: Oleksandr Parkhomenko and Oleksandr Shumakov; CI Ivano-Frankivsk Reg Cl Card Center Dept of Arterial Hypertension SHEI Ivano-Frankivsk NMU, Ivano-Frankivsk, Ukraine: Iryna Kupnovytska, Irina Kutynska, Roxolana Belegai, and Andriy Sapatyi; Ivano-Frankivsk RCCD Dept of Anesthesiology with ITR SHEI Ivano-Frankivsk NMU, Ivano-Frankivsk, Ukraine: Nestor Seredyuk and Roman Petrovskyy; Private Small Enterprise, Medical Center Pulse, Vinnytsia, Ukraine: Yuriy Mostovoy, Olha Mostova, and Lesia Rasputina; City Clinical Hospital #6 of Emergency Medical Care, Simferopol, Ukraine: Oleksiy Ushakov; C.J.Em.H.,Card.dept.for treat.of pat.with myoc.infar.#2,DSMA, Dnipro, Ukraine: Olena Koval, Pavlo Kaplan and Andrii Ivanov; City Hospital #1, Mykolaiv, Ukraine: Igor Kovalskiy and Dmitry Plevak; M.D. Strazhesko Institute of Cardiology of AMS of Ukraine, Kyiv, Ukraine: Yevgeniya Svyshchenko, Larysa Bezrodna, Olena Matova, Tetiana Ovdiienko, and Maryna Mospan; SI NSC M.D. Strazhesko Institute of Cardiology of NAMSU, Kyiv, Ukraine: Oleg Sychov and Olena Romanova; National Pirogov Memorial Medical University, Vinnytsia, Ukraine: Mykola Stanislavchuk, Galyna Berko, and Liudmyla Burdeina; CI CCH #6, Zaporizhzhia, Ukraine: Oleg Kraydashenko, Vladislav Varenov, Roman Stets, and Alina Khmelyova; Lutsk City Hospital, Lutsk, Ukraine: Andriy Yagensky and Mykhailo Pavelko; OSMU of Min.of Health of Ukr.,Ch.of In.Med.#2,Un.cl.of OSMU, Odesa, Ukraine: Susanna Tykhonova and Olena Khyzhnyak; CI Zapor CCH#10 SI ZMA PGE Ch of therapy clin pharmacol & endocrin, Zaporizhzhia, Ukraine: Ivan Fushtey, Valentyn Mochonyi, Olena Chabanna, Inna Daniuk, and Olexandr Palamarchuk.

United Kingdom: Laurie Pike Health Centre, Aston, United Kingdom: Will Murdoch; River Brook Medical Centre, Stirchley, United Kingdom: Naresh Chauhan; Wand Medical Centre, Highgate, United Kingdom: Daryl Goodwin; Greenridge Surgery, Billesley, United Kingdom: Louise Lumley; Grange Hill Surgery, Kings Norton, United Kingdom: Ramila Patel; Ridgacre House Surgery, Quinton, United Kingdom: Philip Saunders; The Greens Health Centre, Dudley, United Kingdom: Bennett Wong; Marysville Medical Practice, Belle Vue, United Kingdom: Alex Cameron; The Nechells Practice, Birmingham, United Kingdom: Philip Saunders; The Village Surgery, Shirley, United Kingdom: Niranjan Patel; Kingsbury Road Surgery, Erdington, United Kingdom: P. Jhittay; Northfield Health Centre, Northfield, United Kingdom: Andrew Ross; Primrose Lane Surgery, Low Hill, United Kingdom: M. S. Kainth; Dovecote Surgery, Oldbury, United Kingdom: Karim Ladha; Claremont Medical Practice, Exmouth, United Kingdom: Kevin Douglas; The Newbridge Surgery, Wolverhampton, United Kingdom: Gill Pickavance; Wychall Lane Surgery, Kings Norton, United Kingdom: Joanna McDonnell; King Street Surgery, Hereford, Hereford, United Kingdom: Laura Handscombe; The Surgery, Atherstone, Atherstone, United Kingdom: Trevor Gooding; Rising Brook Health Centre, Rising Brook, United

Kingdom: Helga Wagner; Saville Medical Group, Newcastle upon Tyne, United Kingdom: Cumberlidge; Marsden Medical Practice, South Shields, United Kingdom: Colin Bradshaw; Guide Post Medical Group, Chappington, United Kingdom: Catherine Bromham; Oxford Terrace Medical Group, Gateshead, United Kingdom: Kevin Jones; The Surgery, Bilston, Bradley, United Kingdom: Shoeb Suryani; Seaton Park Medical Group, Ashington, United Kingdom: Richard Coates; Northgate Practice, Anchor Meadow, United Kingdom: Bhupinder Sarai; Rolle Medical Partnership, Claremont Grove, United Kingdom: W Willcock; The Surgery, Cannock, United Kingdom: S. Sircar; Library House Surgery, Chorley, United Kingdom: John Cairns; The Elmwood Practice, Belfast, United Kingdom: A Gilliand; Dr Bilas, Wolverhampton, United Kingdom: Roman Bilas; Penn Manor Medical Practice, Penn, United Kingdom: E Strieder; Rother House Medical Centre, Stratford-Upon-Avon, United Kingdom: Peter Hutchinson; Russell House Surgery, Codsall, United Kingdom: Anne Wakeman; Sandy Lane Surgery, Rugeley, United Kingdom: Michael Stokes; Warders Medical Centre, Tonbridge, United Kingdom: Graham Kirby; Westgate Surgery, Westgate-on-Sea, United Kingdom: Bhaskhar Vishwanathan; Beaconsfield Medical Practice, Brighton, United Kingdom: Nigel Bird; Wish Park Surgery, Hove, United Kingdom: Paul Evans; Cranleigh Surgery, Cranleigh, United Kingdom: M Clark; Cathedral Medical Group, Chichester, United Kingdom: John Bisatt; Pound Hill Medical Group, Pound Hill, United Kingdom: Jennifer Litchfield; Park Surgery, Horsham, United Kingdom: E. Fisher; Pulborough Medical Group, Pulborough, United Kingdom: Tim Fooks; Worden Medical Centre, Leyland, United Kingdom: Richard Kelsall; Sandbach GPs, Sandbach, United Kingdom: Neil Paul; Barton Surgery (Dawlish), Dawlish, United Kingdom: Elizabeth Alborough; Broadway Medical Centre, Broadway, United Kingdom: Michael Aziz; Bellevue Medical Centre, Fleetwood, Fleetwood, United Kingdom: C Ramesh; Bollington Medical Centre, Bollington, United Kingdom: Pete Wilson; Budleigh Salterton Medical Centre, Budleigh Salterton, United Kingdom: Simon Franklin; Cleveleys Group Practice, Cleveleys, United Kingdom: Sue Fairhead; Cossington House Surgery, Canterbury, United Kingdom: Julian Thompson; Creffield Medical Centre, Colchester, United Kingdom: Hasan Chowan; Taylor & Partners, Woodbridge, United Kingdom: Gary Taylor; Ely Bridge Surgery, Ely, United Kingdom: John Wakeling; Firsway Health Centre, Sale, United Kingdom: Dawn Tragen; Harvey Group Practice, St Albans, United Kingdom: Matt Parfitt; The Surgery, Honiton, Honiton, United Kingdom: Claire Seamark; Kiltearn Medical Centre, Nantwich, United Kingdom: Carolyn Paul; Lindum Medical Practice, Lincoln, United Kingdom: Mark Richardson; Litchdon Medical Centre, Barnstaple, United Kingdom: Angus Jefferies; Magdalen Medical Practice, Norwich, United Kingdom: Helen Sharp; Mattishall Surgery, Mattishall, United Kingdom: Hywel Jones; Mount Farm Surgery, Bury St Edmunds, United Kingdom: Claire Giles; The Fleetwood Health Centre (prev Mountview Surgery), Fleetwood, United Kingdom: Matthew Bramley; Nettleham Medical Practice, Nettleham, United Kingdom: Philip Williams; Portmill Surgery, Hitchin, United Kingdom: Jehad Aldegather; Queen Square Medical Services Ltd, Lancaster, United Kingdom: Simon Wetherell; Sedbergh Medical Practice, Sedbergh, United Kingdom: William Lumb; St Leonard’s Practice, Exeter, United Kingdom: Phil Evans; St Stephens Gate Medical Practice, Norwich, United Kingdom: Frances

© 2020 Fox KAA et al. *JAMA Network Open.*

Scouller; Stowhealth Limited, Stowmarket, United Kingdom: Neil Macey; The Rise Group Practice, Hornsey Rise, United Kingdom: Stephen Rogers; Victoria Road Surgery, Lowestoft, Oulton Broad, United Kingdom: Yvette Stipp; Woolpit Health Centre, Woolpit, United Kingdom: Richard West; Wymondham Medical Partnership, Wymondham, United Kingdom: Philip Pinney; Ash Trees Surgery, Carnforth, United Kingdom: Paul Wadeson; Crescent Surgery, Cleveleys, United Kingdom: John Matthews; Village Practice, Thornton-Cleveleys, United Kingdom: Preeti Pandya; Rosebank Medical Practice, Lancaster, United Kingdom: Andrew Gallagher; Brown Clee Surgery, Bridgnorth, United Kingdom: T Railton; Waterfront Medical Centre, Barry, United Kingdom: Emyr Davies; Whinpark Medical Practice, Edinburgh, United Kingdom: Jonathan McClure; Edzell Health Centre, Edzell, United Kingdom: Marc Jacobs; Mauve Practice, Perth, United Kingdom: Claire Hutton; Bridgewater Family Medical Practice, Whitchurch, United Kingdom: R Thompson; The Medical Centre, Boston, Old Leake, United Kingdom: Bijoy Sinha; Tasburgh Lodge Surgery, Woodhall Spa, United Kingdom: Keith Butter; Lakeside Healthcare at St. Mary's Medical Centre, Stamford, United Kingdom: Susan Barrow; The New Sheepmarket Surgery, Stamford, United Kingdom: Helen Little; Mount Pleasant Health Centre, Exeter, United Kingdom: David Russell; Mill Bank Surgery, Stafford, United Kingdom: Ulka Choudhary; Winyates Health Centre, Redditch, United Kingdom: Ikram Haq; Sherbourne Medical Centre, Leamington Spa, United Kingdom: Paul Ainsworth; Spring Gardens Group Medical Practice, Spring Gardens, United Kingdom: Claire Jones; Framfield House Surgery, Woodbridge, United Kingdom: Phil Weeks; Marden Medical Practice, Shrewsbury, United Kingdom: Jane Eden; St Thomas Health Centre, Exeter, United Kingdom: Lisa Gibbons; Belvoir Vale Surgery, Bottesford, United Kingdom: Janet Glencross; Bay Medical Group (prev Coastal), Morecambe, United Kingdom: Alison MacLeod; The Montalto Medical Centre, Ballynahinch, United Kingdom: K. Poland; Nottinghill Medical Practice, Newtownabbey, United Kingdom: Conor Mulolland; Lisburn Health Centre, Lisburn, United Kingdom: A Warke; Ballygomartin Group Practice, Belfast, United Kingdom: Paul Conn; Duncairn Medical Practice, Belfast, United Kingdom: D Burns; Birchwood Medical Practice, Lincoln, United Kingdom: R Smith; Newmarket Medical Practice, Louth, United Kingdom: R Kamath; The Butchery Surgery, Sandwich, United Kingdom: Jonathan Webster; Buckfastleigh Medical Centre, Buckfastleigh, United Kingdom: Ian Hodgins; Ide Lane Surgery, Exeter, United Kingdom: Stephen Vercoe; Kingswood Surgery, Tunbridge Wells, United Kingdom: Paul Roome; Whitstable Medical Practice (Estuary View), Whitstable, United Kingdom: Hilary Pinnock; Downsway Medical Practice, Istead Rise, United Kingdom: Jayesh Patel; Oakenhurst Medical Practice, Blackburn, United Kingdom: Amar Ali; Crossgar Surgery, Crossgar, United Kingdom: Nigel Hart; Bideford Medical Centre, Bideford, United Kingdom: Richard Davies; Coleridge Medical Centre, Ottery Saint Mary, United Kingdom: Nigel De-Sousa; East Cliffe Practice, Ramsgate, United Kingdom: Catherine Neden; Pool Health Centre, Redruth, United Kingdom: Mark Danielsen; Gravesend Medical Centre, Gravesend, United Kingdom: Purnima Sharma; Steyning Health Centre, Steyning, United Kingdom: Sophia Galloway; Beccles Medical Centre, Beccles, United Kingdom: Charlotte Hawkins; Cromer Group Practice, Cromer, United Kingdom: Raife Oliver; Bouch & Partners,

© 2020 Fox KAA et al. *JAMA Network Open.*

Lowestoft, United Kingdom: Martin Aylward; The Manor Practice, Sutton Coldfield, United Kingdom: Mira Pattni; Grove Surgery (Thetford), Thetford, United Kingdom: Gordon Irvine; Fakenham Medical Practice, Fakenham, United Kingdom: Shahid Ahmad; Llandaff North Medical Centre, Cardiff, United Kingdom: Catherine Rothwell; Mexborough Health Centre, Mexborough, United Kingdom: Fiaz Choudhary; Attleborough Surgeries, Norfolk, United Kingdom: Sabrina Khalaque; Saffron Group Practice, Leicester, United Kingdom: Stephanie Short; Hobs Moat Medical Centre, Solihull, United Kingdom: Sharon Peters; Albany House Medical Centre, Northampton, United Kingdom: Warwick Coulson; Leicester Terrace Health Centre, Northampton, United Kingdom: Neil Roberts; Danetre Medical Centre, Daventry, United Kingdom: Amy Butler; Wychbury Medical Group, Wollescote, United Kingdom: Steven Coates; Bovey Tracey & Chudleigh Practice, Chudleigh, United Kingdom: Ben Ward; Greensands Medical Practice, Gamlingay, United Kingdom: Daniel Jackson; Stonecroft Medical Centre, Sheffield, United Kingdom: Steve Walton; Dearne Valley Group Practice, Thurnscoe, United Kingdom: Diane Shepherd; Westbank Practice, Starcross, United Kingdom: Toh Wong; Conisbrough Group Practice, Doncaster, United Kingdom: Mark Boon; Brannam Medical Centre, Barnstaple, United Kingdom: Melanie Deacon; St Andrews Surgery, Tonypandy, United Kingdom: David Cornelius; Haltwhistle Medical Group, Haltwhistle, United Kingdom: Sarah Davies; Sele Medical Group, Hexham, United Kingdom: Ben Frankel; Burn Brae Medical Group, Hexham, United Kingdom: Nick Hargreaves; Drs Cloak, Choi and Milligan, The Green, United Kingdom: Henry Choi; Dr Stephenson & Partners, Concord, United Kingdom: Jon Sumner; Rothwell & Desborough Surgery, Rothwell, United Kingdom: Tim Myhill; Oak Street Medical Practice, Norwich, United Kingdom: Salah Estifanos; Greens Norton Medical Centre, Towcester, United Kingdom: Diane Geatch; The Scott Practice, Balby, United Kingdom: Justin Wilkinson; Brunel Medical Practice, Babbacombe, United Kingdom: Richard Veale; Bentley Surgery, Doncaster, United Kingdom: Karen Forshaw; Yealm Medical Centre, Yealmpton, United Kingdom: Rob Hirst; Danes Camp Surgery, Northampton, United Kingdom: Kashif Zaman; Dove Valley Practice, Worsbrough, United Kingdom: Catherine Liley; Walderslade Surgery, Barnsley, United Kingdom: Rebecca Wastling; The Rame Group Practice, Torpoint, United Kingdom: Paul McEleny; Dorking Medical Practice, Dorking, United Kingdom: Andre Beattie; Carryduff Surgery, Carryduff, United Kingdom: Philip Cooke; King Street Surgery, Lancaster, Lancaster, United Kingdom: Mike Wong; Park Road Surgery, Surrey, United Kingdom: Mark Pugsley; Roundwell Medical Centre, Norwich, United Kingdom: Chaminda Dooldeniya; Barton-on-Sea Surgery, New Milton, United Kingdom: Greg Rogers; Wareham Surgery, Dorset, United Kingdom: James Bennett; Wilton Health Centre, Salisbury, United Kingdom: Polly Jacobs; Leslie Medical Practice, Glenrothes, United Kingdom: Rajesh Muvva; Academy Medical Centre, Angus, United Kingdom: Matthew Adam; Bicester Health Centre, Bicester, United Kingdom: Robin Fox; Windrush Medical Practice, Witney, United Kingdom: Nicolas Thomas; The White Horse Medical Practice, Faringdon, United Kingdom: Simon Cartwright; Westongrove PMS Partnership, Aston Clinton, United Kingdom: Rory Reed; Park Terrace Medical Practice, Stirling, United Kingdom: Simon Randfield; The Broadshires Health Centre, Carterton, United Kingdom: Christine A'Court;

© 2020 Fox KAA et al. *JAMA Network Open.*

Kirkcaldy Health Centre, Kirkcaldy, United Kingdom: Ann Flynn; Rothes Medical Practice, Glenrothes, United Kingdom: Andrew Halpin; The Medical Centre, Redditch, United Kingdom: Shoeb Suryani; Clarence Medical Practice, Rhyl, United Kingdom: Simon Dobson; Plas Menai Surgery, Conway, United Kingdom: Louise Lomax; Plas Y Bryn Surgery, Wrexham, United Kingdom: Minnal Nadaph; Tryst Medical Centre, Stenhousemuir, United Kingdom: Iain Munro; Frome Valley Medical Centre, Frampton Cotterill, United Kingdom: Jane Goram; Kingswood Health Centre, Bristol, United Kingdom: Helen Stoddart; Charlotte Keel Health Centre, Bristol, United Kingdom: Phil Simmons; Llanedeyrn Health Centre, Llanedeyrn, United Kingdom: John Shewring; Chawton Park Surgery, Alton, United Kingdom: Emma Bowen-Simpkins; Park and St Francis Surgery, Valley Park, United Kingdom: Mark Rickenbach; Salisbury Medical Practice, Salisbury, United Kingdom: Polly Jacobs.

Australia: Lismore Base Hospital, Lismore, New South Wales, Australia: Adam Blenkhorn, Harry Gibbs, Leverne Hesketh, Janice Boys, Charmaine Morahan, and Tracey Mackney; Launceston General Hospital, Launceston, Tasmania, Australia: Bhuwanendu Singh, Carol Singh, Monica Campo, Rosemary Beveridge, Laurie McKeon, and Elizabeth Vardon; Pendlebury Research, Cardiff, New South Wales, Australia: Penny Astridge, Lyne Parsons, Lesley Carlton, and Geoffrey Oldfield; The Northern Hospital, Epping, Victoria, Australia: William van Gaal, It Meng Tsay, Vicki Lawlor, Chelsea Webster, Damien Cresp, Elizabeth Buckley, Rinku Rayoo, Mary Park, Uwais Mohamed, Naveen Sharma, Vivek Muhta, Elizabeth Vardon, and Amie Cho; The Canberra Hospital, Garran, Austl. Cap. Terr., Australia: Walter Abhayaratna, Emily Wilford, Katherine Johnson, Kate Hayes, Natasha Thomas, Renee Eslick, Mehdi Eskandari, and Ben Jacobson; Royal Hobart Hospital, Hobart, Tasmania, Australia: Philip Thomson, Vicki OMay, Nathan Dwyer, Catherine McIntosh, Lynette Reid, Teresa Grabek, Karen Patching, Andrew Black, Ben Costello, Rachel Lloyd, George Lukas, and Joseph Amin; Ashford Cardiac Clinic, Ashford, South Australia, Australia: Ron Lehman, Cameron Singleton, Hazel Morrison, Tanya Patching, Mandy Lehman, and Sam Lehman; Bankstown-Lidcombe Hospital, Bankstown, New South Wales, Australia: Jens Kilian, Marika Seremetkoska, Jo-Dee Myers, Deirdre Upton, Alla Waldman, Changjie Song, and Joanna Ramachenderan; Nepean Hospital, Kingswood, New South Wales, Australia: David Coulshed, Michael Fitzpatrick, Michele MacKenzie, Lisa Barry, George Touma, and Inaam Ullah; Royal Perth Hospital, Perth, Western Australia, Australia: Andrei Catanchin, Michelle Bonner, Claire Batta, Mary Vorster, Vincent Paul, Nikola Stoyanov, and Samantha Thompson; Core Research Group, Milton, Queensland, Australia: David Colquhoun, Aurelia Connelly, Lara Petelin, Dylan Barnes, Nigel Appleby, Tara Kinnane, Hilary Morrison, Antonio Ferreira-Jardim, and Bojana Petrovic; Macquarie University Hospital, North Ryde, New South Wales, Australia: Hosen Kiat, Lisa Wallis, Helena Setio, Anil Aggarwala, Kiran Swaraj, and Imran Kassam; Balwyn Consulting Suites, Epping, Victoria, Australia: David Eccleston and Karen Patching; Liverpool Hospital, Liverpool, New South Wales, Australia: John French, Maria Plotz, Craig Juergens, Suzanne Raynes, Natalia Sequalino, Kelsey O'Brien, Sally-Anne Hoddy, Tuan Nguyen, Christian Mussap, Hany Dimitri, Krishna Kadappu, Dominic Leung, and Alexandra Croucher; SA Heart, Ashford, South Australia, Australia: Bronte Ayres, Jessie Palmer, Jean Tarrant, Leon Zimmett, Tanya

© 2020 Fox KAA et al. *JAMA Network Open.*

Patching, and Marilyn Dolman; The Avenue Cardiovascular Centre, Dandenong, Victoria, Australia: Peter Blombery, Helen Rashad, and Claire McCarthy; Monash Medical Centre, Clayton, Victoria, Australia: Thanh Phan, Kitty Wong, and Lauren Sanders; Gosford Hospital, Gosford, New South Wales, Australia: James Rogers, Bets Conway, Jonathan Sturm, Margaret Webb, Veronica Zenteno, David Crimmins, Anna Schutz, and Susanne Rhodes; Heart Care Victoria - Heidelberg, Heidelberg, Victoria, Australia: David O'Donnell and Karen Patching; Pitt Town Family Practice, Pitt Town, New South Wales, Australia: Sang Cheol Bae; The Alfred Hospital, Melbourne, Victoria, Australia: Harry Gibbs and Vathy Nagalingam; Redcliffe Hospital, Redcliffe, Queensland, Australia: Patrick Carroll, Megan Ratcliffe, Maree Duroux, Samantha Shone, Johnathon Hunter, Mayank H Modi, and Richard Geraghty; Cairns Hospital, Cairns, Queensland, Australia: Greg Starmer, Shane Preston, Michelle Gosley, Sue Richmond, Sue Dixon, and Steven Sutcliffe; Lyell McEwin Hospital, Elizabeth Vale, South Australia, Australia: Margaret Arstall, Katrina Macmillan, Purendra Pati, Jann Parkinson, and Jane Rose; St George Hospital, Kogarah, New South Wales, Australia: Maurits Binnekamp, Jennifer Brimley, Ellie Watson, Prakriti Shrestha, and Megan Higgs; Cardiac Diagnostic Centre, Wollongong, New South Wales, Australia: Astin Lee, Stephen Mackay, Miyar Prathap Hegde, Taufik Fetahovic, Martin Walker, Joe Famia, and Janene Gibbs.

Canada: Hamilton General Hospital, Hamilton, Ontario, Canada: John Eikelboom, Sam Schulman, and Marlene Robinson; Oxford AIM Clinic, London, Ontario, Canada: Robert Luton, Shannen Douglass, Fraser Gibson, and Krystine Cooper; Brampton Research Associates, Brampton, Ontario, Canada: Milan Gupta, Ana Maria Sindilar, Lidia Lepore, Lekraj Nimraj, Mitra Mohammadi, and Ranjit Kahlon; Cambridge Cardiac Care Centre, Cambridge, Ontario, Canada: Amritanshu Shekhar Pandey, Michelle Pandey, and Linda Snell; Surrey Memorial Hospital, Surrey, British Columbia, Canada: Stephen Cheung, Jan Kornder, Lynn Breakwell, Tracy Cleveland, Jordana Largy, Alayna Ewert, and Shannon Wong; Leader, Rolland, Ajax, Ontario, Canada: Rolland Leader and Loris Aro; Dr Philippe Beaudry, Burlington, Ontario, Canada: Philippe Beaudry, Kathryn Beaudry, and Judy Dunnigan; Dr Philippe Beaudry, Montréal, Quebec, Canada: Celine Bergeron; Dr Philippe Beaudry, Downsview, Ontario, Canada: Gordhan Jethoo; CHUS–Hôpital Fleurimont, Sherbrooke, Quebec, Canada: Félix Ayala-Paredes, Caroline Lamoureux, Suzanne Maltais, Sonia Nadeau-Lapointe, Veronique Dagenais, Marie-Claude Grenier, and Claude Jean; JBN Medical Diagnostic Services Inc, Burlington, Ontario, Canada: Joseph Berlingieri and Fern Petrie; JBN Medical Diagnostic Services Inc, Windsor, Ontario, Canada: Stephen Lewis and Chris MacRae; Alder Medical Centre, Campbell River, British Columbia, Canada: John Heath, Linette Scott, and Jane Drown; Centre de santé et de services sociaux Champlain-Charles-Le Moyne, Greenfield Park, Quebec, Canada: Germain Poirier, Sandrine Spearson, Emilie Douville, Karine Primeau, Isabelle Neas, and Julie Gaudreault; The Medical Arts Health Research Group, Kamloops, British Columbia, Canada: Miranda du Preez, Patricia Roberts, Patti Roberts, Anna Kubanska, Judy Vicic, and Nathaly Reynoso; The Medical Arts Health Research Group, Powell River, British Columbia, Canada: Bradley Schweitzer, Timothy Barkowski, Stephen Burns, Sally Watson, and Patti Roberts; Centre Intégré Universitaire de Santé et de Services Sociaux du Nord-de-l'Île-de-Montréal, Montréal,

© 2020 Fox KAA et al. *JAMA Network Open.*

Quebec, Canada: Reginald Nadeau, Georgeta Sas, Zahra Djaidani, and Sadia Daheb; London Health Sciences Centre, London, Ontario, Canada: George Dresser, Donald Farquhar, Richard Kim, Rita Moor, Heather Vosper, Lara Whatmore, and Natalie Crown; Dhillon, Ripple, Oshawa, Ontario, Canada: Ripple Dhillon, Julie Bigcanoe, and Nanci Bignell; University of Alberta Hospital-SCC/WCM, Edmonton, Alberta, Canada: Tomasz Hruczkowski and Maria Raines; Regina General Hospital, Regina, Saskatchewan, Canada: Andrea Lavoie, William Semchuk, Payam Dehghani, Laurie Ferleyko, Cheryl Altwasser, and Sheila Kelly; Queen Elizabeth II Health Sciences Centre, Halifax, Nova Scotia, Canada: Ratika Parkash, Debbie Wright, Lisa Carroll, and Ann Fearon; James Cha, MD, Oshawa, Ontario, Canada: James Cha, Rebecca Otis, Judy Otis, and Elizabeth Burke; CHUM Hôtel-Dieu, Montreal, Quebec, Canada: Benoit Coutu, Denise Fournier, Julie Fleury, and Isabelle Denis; Nova Scotia Health Authority, Sydney, Nova Scotia, Canada: Paul MacDonald, Jeanie MacIsaac, Kathleen Hines, Annette Wood, Christine Hines, and Joy Howard; First Line Medical Services Ltd, St. John's, Newfoundland, Canada: Brian Ramjattan, Wayne Gulliver, Bonnie Clarke, and Teri Trahey; Fraser Clinical Trials Inc, New Westminster, British Columbia, Canada: Jorge Bonet, Dennis Rupka, Leanne Kwan, Katherine Haveman, Michael Kammermayer, and Jacquie Stevenson; Dr Saul Vizel Cardiac Research Office, Cambridge, Ontario, Canada: Saul Vizel and Beverley Fox; St. Michael's Hospital, Toronto, Ontario, Canada: Paul Angaran, Kamran Ahmad, Victoria Korley, Arnold Pinter, Paul Dorian, Iqwal Mangat, and Theresa Aves; Medicine Professional Corporation, Kitchener, Ontario, Canada: Sameh Fikry, Wagdy Basily, and Ana Maria Jackson.

Egypt: Private Clinic, Giza, Egypt: Hany Ragy and Annie Ohanissian; Private Clinic, Cairo, Egypt: Ahmed Mowafy; Private Clinic, Cairo, Egypt: Azza Katta; Private Clinic, Cairo, Egypt: Mazen Tawfik; Private Clinic, Alexandria, Egypt: Moustafa Nawar and Eman Zaatout; International Cardiac Center–I.C.C., Alexandria, Egypt: Mohamed Sobhy and Ahmed Soliman; Private Clinic, Cairo, Egypt: Seif Kamal Abou Seif; Private Clinic, Cairo, Egypt: Tarek Khairy; Private Clinic, Cairo, Egypt: Ahmed Abd El-Aziz and Michael Sobhy; Private Clinic, Al-Minya, Egypt: Nasser Taha; Private Clinic, Cairo, Egypt: Ashraf Reda, Nevein Sami, and Mohamed Reda; Private Clinic, Port-Said, Egypt: Atef Elbahry; Om Al Qura Cardiac Center, Tanta, Egypt: Mohamed Setiha, Sameh Samir, and Hanan Salem; Private Clinic, Assiut, Egypt: Mohamed Gamal El Din; Cardiotech for Medical Care, Giza, Egypt: Magdi Elkhadem and Ali Mohsen; Private Clinic, Cairo, Egypt: Adel El-Etreby, Shehab El Etriby, and Adham Abdel Tawab.

South Africa: Kettles, DI, East London, South Africa: David Kettles, Gerda Du Plessis, Debbie Browne, and Penny Jackson; Coronary Care Unit, Durban, South Africa: Junaid Bayat; Into Research, Pretoria, South Africa: Heidi Siebert, Craig Phillip Franklin, Petronella Nagel, and Maria Booysen; Dr AR Horak Private Practice, Cape Town, South Africa: Adrian Horak, Elwyn Lloyd, and Lindy Henley; Kelfkens, Y, Potchefstroom, South Africa: Ynez Kelfkens; Garda, RA, Johannesburg, South Africa: Riaz Garda, Soraya Cassimjee, and Loice Mavhusa; Charlotte Maxeke Johannesburg Academic Hospital Haematology Department, Johannesburg, South Africa: Barry Jacobson, Judy Sasto, Gladys Conway, Leonie Smith, Claire Cannon,

and Jeannie Marks; Dr Dawood, Mohamed and Pillay, Cape Town, South Africa: Thayabran Pillay and Margo Botha; Syzygy Clinical Research Services, Pretoria, South Africa: Michele Guerra, Lelanie van Zyl, Anna-Marie Stapelberg and Deidre Oosthuizen; Clinical Projects Research SA (PTY) LTD, Worcester, South Africa: Louis van Zyl, Marshall Heradien, Judy Anne Finlaison, Tania Ellis, and Francois van Zyl; Universitas Hospital Cardiology Research, Bloemfontein, South Africa: Hendrik Theron, Lorinda de Meyer, Ellen Makotoko, Marinda Karsten, and Andonia Page; Murray, AN, Cape Town, South Africa: Andrew Murray, Heather Christie, and Cecilia Boshoff; Winelands Medical Research Centre, Stellenbosch, South Africa: Rikus Louw, Garath Tarr, and Annalie Skein; Greyling, D, Cape Town, South Africa: Deon Greyling and Roelof Diederichs; Mntla, PS, Pretoria, South Africa: Pindile Mntla and Lillian Rikhotso; Ismail, SM, Cape Town, South Africa: Siddique Ismail, Anna Isaacs, Aziza Davids, Mariette Pelser, and Sipho Hlengwa; Ahmed, FA, Isipingo Rail, South Africa: Fayzal Ahmed and Charmaine Chami; Dr JM Engelbrecht Practice, Somerset West, South Africa: Johannes Engelbrecht, Marlene Mostert, and Wilhelmina Pretorius; Ramdass, AS, Durban, South Africa: Andrew Ramdass, Muhammed Moosa, and Indhranie Naidoo; Maharajh, S, Assagay, South Africa: Shambu Maharajh; Limaro Research, Bloemfontein, South Africa: Wessel Oosthuysen, Elizabeth Oosthuysen, and Cornel Bester; MERC–Cape Town, Cape Town, South Africa: Mohammed Moosa, Rehana Loghdey, Shireen Safodien, Mishka Salie, Farhaad Shaik, and Rosemary Matthews; Ueckermann, V, Pretoria, South Africa: Veronica Ueckermann and Andre Ueckermann.

United Arab Emirates: Sheikh Khalifa Medical City, Abu Dhabi, United Arab Emirates: Wael Al Mahmeed and Irfan Maqsood; Zayed Military Hospital, Abu Dhabi, United Arab Emirates: Abdullah Al Naeemi and Maher Makdad; Gulf Diagnostic Center Hospital, Abu Dhabi, United Arab Emirates: Ghazi Yousef, Ralph Manzano, Karen Magdaluyo, and Steve Teves; Dubai Hospital, Dubai, United Arab Emirates: Nooshin Bazargani and Azbin Abdul; Rashid Governmental Hospital, Dubai, United Arab Emirates: Munther AlOmairi and Amna Al Mulla; Zulekha Hospital LLC, Dubai, United Arab Emirates: Rajan Maruthanayagam, Abdul Rehman, and Preetha Haridas; Zulekha Hospital, Sharjah, United Arab Emirates: Rupesh Singh, Shereef El Bardisy, Sharmila Jadhav, and Preetha Haridas; Al Qassimi Hospital, Sharjah, United Arab Emirates: Ahmed Naguib; Kuwait Hospital, Sharjah, United Arab Emirates: Mohamed Ibrahim and Rehab Mohamed; Fujairah Hospital, Fujairah, United Arab Emirates: Amrish Agrawal and Jameelah Dominguez; RAK Hospital, Ras Al Khaimah, United Arab Emirates: Mukesh Nathani; Gulf Medical University Hospital, Ajman, United Arab Emirates: Ehab M. Esheiba, Nidhi Sharma, Mohammed Thanzeel, and Rahul Sharma; Ibrahim Bin Hamad Obaidullah Hospital, Ras-Al Khaimah, United Arab Emirates: Adel Wassef; Kalba Hospital, Sharjah, United Arab Emirates: Rajeev Gupta.

United States: Cotton-O'Neil Clinical Research Center, Topeka, Kansas, United States: Michael Cox and Erin Malone; Medical Associates of North Georgia, Canton, Georgia, United States: Scott Beach, Melanie Eley, April Brown, and Cheryl Verdi; FirstHealth of the Carolinas, Inc, Pinehurst, North Carolina, United States: Peter Duffy, Tim Richardson, Anne Dickerson, Valerie Palumbo, Shauna Eggertson, and

© 2020 Fox KAA et al. *JAMA Network Open.*

Pamela Mason; Southeast Clinical Research, LLC, Jacksonville, Florida, United States: Stephen Falkowski, Elizabeth Burkett, Tammy Parrott, and Karen Johnson; Montefiore Medical Center PRIME, Lake Success, New York, United States: Kevin Ferrick, Ronald Zolty, Sandy Congal, and Auris Browne; MCA Research, Houston, Texas, United States: Miguel Franco, Celine Garcia, Paula Gentry, and Melialoha Bartlett; Bryan Heart, Lincoln, Nebraska, United States: W. Michael Kutayli, Andrew Merliss, Rose Saalfeld, Cheryl Orosco, and Nicci Thompson; Truman Medical Centers, Inc, Kansas City, Missouri, United States: Annette Quick, Paramdeep Baweja, Daniel Pauly, M. Javed Ashraf, and Mickie Keeling; Gwinnett Hospital System, Inc, Lawrenceville, Georgia, United States: Niraj Sharma, Jowanna Kerr, and Marsha Headlee; Cardiology Consultants, Daytona Beach, Florida, United States: Vance Wilson, Terry Purcell, Jennifer Langdon, and Pamela Jones; Jean Brown Research, Salt Lake City, Utah, United States: Stephen Miller, Scott West, Heather Theobald, Kim Smith, Allison Jones, and Lorlie Evans; University of Texas Southwestern Medical Center, Dallas, Texas, United States: Mark Alberts, Patricia Knowles, and Jan Cameron-Watts; Central New Jersey Cardiology, South Plainfield, New Jersey, United States: Edwin Blumberg and Susan Paserchia; WellSpan Cardiology, Ephrata, Pennsylvania, United States: Roddy Canosa, Elise Hartranft, Susan Felpel, and Sarah Jasinski; Heart Specialists of Central Jersey, Freehold, New Jersey, United States: Ted Gutowski and Susan Karl; Community Health Care, Inc., Canal Fulton, Ohio, United States: Rodney Ison, Tammy Lincoln, Butcher Stephanie, and Paula Shaw; Cardiology Consultants LLP, Houston, Texas, United States: Jorge Garcia, Edward Massin, and Teresa Hicks; Coast Cardiovascular Consultants, PLLC, Biloxi, Mississippi, United States: Paul Mullen and Sherry Raziano; DM Clinical Research, Edison, New Jersey, United States: Howard Noveck, Fatema Hakimi, Debra Merritt, and Abbas Haideri; Baptist Heart Specialists, Jacksonville Beach, Florida, United States: Pamela Rama, Jill DePauw, and Doran Cassidy; T & R Clinic, Halton City, Texas, United States: Rajneesh Reddy and Melanie Bentley; The Valley Hospital, Fairlawn, New Jersey, United States: Marcus Williams and Jin Ah Lee; University of California Davis Health System, Sacramento, California, United States: Deborah Diercks, Daniel Nishijima, Laura Jones, Toni Harbour, Ben Mooso, Allyson Sage, Tirath Sanghera, and Michaela Canova; Tulane University Heart & Vascular Institute, New Orleans, Louisiana, United States: Keith Ferdinand, Patrice Delafontaine, Gholam Ali, Nana Asafu-Adjaye, and Suzanne Bowers; City Cardiology Associates, Akron, Ohio, United States: Ihsan Haque and Nicole Pickelsimer; Jamaica Hospital Medical Center, Jamaica, New York, United States: Robert Mendelson, Tazrin Tripti, Janaya Raynor, Genna Pearl, and Kelly Cervellione; University of Texas Health Science Center at Tyler, Tyler, Texas, United States: Sridevi Pitta, Kent Davis, Benji Hawkins, Michael Lay, Debbie Fielder, Christopher Herrick, and Jay Shoemaker; Washington University, Saint Louis, Missouri, United States: Daniel Theodoro, Kaharu Sumino, Gregory Ewald, Vanetta Worthy, Anne Thatcher, Lynn Henson, Kelly Ball, Catharine Richard, and Jessica Peterson; Cardiovascular Research Center of Knoxville, Atlanta, Georgia, United States: Charles Treasure, Candy Robertson, Michelle Parker, Lisa Treasure, and Joseph Minardo; SC Nephrology & Hypertension Center, Inc, Orangeburg, South Carolina, United States: Moustafa Moustafa and Samuel Emelife; Crystal Coast Family

© 2020 Fox KAA et al. *JAMA Network Open.*

Practice, P.A., Morehead City, North Carolina, United States: Cas Cader and Sommer Morton; Medication Management, LLC, Greensboro, North Carolina, United States: Walter Pharr, Katie Jenkins, Lauren Talton, Bryan Bray, Amy Riddle, and Jill Woody; Northwell Health, LLC, Lake Success, New York, United States: Alisha Oropallo, Farisha Baksh, and Sally Kaplan; George E. Platt, MD, Green Cove Springs, Florida, United States: George Platt, Margaret Thorne, Jeremy Love, and Kimberlee Harris; Augusta University, Augusta, Georgia, United States: Jaspal Gujral, Peggy Best, and Celestine Williams; AAHSRI, Annapolis, Maryland, United States: James Welker, Kathleen Gray, and Kristine Wood; Kentucky Lung Clinic, PSC, Hazard, Kentucky, United States: Firas Koura, Lori Akers, and Margie Duff-Coots.

© 2020 Fox KAA et al. *JAMA Network Open.*
